# Synthesis of α‐Hydroxy‐1,2,3‐Triazole‐linked Sialyltransferase Inhibitors and Evaluation of Selectivity Towards ST3GAL1, ST6GAL1 and ST8SIA2

**DOI:** 10.1002/cmdc.202400088

**Published:** 2024-07-01

**Authors:** Rémi Szabo, Chris Dobie, Andrew P. Montgomery, Harrison Steele, Haibo Yu, Danielle Skropeta

**Affiliations:** ^1^ School of Chemistry & Molecular Bioscience University of Wollongong Wollongong, NSW Australia; ^2^ Molecular Horizons University of Wollongong Wollongong, NSW Australia; ^3^ ARC Centre of Excellence in Quantum Biotechnology University of Wollongong Wollongong, NSW Australia

**Keywords:** sialyltransferase inhibitors, molecular modelling, antitumour agents, sialic acid, drug design

## Abstract

Tumour‐derived sialoglycans, bearing the charged nonulosonic sugar sialic acid at their termini, play a critical role in tumour cell adhesion and invasion, as well as evading cell death and immune surveillance. Sialyltransferases (ST), the enzymes responsible for the biosynthesis of sialylated glycans, are highly upregulated in cancer, with tumour hypersialylation strongly correlated with tumour growth, metastasis and drug resistance. As a result, desialylation of the tumour cell surface using either targeted delivery of a pan‐ST inhibitor (or sialidase) or systemic delivery of a non‐toxic selective ST inhibitors are being pursued as potential new anti‐metastatic strategies against multiple cancers including pancreatic, ovarian, breast, melanoma and lung cancer. Herein, we have employed molecular modelling to give insights into the selectivity observed in a series of selective ST inhibitors that incorporate a uridyl ring in place of the cytidine of the natural donor (CMP‐Neu5Ac) and replace the charged phosphodiester linker of classical ST inhibitors with a neutral α‐hydroxy‐1,2,3‐triazole linker. The inhibitory activities of the nascent compounds were determined against recombinant human ST enzymes (ST3GAL1, ST6GAL1, ST8SIA2) showing promising activity and selectivity towards specific ST sub‐types. Our ST inhibitors are non‐toxic and show improved synthetic accessibility and drug‐likeness compared to earlier nucleoside‐based ST inhibitors.

## Graphical abstract

Building on our previous research into 1,2,3‐triazole linked sialyltransferase inhibitors, we present the design and synthesis of a new series of compounds that improve upon the drug‐likeness of the parent scaffold. Additionally, we performed a preliminary SAR study with the STs hST3GAL1, hST6GAL1, and hST8SIA2. This work provides new insights into the design of selective ST inhibitors, particularly considering different nucleoside derivatives.

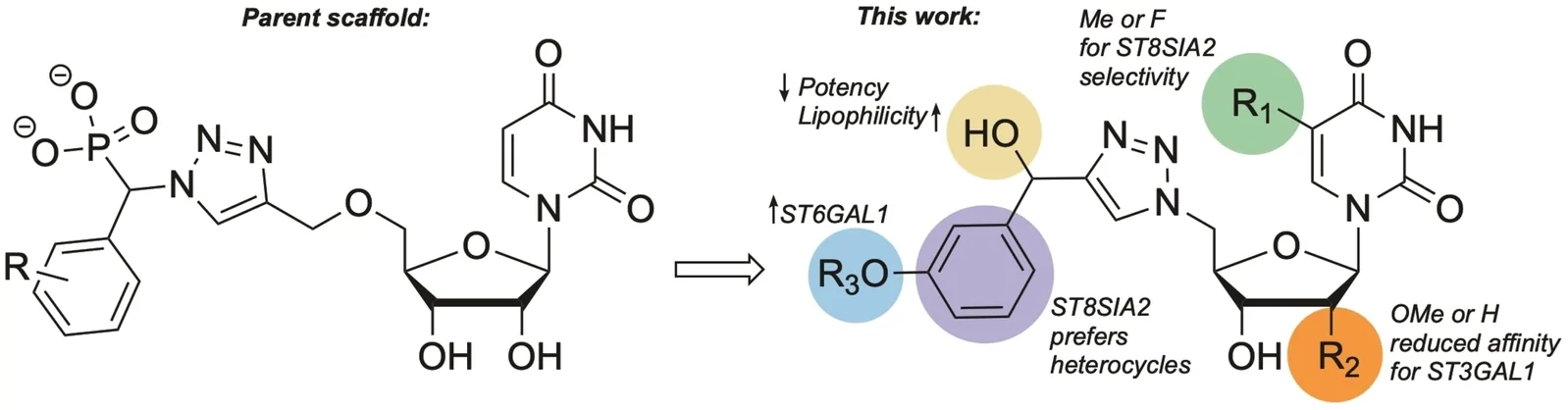

## Introduction

Sialic acid is the body's most important sugar next to glucose. It is found at the terminal position of many *N*‐ and *O‐*glycans where it directs cell‐cell communication including interactions with the extracellular matrix, immune cells, epithelial cells and antibodies.[^1^, ^2^] For this reason, tumour cells have hijacked this critical communication process, increasing their production of cell surface sialic acid (hypersialylation) to increase tumour aggressiveness and promote immune evasion, altered adhesion, metastasis and both chemo‐ and radioresistance.[^3^, ^4^] Multiple sialyl‐mediated mechanisms facilitate tumour cell survival including evading apoptosis and cell death via hypersialylation of the Fas receptor (FasR) and tumour necrosis factor receptor 1 (TNFR1) and altered adhesion and invasion via integrin‐mediated processes in numerous cancers including pancreatic, colon, breast, ovarian, melanoma and lung cancer.[^5^, ^6^, ^7^] Increased expression of sialylated selectin ligands on tumours is correlated to enhanced cancer metastasis, while circulating tumour cells also express sialylated ligands for E‐, P‐ and L‐selectins including sLe^a^, sLe^x^ and CD44.^[8]^ Hypersialylation of tumour‐derived glycans promotes binding to the sialic acid receptors expressed on immune cells (such as Siglecs) leading to immunosuppression, which can be reversed through desialylation.[^9^, ^10^, ^11^, ^12^, ^13^]

Hypersialylation is a hallmark of cancer^[14]^ and the basis of several biomarkers used clinically including the pancreatic cancer marker CA19‐9 (sLe^a^) and the sialylated mucin biomarkers CA125 (MUC16) and CA15‐3 (MUC1 analogue) for ovarian and breast cancer, respectively.^[15]^ The biosynthesis of sialylated glycoconjugates in humans is mediated by 20 different sialyltransferase (ST) enzymes that catalyse glycosylation between C2 of the sialyl donor cytidine monophosphate *N*‐acetylneuraminic acid (CMP‐Neu5Ac) and a C3, C6 or C8 hydroxyl of a glycan acceptor known as ST3, ST6 or ST8 subtypes accordingly.^[16]^ Sialyltransferases are further categorised by their acceptor, which is either galactose (Gal), *N*‐acetylgalactosamine (GalNAc) or another sialic acid (Sia) moiety. Tumour hypersialylation occurs via upregulation of STs (or downregulation of sialidases/neuraminidases) with oncogenes such as Ras and c‐Myc linked to increased ST transcription.[^3^, ^5^, ^17^, ^18^, ^19^, ^20^]

Due to the critical role of sialic acids in tumour metastasis and progression, inhibiting cell surface sialylation is a potential new cancer treatment being widely investigated.[^5^, ^20^, ^21^] Reported ST inhibitors range from soyasaponins, ginsenosides and lithocholic acid derivatives,[^22^, ^23^, ^24^] *N*‐acetylmannosamine derivatives as modified sialic acid biosynthetic precursors,[^25^, ^26^] analogues of cytidine,^[27]^ and the widely investigated peracetylated 3F_ax_‐Neu5Ac pioneered by Paulson and coworkers, which is converted intracellularly to the active inhibitor CMP‐3F_ax_‐Neu5Ac.[^28^, ^29^] This strategy of using a metabolic inhibitor has transformed the field of ST inhibition with the commercially available inhibitor widely used in ST inhibition studies in cancer cells and animal models.[^10^, ^30^, ^31^, ^32^] However, evaluation of the pan‐ST inhibitor in murine models has identified kidney and liver dysfunction as a limitation,^[33]^ highlighting the need for targeted delivery of a pan‐ST inhibitor or development of a selective ST inhibitor.

**Figure 1:**
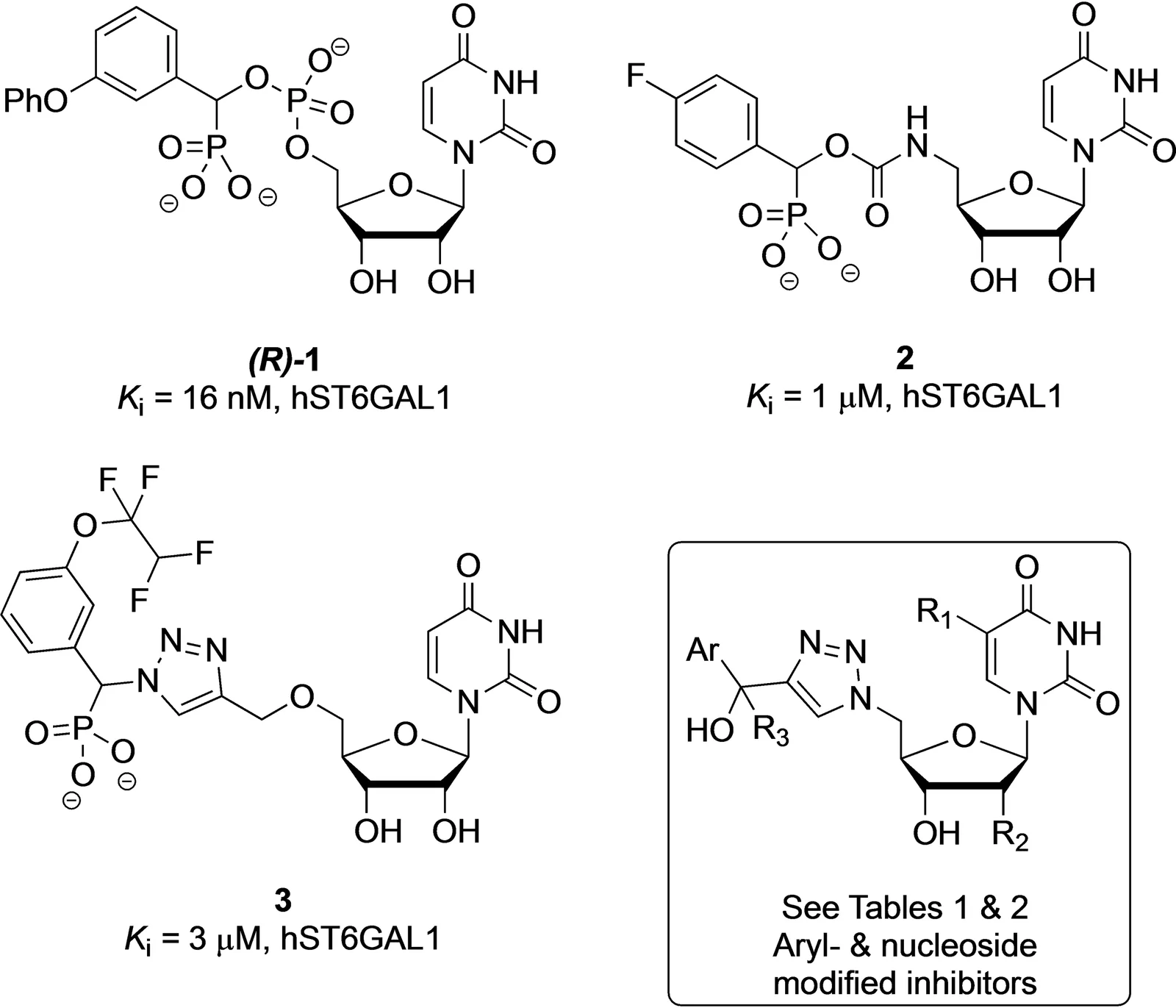
Structures of reported charged phosphodiester (**1**), carbamate (**2**), and 1,2,3‐triazole‐linked (**3**) transition‐state analogue sialyltransferase inhibitors, along with the newly developed neutral α‐hydroxy‐1,2,3‐triazole‐linked inhibitors.

The most successful ST inhibitors mimic the transition state of the ST‐catalysed transfer of sialic acid from the donor CMP‐Neu5Ac to the various acceptors.[^34^, ^35^] Examples such as the aryl‐based phosphodiester **1** (Figure 1) show excellent activity (*K_i_* =70 nM, rat a‐2,6‐ST; *K_i_* =19 nM, hST6GAL1**)**, where the aryl moiety mimics the sialic acid part and the phosphodiester mimics the phosphate/carboxylic acid arrangement in the natural donor.^[36]^ The phosphodiester **1** is one of the most potent agents reported and is widely used as a parent compound for ST inhibitor development. Numerous analogues of the CMP‐based ST inhibitors have been explored with a range of activities and selectivities for different ST subtypes.[^37^, ^38^, ^39^] Although phosphodiester‐based ST inhibitors (e. g. **1**) show potent nanomolar activity, there are potential bioavailability issues relating to their cell permeability, stability and susceptibility to phosphatase cleavage *in vivo*.^[40]^

**Figure 2:**
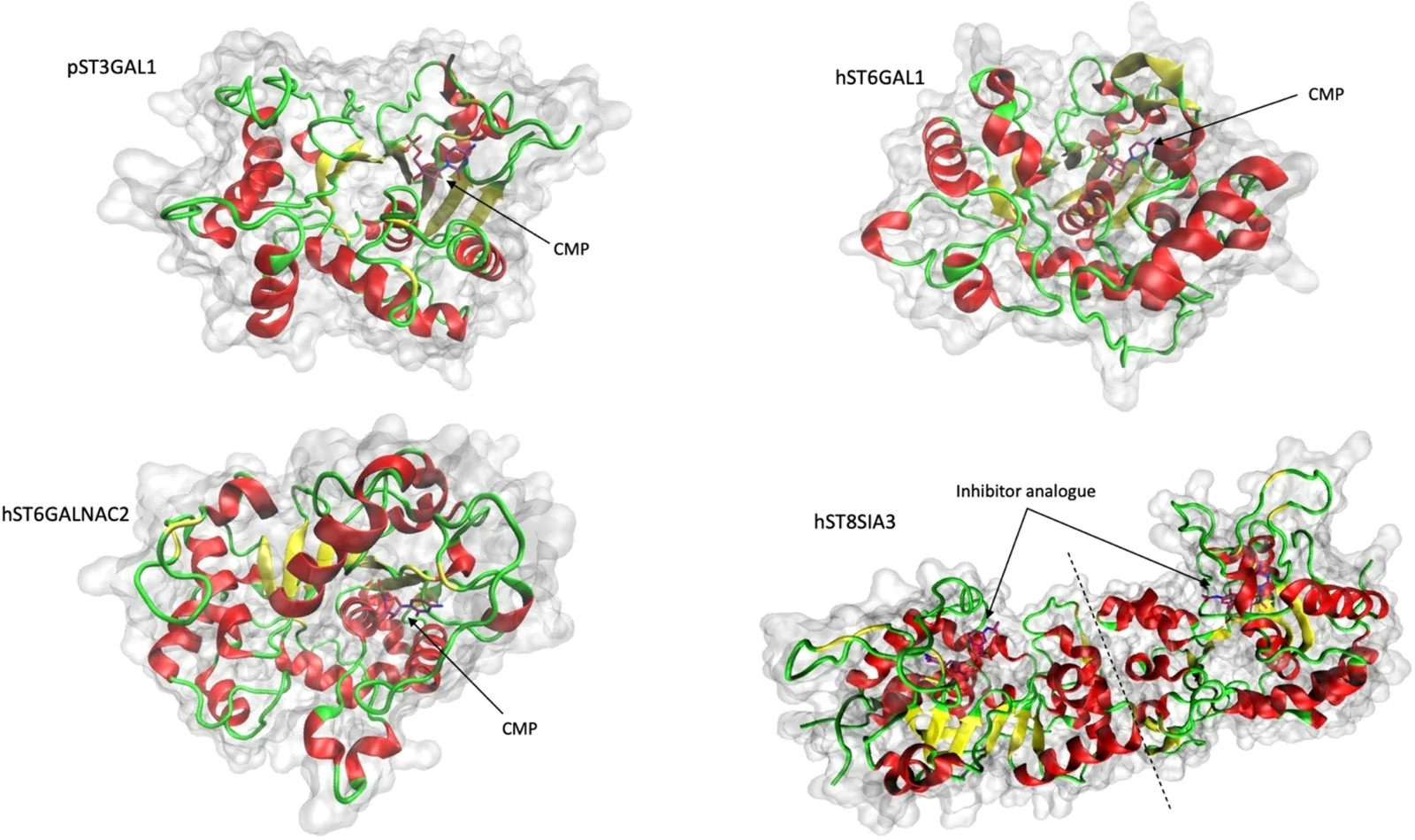
The X‐ray crystal structures of four mammalian sialyltransferase enzymes with donor analogue CMP bound: a) porcine ST3GAL1 [PDB: 4WNB];^[44]^ b) human ST6GAL1 [PDB: 4JS2];^[41]^ c) human ST6GALNAC2 [PDB: 6APL];^[43]^ and an inhibitor analogue bound to: d) human ST8SIA3 [PDB: 5CXY].^[42]^ These are the most fully resolved structures of these enzymes at the time of writing.

Crystallographic data has only been reported for three human STs (ST6GAL1,^[41]^ ST8SIA3^[42]^ and ST6GALNAC2^[43]^), along with the porcine ST3GAL1^[44]^ (Figure 2). The release of AlphaFold^[45]^ has enabled the evaluation of subtype selectivity across a wider number of ST subtypes for structure‐based design and optimisation, however, selective ST inhibition remains a challenge. To improve bioavailability, we have sought to replace the charged phosphodiester linker with an α‐hydroxy‐1,2,3‐triazole linker as a neutral bioisostere to improve linker stability, cell permeability and synthetic accessibility and using molecular modelling to guide substituent selection for greater ST subtype selectivity. We have previously demonstrated using docking and molecular dynamics (MD) simulations that triazole‐linked derivatives have comparable interactions to their phosphodiester‐linked counterparts within the hST6GAL1 (PDB ID: 4JS2) active site.[^46^, ^47^, ^48^, ^49^, ^50^] Herein, we present the synthesis, biological evaluation and molecular docking of synthetically accessible, small molecule sialylation inhibitors showing improved selectively towards different ST enzymes.

## Results and Discussion

### Chemistry

**Scheme 1:**
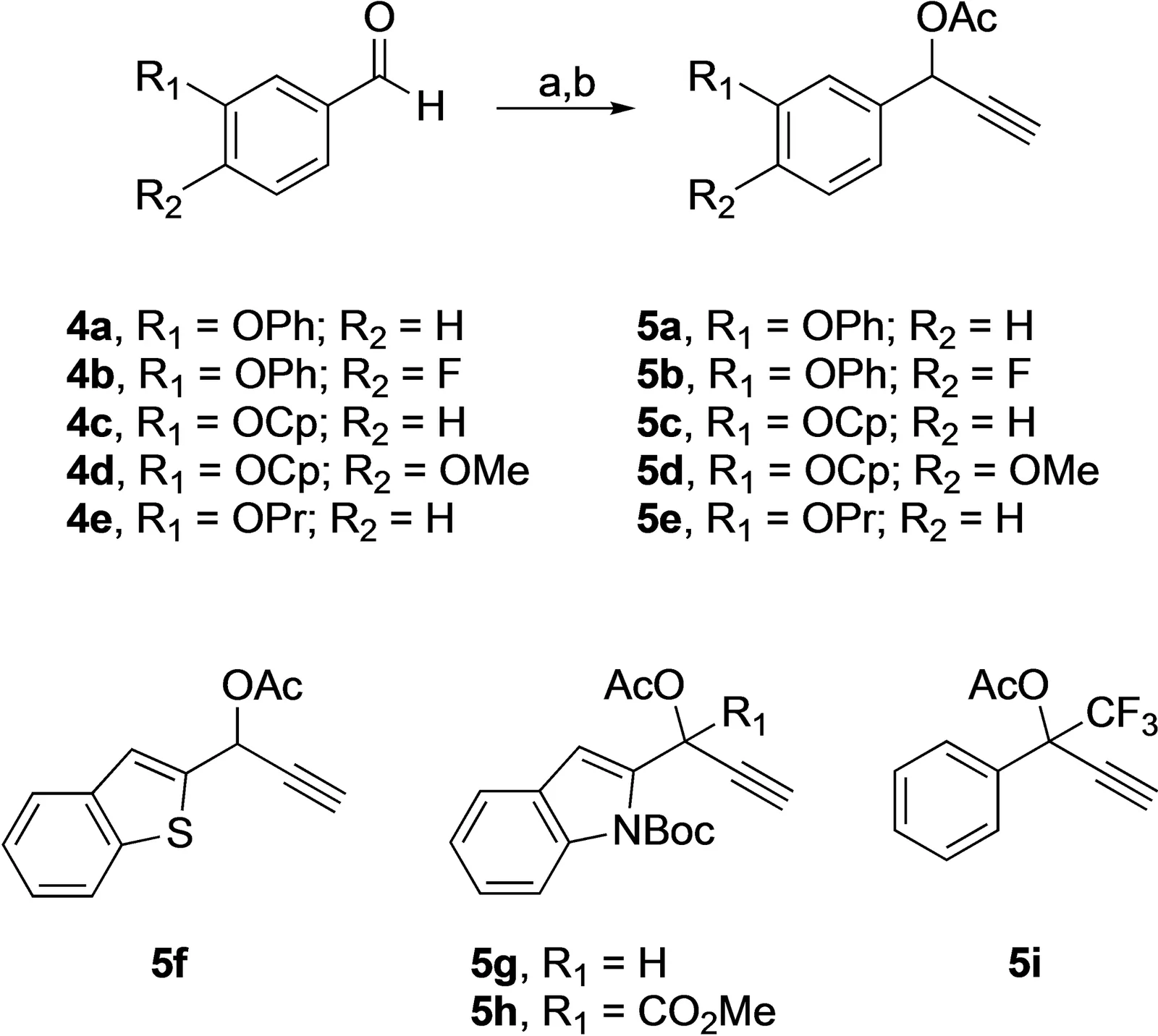
Synthesis of the α‐arylpropargyl acetate derivatives **5** **a**–**e** from the corresponding aldehydes. *Conditions*: (a) Ethynyl‐MgBr (1.2 equiv.), THF, 0 °C. (b) Ac_2_O (1.2 equiv.), Et_3_N (1.5 equiv.), CH_2_Cl_2_, r.t, 83–94 % yield over two steps. Heterocyclic derivatives **5** **f–I** were prepared analogously in 51–86 % yield (see Supplementary Information, page 3). Note: Cp=cyclopentyl, Pr=*n*‐propyl.

Classical ST inhibitors, such as compound **1**, are based on a highly charged, phosphodiester scaffold.^[36]^ To improve their stability and accessibility, we have previously reported the development of carbamate‐ (**2**) and triazole‐linked (**3**) uridyl‐based ST inhibitors showing low micromolar activity against hST6GAL1 (Figure 1).[^51^, ^52^] To further promote cell permeability, herein, we have sought to replace the charged phosphonate moiety in these promising earlier ST inhibitors with a neutral hydroxy group to retain some of the key contacts in the active site. To prepare the target triazole‐linked ST inhibitors, we used the Cu(I)‐catalysed azide‐alkyne cycloaddition (CuAAC) as the key coupling step within a convergent synthetic pathway,^[53]^ combining an appropriately protected 5′‐azido‐5′‐deoxynucleoside with an α‐arylpropargyl alcohol or acetate building block. This method provides a convenient way to form a triazole ring under mild conditions. The alkyne fragment was prepared in two steps by the addition of ethynylmagnesium bromide to a range of benzaldehyde derivatives (**4** **a**–**e**) followed by acetylation.^[54]^ Following this method, a series of 3‐ and 3,4‐substituted benzyl alkynes **5** **a–e** were obtained in high yields (83–92 %, Scheme 1, see supplementary section for synthesis details). The alkyne‐bearing 2‐benzothiophene (**5** **f**) and 3‐indole (**5** **g** and **5** **h**) building blocks were prepared similarly. Under the above conditions, benzothiophene‐2‐carboxaldehyde gave **5** **f** in 86 % yield. The addition of ethynylmagnesium bromide to the indolic derivatives required the protection of the *N* ^1^‐position on the starting material. Thus, indole‐3‐carboxaldehyde and methyl 2‐(1*H*‐indol‐3‐yl)‐2‐oxoacetate were treated with di‐*tert‐*butyl dicarbonate and 4‐dimethylaminopyridine in acetonitrile^[55]^ before the addition of the Grignard reagent to afford the corresponding alkynes **5** **g** and **5** **h** (73 % and 51 %, Scheme 1). The α‐trifluoromethyl alkynyl compound **5** **i** was produced analogously in 89 % yield starting from commercially available 1,1,1‐trifluoroacetophenone.

**Scheme 2:**
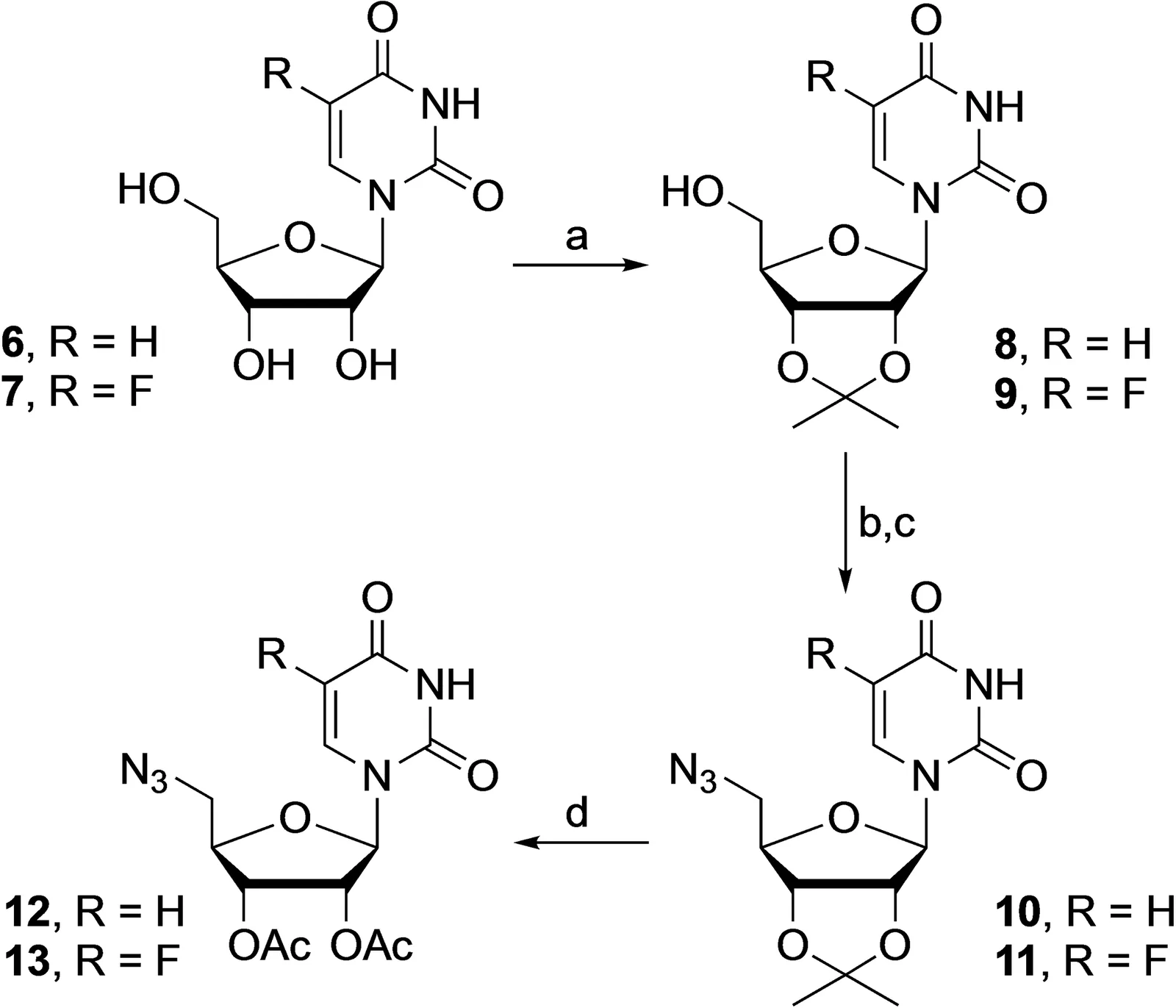
Synthesis of the uridine and 5‐fluorouridine 5′‐azido‐5′‐deoxy derivatives **12** and **13**. *Conditions*: (a) acetone, H_2_SO_4_, r.t., 99 %; (b) MsCl (1.2 equiv.), Et_3_N (2 equiv.), CH_2_Cl_2_, 0 °C – r.t., 91 %; (c) NaN_3_ (2 equiv.), DMF, 80 °C, 86 %; (d) i) In(OTf)_3_ (5 mol %), CH_3_CN/H_2_O [4 : 1], reflux; (ii) Ac_2_O (excess), 0 °C – r.t., 92 %.

A series of 5′‐azido‐5′‐deoxynucleosides were prepared from commercially available thymidine/uridine derivatives. First, the acetyl‐protected 5′‐azido‐5′‐deoxyuridine and 5‐fluorouridine building blocks (Scheme 2) were prepared using the method of Kamien *et al*.^[56]^ The synthesis commenced by selectively protecting the 2′,3′‐diol of uridine (**6**) and 5‐fluorouridine (**7**) to yield the corresponding acetonide‐protected compounds **8** and **9**, respectively. The free primary alcohols were then treated with methane sulfonyl chloride and pyridine, followed by substitution with sodium azide in DMF to give the corresponding azides (**10** and **11**) in high yields. The acetonide protecting group was removed using indium(III) triflate and the resultant 2′,3′‐diol protected with acetate groups via treatment with acetic anhydride to give the diacetylated 5′‐azido‐5′‐deoxyuridine derivatives **12** and **13**, also in excellent yields.

**Scheme 3:**
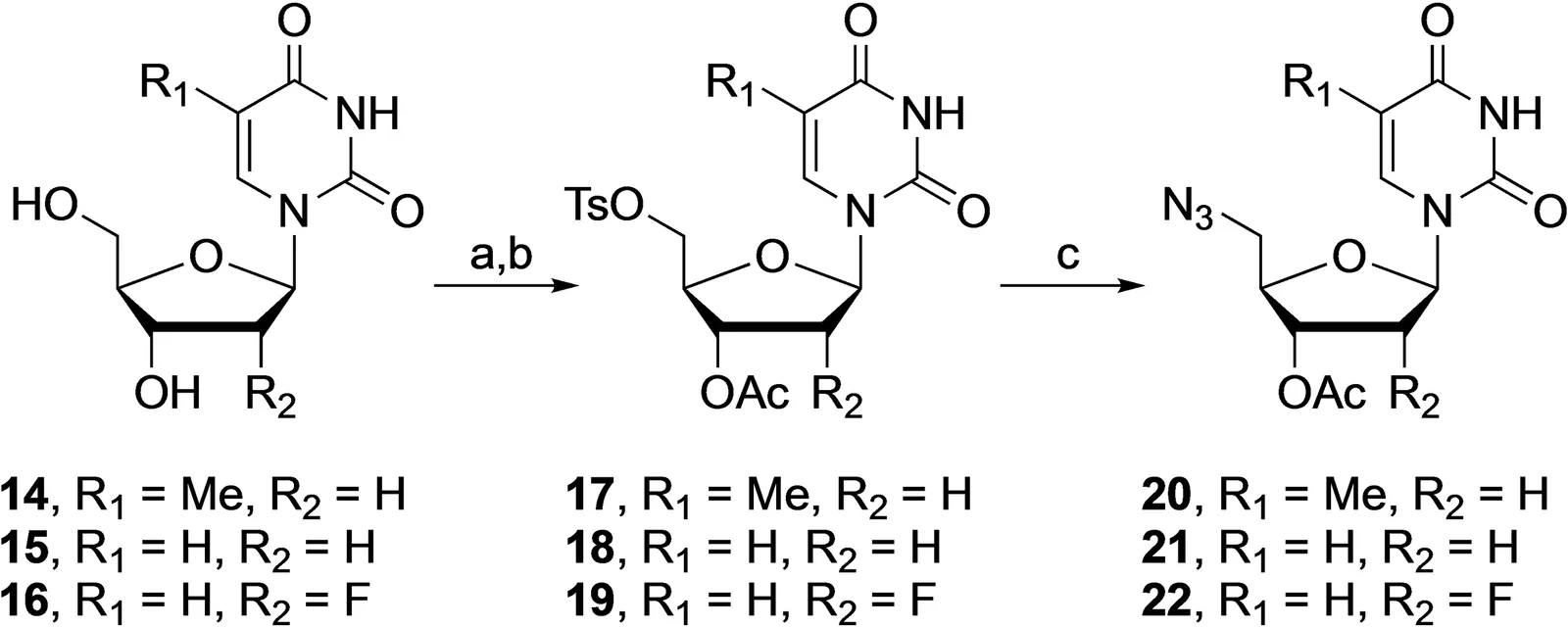
Synthesis of the thymidine, 2′‐deoxyuridine, and 2′‐deoxy‐2′‐fluorouridine 5′‐azidonucleosides **20–22**. *Conditions*: (a) TsCl (1.1 equiv.), pyridine, −10 °C; (b) Ac_2_O (excess), 0 °C – r.t., 47–62 % yield (two steps); (c) NaN_3_ (2 equiv.), DMF, 80 °C, 89–93 % yield.

The thymidine and 5′‐azido‐2′,5′‐dideoxyuridine building block derivatives were prepared by *one‐pot* tosylation of the starting nucleosides **14–16** followed by acetylation in pyridine at 0 °C.^[57]^ The resulting protected 5′‐tosylnucleosides **17–19** were then treated with sodium azide in DMF at 80 °C (Scheme 3) to give the target azido click coupling partners **20–22** in good overall yields. This method was also trialled for the 2′‐*O‐*methyluridine analogue (**23**), however, these conditions provided a mixture of diastereomers at the C1′‐position. Therefore, an alternate method was developed for this compound.

**Scheme 4:**
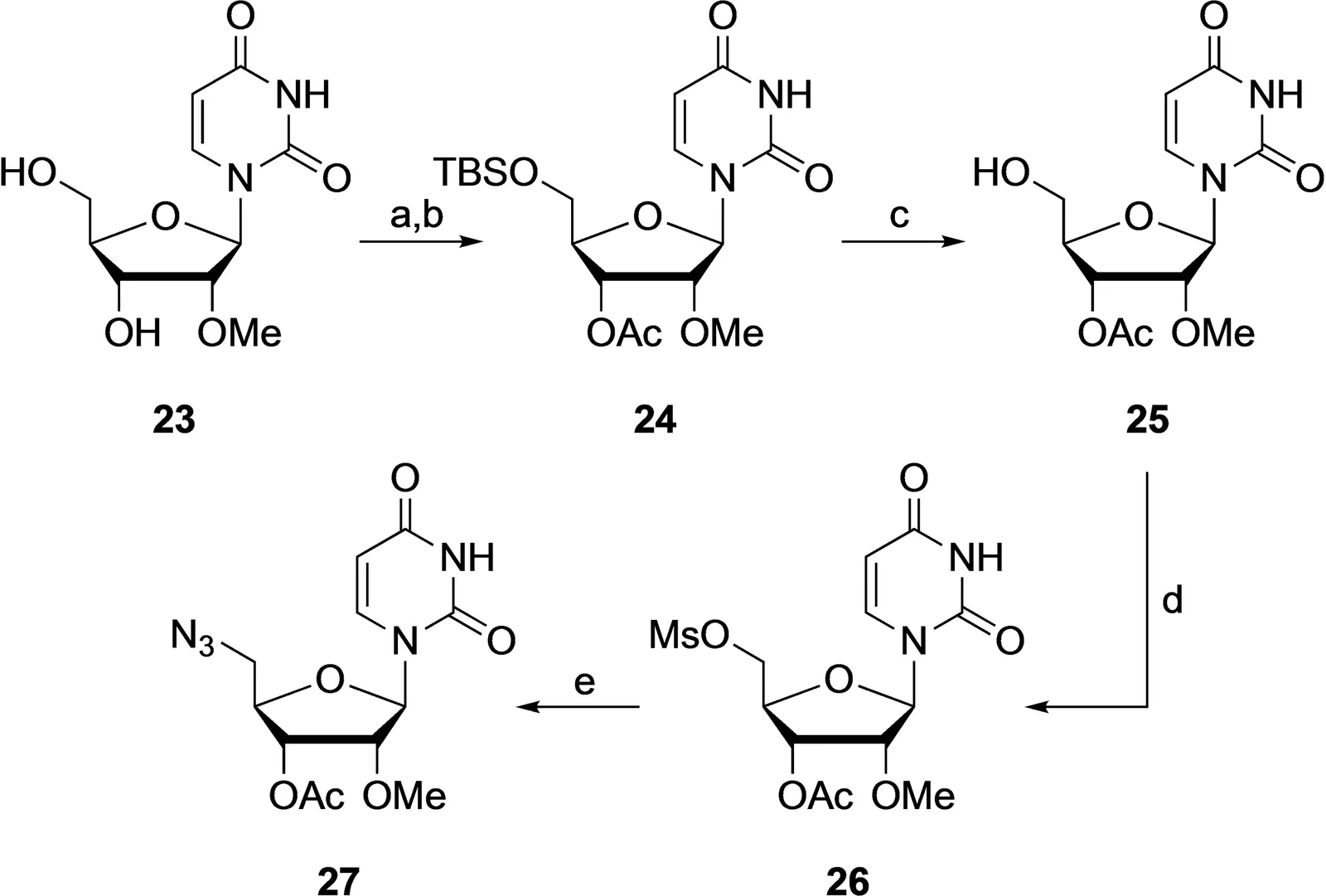
Synthesis of 3′‐O‐acetyl‐5′‐azido‐5′‐deoxy‐2′‐methoxyuridine **27**. Conditions: (a) TBDMSCl (1.1 equiv.), imidazole (2.5 equiv.), DMF, r.t.; (b) Ac_2_O (excess), 0 °C, 74 % (two steps); (c) TBAF (1.2 equiv.), AcOH (1.5 equiv.), THF, 0 °C, 76 %; (d) MsCl (1.2 equiv.), Et_3_N (1.5 equiv.), CH_2_Cl_2_, 0 °C, 72 %; e) NaN_3_ (2 equiv.), DMF, 80 °C, 91 %.

The 5′‐azido‐5′‐deoxy‐2′‐methoxyuridine (Scheme 4) building block was successfully obtained in five steps by selectively protecting the 5′‐position of 2′‐methoxyuridine (**23**) with a bulky TBDMS group followed by acetylation of the 3′‐hydroxyl group to yield the fully protected derivative **24** in 74 % over two steps. Cleavage of the silyl ether with TBAF in THF gave the free primary alcohol (**25**), suitable for reacting with mesyl chloride (**26**) in good yield, followed by treatment with sodium azide to afford the desired azido product **27** in high yield. Alternatively, this compound could be prepared using the method described by Wang *et al*., which utilises carbon tetrabromide, sodium azide, and triphenylphosphine.^[58]^

Next, the key alkynyl (Scheme 1) and azido (Schemes 2, 3, 4) fragments were coupled via a CuAAC reaction (Table 1). The synthesis of 1,4‐substituted‐1,2,3‐triazoles involves the *in situ* generation of Cu(I) catalyst from Cu(OAc)_2_ and sodium ascorbate as a reducing agent to facilitate the CuAAC reaction.^[53]^ A variety of solvent systems were screened, and it was found that a mixture of acetonitrile and water [4 : 1] was optimal for these reactions. In the examples presented, rapid completion of the reaction in good yield directly correlated with the substrates’ solubility. As shown in Tables 1 and 2, these conditions were appropriate for all the nucleoside derivatives utilised in this study (**12**, **13**, **20**, **21**, **22** and **27**).

**Table 1:** Synthesis of aryl‐modified sialyltransferase inhibitor derivatives **29** **a–i** (reagents and conditions in table footnotes) and inhibition against hST3GAL1, hST6GAL1 and hST8SIA2 at 100 μM.

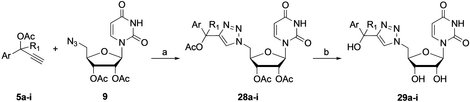								
Entry	Alkyne	Ar	R_1_	Click product Yield (%)	Deprotection Yield (%)	% Inhibition of **29** **a–i** at 100 μM		
						hST3GAL1	hST6GAL1	hST8SIA2
1	**5** **a**	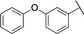	H	**28** **a**: 84	**29** **a**: 93	43	84	45
2	**5** **b**	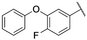	H	**28** **b**: 87	**29** **b**: 89	64	63	93
3	**5** **c**	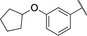	H	**28** **c**: 89	**29** **c**: 92	n.t.^[a]^	n.t.^[a]^	42
4	**5** **d**	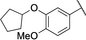	H	**28** **d**: 84	**29** **d**: 90	27	57	35
5	**5** **e**	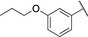	H	**28** **e**: 90	**29** **e**: 94	27	57	24
6	**5** **f**	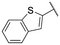	H	**28** **f**: 92	**29** **f**: 94	n.t.^[a]^	n.t.^[a]^	69
7	**5** **g**	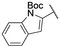	H	**28** **g**: 90	**29** **g**: 83	47	51	87
8	**5** **h**	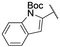	CO_2_Me	**28** **h**: 81	**29** **h**: 79	45	73	96
9	**5** **i**	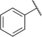	CF_3_	**28** **i**: 97	**29** **i**: 86	n.t.^[a]^	n.t.^[a]^	30

*Reagents and Conditions*: (a) Cu(OAc)_2_ (0.25 equiv.), sodium ascorbate (0.5 equiv.), ACN/H_2_O [4 : 1], r.t., 81–97 %; (b) NH_4_OH, MeOH, r.t., 79–94 %. [a] n.t.=not tested.

**Table 2:** Synthesis of nucleoside‐modified sialyltransferase inhibitor derivatives **31** **a–e** (reagents and conditions in table footnotes) and inhibition against hST3GAL1, hST6GAL1 and hST8SIA2 at 100 μM.

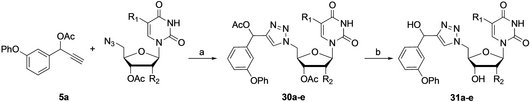								
Entry	Azide	R_1_	R_2_	Click product Yield (%)	Deprotection Yield (%)	% Inhibition of **31** **a–g** at 100 μM		
						hST3GAL1	hST6GAL1	hST8SIA2
1	**13**	F	OAc/OH^[a]^	**30** **a**: 96	**31** **a**: 98	<1	4	86
2	**20**	Me	H	**30** **b**: 94	**31** **b**: 97	1	<1	52
3	**21**	H	H	**30** **c**: 85	**31** **c**: 92	1	56	80
4	**22**	H	F	**30** **d**: 94	**31** **d**: 96	44	55	53
5	**27**	H	OMe	**30** **e**: 83	**31** **e**: 95	4	64	68

*Reagents and Conditions*: (a) Cu(OAc)_2_ (0.25 equiv.), sodium ascorbate (0.5 equiv.), ACN/H_2_O [4 : 1], r.t., 83–96 %; (b) NH_4_OH, MeOH, r.t., 92–98 %. [a] R_2_=OAc for the protected compound **30** **a** and OH for the fully deprotected compound **31** **a**; [b] n.t.=not tested.

In order to remove the acetyl protecting groups, the triazole compounds **28** **a**–**i** and **30** **a**–**e** were taken up in methanol and treated with concentrated ammonia to provide the corresponding final targets **29** **a**–**i** and **31** **a**–**e** in high yields (Tables 1 and 2). Interestingly, the methyl ester group on **28** **h** was kept intact under these conditions to yield the final indole compound **29** **h**.

The final compounds were synthesised as diastereomers that were not readily separable by HPLC and, therefore, tested for inhibitory activity as racemic mixtures. In previously reported ST inhibitors the diastereomers did not vary significantly in inhibitory activity against hST6GAL1.[^51^, ^52^] Where required, the lead compounds from this study will be prepared via asymmetric synthesis^[36]^ for additional *in vitro* and *in vivo* assessment as these compounds progress further.

### Sialyltransferase Inhibition

Sialyltransferase inhibition was assessed for hST3GAL1 and hST6GAL1 via the CMP‐Glo enzymatic assay utilised in our previous work. This method determines ST activity based on CMP production, which is detected via luminescence produced from a luciferase‐mediated reaction.[^51^, ^52^] Inhibition of hST8SIA2 was assessed by a reported HPLC‐based method using a fluorescently labelled polysialic acid acceptor (DMB‐DP3) as described by Keys *et al*.^[59]^

Of the aryl‐ and hetaryl modified series (**29** **a–i**, Table 1), the best performing derivative against ST3GAL1 was the 3‐phenoxy‐4‐fluoro compound **29** **b**, with 64 % inhibition at 100 μM (Table 1; Entry 2), while the indole derivatives **29** **g** and **29** **h** showed comparative activity to the 3‐phenoxy compound **29** **a** (47, 45, and 43 % inhibition respectively, Table 1; Entries 1, 7 and 8). For ST6GAL1, the most promising compound remained the 3‐phenoxy substituted compound **29** **a** (84 %, Table 1; Entry 1), while the indole compound with a methyl ester on the chiral carbon (**29** **h**) also showed good activity (73 %, Table 1; Entry 8). This result correlates with the findings of other ST inhibitor series, where H‐bond acceptors (such as a charged phosphonate group) in this position, along with an extended bond length from the chiral carbon, greatly improve activity against ST6GAL1.[^51^, ^52^] The results against ST8SIA2 appear to follow a combination of the trends of the other two subtypes, where the 3‐phenyoxy‐4‐fluoro derivative **29** **b** and both indole compounds **29** **g** and **29** **h** performed well (93, 87, and 96 % inhibition, respectively, Table 1; Entries 2, 7 and 8).

For the nucleoside‐modified derivatives, the uridyl (**29** **a**, 43 %, Table 1; Entry 1) and the 2′‐fluoro‐2′‐deoxyuridine derivative (**31** **d**, 44 %, Table 2; Entry 4) were active against ST3GAL1, with all other modifications at the 5‐position of the pyrimidine ring and the 2′‐position of the ribose ring reducing activity towards this ST subtype. For ST6GAL1, the uridyl parent compound (**29** **a**) was the most active at 84 % inhibition (Table 1; Entry 1), followed by the 2′‐*O*‐methyluridine derivative (**31** **e**, 65 %, Table 2; Entry 5), 2′‐deoxyuridine (**31** **c**, 56 %, Table 2; Entry 3) and 2′‐fluoro‐2′‐deoxyuridine derivative (**31** **d**, 55 %, Table 2; Entry 4). The 5‐fluorouridine derivative (**31** **a**) was the most active towards ST8 at 86 % inhibition (Table 2; Entry 1), with both the 5‐fluorouridine (**31** **a**) and thymidine derivative (**31** **b**) showing selectivity towards ST8 over ST3 and ST6 (both compounds with <5 % inhibition against both ST3 and ST6, Table 2; Entries 1 and 2). For ST8, the next most active compounds after 5‐fluorouridine (**31** **a**) were 2′‐deoxyuridine (**31** **c**, 80 %, Table 2; Entry 3) and the 2′‐*O*‐methyluridine derivatives (**31** **e**, 68 %, Table 2; Entry 5), noting that both compounds also showed significant activity towards ST6, but no activity towards ST3. From this series, all six compounds were active (45–86 %) against ST8, four were active towards ST6 and two against ST3.

Regarding selectivity, it appears that ST8SIA2 is more promiscuous than the other two ST subtypes tested. In the aromatic region, this finding could be explained by the enzyme's preference for polysialic acids,^[42]^ which could mean that it has a more accommodating acceptor region. On the other hand, aliphatic substituents, as in the 3‐cyclopentoxy‐4‐methoxy (**29** **d**) and 3‐propoxy (**29** **e**) derivatives, seem to afford mild selectivity for ST6GAL1 (Table 1; Entries 4 and 5), a finding supported by the results from our investigation of ether‐triazoles as ST inhibitors.^[51]^ A highlight in the study was the finding that modification at the 5‐position of the pyrimidine ring as in the 5‐fluorouridine (**31** **a**) and thymidine (**31** **b**) derivatives gives selectivity for ST8SIA2, while eliminating activity against ST3GAL1 and ST6GAL1 (Table 2; Entries 1 and 2). This observation provides an opportunity to develop highly selective inhibitors for the ST8 subtype that are critical in several cancers and neurological disorders.[^5^, ^21^, ^50^]

All final compounds were evaluated in an MTS cell viability assay using a MiaPaCa‐2 pancreatic cancer cell line. While MiaPaCa‐2 cells are known to express ST6GAL1,^[6]^ the purpose of this assay was a preliminary evaluation of cytotoxicity therefore it was not required for the cell line to express all three ST subtypes. The compounds showed no reduction in cell viability when tested at concentrations of 200–300 μM for an incubation period of 24–48 hours. This is in line with non‐cytotoxic effects reported previously for ST inhibitors.^[52]^ *In silico* evaluation with ProTox3 (https://tox.charite.de/protox3) also gave no predicted cytotoxicity for all final compounds (see Supplementary section). Further cell‐based studies evaluating cytotoxicity and anti‐metastatic activity on a range of both normal and cancer cell lines will be reported in the future. All compounds were also assessed using SwissADME (www.swissadme.ch) as being free from any pan‐assay interference compounds (PAINS) as described by Baell *et al*.^[60]^ The ADME parameters and PK properties were also computed for the final compounds with SwissADME and compared to their phosphodiester,^[36]^ carbamate,^[52]^ and ether‐triazole^[51]^ counterparts, indicating improved properties including reduced size, H‐bond acceptors, lipophilicity, molar refractivity and polarity, along with greater synthetic accessibility (see Table S1, Supplementary Information).^[61]^

### Molecular Docking

Docking simulations of a selection of the most promising inhibitors were conducted in the crystal structure of the human ST6GAL1 (PDB: 4JS2),^[41]^ ST8SIA3 (PDB: 5CXY)^[42]^ and porcine ST3GAL1 (PDB: 2WNB)^[44]^ using AutoDock Vina, according to the methods described previously.[^47^, ^48^, ^49^, ^51^, ^52^] For each simulation, the scores corresponding to conformations aligning with CMP from the crystal structure were considered. Although pST3GAL1 and hST8SIA3 differ to the enzyme sub‐type employed in the inhibitor evaluation studies, they are the closest available crystal structures to hST3GAL1 and hST8SIA2, respectively. There is 85 % homology between pST3GAL1 and hST3GAL1 and 39 % homology between hST8SIA2 and hST8SIA3, with each pair sharing functionally homologous domains.^[62]^ For this reason, this docking study was performed using these structures to further aid the design of ST inhibitors by providing insights into the selectivity observed between enzymes for different inhibitors.

**Figure 3:**
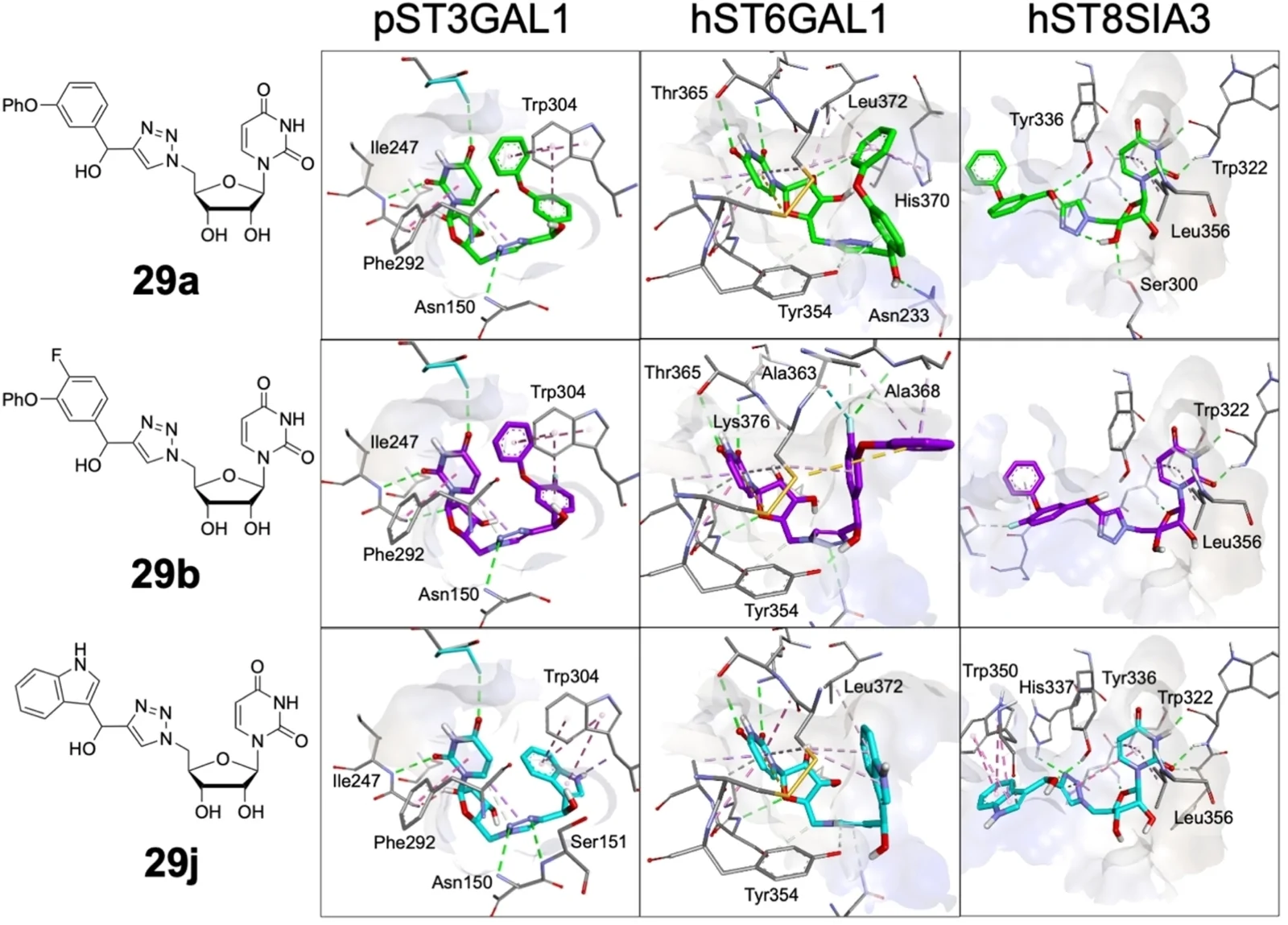
Docking of the *aryl‐modified uridine derivatives* **29** **a**, **29** **b**, and **29** **j** into the X‐ray diffraction structures for porcine ST3GAL1 [PDB: 2WNB], human ST6GAL1 [PDB: 4JS2] and human ST8SIA3 [PDB: 5CXY].

**Figure 4:**
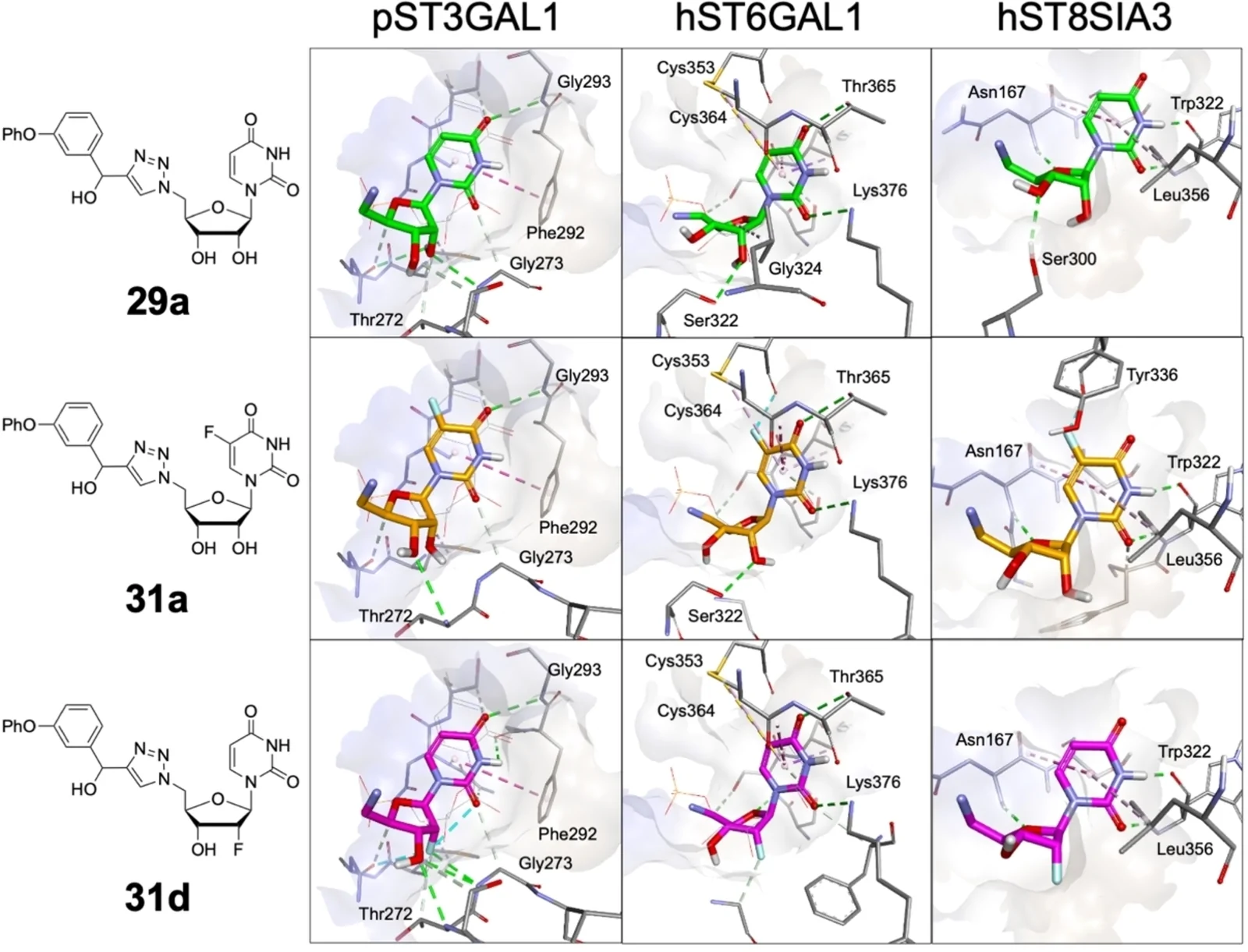
Docking of the *nucleoside‐modified uridine derivatives* **29** **a**, **31** **a**, and **31** **d** into the X‐ray diffraction structures for porcine ST3GAL1 [PDB: 2WNB], human ST6GAL1 [PDB: 4JS2] and human ST8SIA3 [PDB: 5CXY]. The triazole and aromatic region of each compound has been made transparent for visibility of the nucleoside fragment and is presented at a different view to those in Figure 3 for optimal visualisation of the nucleoside.

Five triazole compounds of variable activity between the three ST subtypes were chosen to probe the structure‐activity relationships. To assess the effects of the different aromatic groups on ligand‐receptor interactions in the ST enzymes, the aryl‐modified derivatives **29** **a** (3*‐*phenoxybenzyl) and **29** **b** (3‐phenoxy‐4‐fluorobenzyl) were chosen, along with **29** **j**, which is the deprotected version of the Boc‐protected 3‐indole compound **29** **g** (Figure 3 and Table 1). To assess the impact of modifying the nucleoside component, the uridine derivative **29** **a**, 5‐fluorouridine derivative **31** **a** and 2′‐fluoro‐2′‐deoxyuridine derivative **31** **d** were chosen for the molecular docking experiments (Figure 4). Images of the best and most consistent binding modes are shown in Figures 3 and 4, with the *R* diastereomer used for each compound for consistency.

For ST3GAL1, while not explicit in these docking poses, it appears that the presence of a fluorine atom on the aromatic fragment contributes to increased affinity. For example, the fluorinated derivative **29** **b** (Figure 3) shows the highest inhibitory activity towards ST3GAL1 (Table 1, Entry 2), possibly through a different binding conformation to that shown, allowing fluorine to interact with hydrophobic residues in that binding pocket. On the nucleoside fragment, the 5‐position on the uridine ring appears to be important for activity. The 5‐fluorouridyl substitution present in **31** **a** (Figure 4) reduced the activity of this derivative (Table 2, Entry 1), which is consistent with modelling of the active site surface as relatively hydrophilic and thus having a fluorine (**31** **a**) or methyl (**31** **b**) in this space would be unfavourable. There is, however, negligible difference in activity when the 2′‐hydroxyl moiety in **29** **a** is substituted with fluorine (**31** **d**), as seen by comparing Table 1, Entry 1 vs Table 2, Entry 4 (Figure 4).

For ST6GAL1, it appears that for the aryl‐modified uridine derivatives, an interaction between an asparagine (Asn233) and the α‐hydroxy group at the chiral centre of the **29** **a** compound (Figure 3) aided in orienting the 3‐phenoxy ring towards a collection of hydrophobic residues. This interaction seems to be more effective in the instance of the 3‐phenoxy compound, due to the increased inhibition over the other aromatic derivatives. There also appears to be a favourable π‐sulfur bond between the nucleoside ring of each docked compound and the disulphide bond between Cys353‐Cys364 that spans the active site (Figure 4). However, this bond seems to be disrupted with 5‐fluoro substitution (**31** **a**, Figure 4), which instead leads to an interaction between the fluorine and a backbone carbonyl.

When tested against ST8SIA3, the binding of the indole derivative **29** **j** (Figure 3) is aided by a considerable degree of π‐stacking between the indole ring itself and another indole ring from a tryptophan residue (Trp350). Although this residue does not appear to be interacting with the 3‐phenoxy groups of the other ligands (**29** **a**, and **29** **b** Figure 3) in these docking outputs, it is possible that in solution the Trp350 residue would also interact with other aromatic systems such as these. This interaction is supported by the stronger inhibition shown against ST8SIA2 by the heterocyclic or 3‐phenoxy derivatives in Table 1. Regarding the nucleoside component, there is little difference in binding, except for the 5*‐*fluorouridine derivative (**31** **a**), where the 5‐fluoro substituent forms a halogen bond with a carbonyl on the protein backbone (Figure 4).

Bringing together the results from the enzymatic assay and docking study, the main findings are that 5‐substituted derivatives show selectivity for ST8SIA2, while 2′‐modifications reduced affinity for ST3GAL1. On the aromatic fragment, ST6GAL1 showed a higher affinity for aliphatic substituents at the 3‐position on the aryl moiety compared to the other two enzymes, while ST8SIA2 had a higher affinity for heterocyclic moieties appended to the triazole linker.

There are a host of exciting strategies for targeting sialylation in cancer including Bertozzi's approach to desialylate cancer cells with *Vibrio cholera* sialidase conjugated to the HER2 targeting antibody Trastuzumab,^[13]^ and new opportunities on remodelling of cell‐surface glycans by extracellular STs.^[63]^ Although some STs are widely studied in cancer, such as ST3GAL1/ST6GAL1, several ST subtypes are underexplored. The metabolic pan‐ST inhibitor, peracetylated 3F_ax_‐Neu5Ac, is commercially available and widely used in cell studies on sialylation, however, its clinical use is limited by toxicity issues. To address this, Büll *et al*. have selectively delivered peracetylated 3F_ax_‐Neu5Ac encapsulated into tumour‐targeting nanoparticles to prevent lung cancer metastasis.^[64]^ Alongside these desialylation strategies that use a pan‐ST inhibitor (or sialidase) coupled with a targeting agent, the development of small molecule ST inhibitors that exhibit inherent selectivity towards specific ST subtypes is an important complementary strategy to limit off‐target effects and enable ST inhibitors to proceed to the clinic.

## Conclusions

In this work, we present the design and synthesis of a new series of α‐hydroxy‐1,2,3‐triazole linked uridyl‐based ST inhibitors that show favourable PK parameters compared to charged phosphodiester‐based ST inhibitors. The newly developed inhibitors incorporate a range of structural modifications to probe for selectivity across ST subtypes. The 5‐substituted nucleosides such as thymidine or 5‐fluorouridine gave dramatically enhanced selectivity for ST8SIA2, while aliphatic substitutions on the aryl moiety, which potentially mimics the sialic acid, are more tolerated by ST6GAL1 than the other two subtypes tested. Although selective targeting of ST3GAL1 remains elusive amongst the structural modifications investigated in this work, further computationally‐guided design is ongoing to develop ST3‐selective inhibitors in the future. This study also highlighted that while the presence of a phosphonate moiety is important for activity against ST3 and ST6, it appears less crucial for ST8, providing a new avenue to explore to develop ST8 selective inhibitors further. Combined with our previous work on phosphonate‐bearing, triazole‐linked ST inhibitors, this work provides important information for the design of subtype‐selective ST inhibitors with improved synthetic accessibility, drug‐likeness and enhanced ST sub‐type selectivity.

## Experimental Section

### General Synthetic Experimental

All commercially available starting materials, reagents and solvents were used without further purification. Thin layer chromatography (TLC) was performed on aluminium‐backed 0.20 mm silica gel plates. Visualisation was accomplished with UV light. Flash chromatography was performed under positive air pressure using Silica Gel 60 of 230–400 mesh (40–63 μm). Optical Rotations were measured at 20 °C in the specified solvent with a path length of 1.0 dm on a Jasco P‐2000 Digital Polarimeter (λ=589 nm). ${\left[\alpha \right]}_{D}^{20}$ values are given in 10^−1^ deg cm^2^ g^−1^ and are shown in the form ${\left[\alpha \right]}_{D}^{20}$ 22 (c 0.95 in MeOH), where concentrations (c) are given in g per 100 mL. Proton and carbon magnetic resonance spectra (^1^H NMR and ^13^C NMR) were recorded on a Varian Mercury 300 MHz spectrometer, a Varian Inova 500 MHz spectrometer or a Varian V NMRS PS54 500 MHz spectrometer. Low resolution mass spectrometry (MS) was performed on a Shimadzu LC‐2010 Electrospray Ionization (ESI) Mass Spectrometer. High resolution mass spectrometry (HRMS) was performed on a Waters Quadrupole‐Time of Flight (QTOF) Xevo Spectrometer via ESI with Leucine–Enkephalin as an internal standard.

Nucleoside compounds **12**,^[58]^ **13**,^[65]^ **20**,^[66]^ and **21** ^[66]^ were synthesized by known procedures. All other starting materials were commercially available. All reagents, except commercial products of satisfactory quality, were purified by literature procedures prior to use.

The synthesis of α‐arylpropargyl acetates is described in the supplementary material.

### Synthesis of Novel Nucleoside Derivatives

**3′‐O‐Acetyl‐2′, 5′‐dideoxy‐5′‐tosyluridine (18)**. A solution of 2′‐deoxyuridine (600 mg, 2.63 mmol) in dry pyridine was cooled with an external ice bath and treated with TsCl (551 mg, 2.89 mmol) and stirred at room temperature for 1 h. Ac_2_O (1.25 mL) was added to the reaction mixture and stirred for another 3 h. The solution was then taken up in CH_2_Cl_2_ and washed with 0.5 M HCl, water and brine. The organic layer was dried on MgSO_4_ and evaporated under reduced pressure and the residue was purified using silica gel chromatography eluted with (CH_2_Cl_2_/EtOAc, 2 : 1) to afford the title compound as a pale‐yellow syrup (692 mg, 62 %). ^1^H NMR (500 MHz, CDCl_3_): δ 8.14 (br s, 1H), 7.80 (d, *J*=8.3 Hz, 2H), 7.53 (d, *J*=7.8 Hz, 1H), 7.40 (d, *J*=8.3 Hz, 2H), 6.33 (dd, *J*=8.8, 5.4 Hz, 1H) 5.76 (dd, *J*=8.3, 1.9 Hz, 1H), 5.21–5.19 (m, 1H), 4.33–4.32 (m, 2H), 4.19–4.18 (m, 1H), 2.48 (s, 3H), 2.47–2.43 (m, 1H′), 2.19–2.13 (m, 1H), 2.10 (s, 3H). ^13^C NMR (125 MHz, CDCl_3_): δ 170.4, 163.3, 150.4, 145.5, 139.1, 131.9, 130.0, 127.7, 103.0, 84.7, 81.9, 74.2, 68.9, 37.0, 21.5, 20.7. HRMS (ESI) *m/z* calcd for C_18_H_20_N_2_O_8_S [M+Na]^+^: 447.0838, found 447.0839. ${\left[\alpha \right]}_{D}^{20}$ =27.8 (c 0.5 in MeOH).

**3′‐*O*‐Acetyl‐5′‐azido‐2′,5′‐dideoxy‐2′‐fluorouridine (22)**. Compound **19** (450 mg, 1.02 mmol) was dissolved in dry DMF (40 mL) and NaN_3_ (132 mg, 2.03 mmol) and benzyltriethylammonium chloride (46 mg, 203 μmol) was added. The suspension was stirred at 80 °C overnight. The reaction mixture was poured onto crushed ice and extracted with CH_2_Cl_2_. The organic layer was washed several times with water and brine, dried over MgSO_4_, filtered and evaporated and subsequently purified by flash chromatography to yield the title compound **22** as a white solid (283 mg, 88 %). m.p. 172–174 °C; ^1^H NMR (300 MHz, CDCl_3_): δ 9.25 (br s, 1H), 7.42 (d, *J*=7.9 Hz, 1H), 5.86 (dd, *J*=19.8 Hz, 2.0 Hz, 1H), 5.82 (d, *J*=7.9 Hz, 1H), 5.38 (ddd, *J*=31.5 Hz, 3.3 Hz, 3.0 Hz, 1H), 5.25–5.19 (m, 1H), 4.32–4.29 (m, 1H), 3.80 (dd, *J*=13.5 Hz, *J*=2.4 Hz, 1H), 3.60 (dd, *J*=13.5 Hz, *J*=4.4 Hz, 1H), 2.17 (s, 3H); ^13^C NMR (75 MHz, CDCl_3_): δ 169.8, 162.8, 149.8, 140.8, 103.3, 91.1 (d, *J*=60.8 Hz), 90.4 (d, *J*=83.3 Hz), 79.4, 70.2 (d, *J*=15.3 Hz), 51.0, 20.4; HRMS (ESI) *m/z* calcd for C_11_H_12_FN_5_O_5_Na [M+Na]^+^: 336.0720; found 336.0734; ${\left[\alpha \right]}_{D}^{20}$ =32.6 (c 0.5 in MeOH).

**3′‐*O*‐Acetyl‐5′‐*tert*‐butyldimethylsilyl‐2′‐*O*‐methyluridine (24)**. A solution of 2’‐*O*‐methyluridine (600 mg, 2.32 mmol) and imidazole (395 mg, 5.81 mmol) in dry DMF (5 mL) was treated with *t*‐butyldimethylsilyl chloride (385 mg, 2.55 mmol) and stirred at room temperature for 20 h. The reaction mixture was poured on crushed ice and the white precipitate was collected by filtration, air dried and used in the following step without further purification. The solid was suspended in CH_2_Cl_2_ (12 mL) and added successively trimethylamine (0.5 mL, 3.48 mmol) and Ac_2_O (0.26 mL, 2.79 mmol) and the resulting solution was stirred at room temperature. After 3 h, the starting material was no longer detectable by TLC and the solution was diluted with CH_2_Cl_2, washed_ with water, NaHCO_3_ and brine, and finally dried over MgSO_4_, filtered and evaporated under reduced pressure. The residue was purified by silica gel chromatography to give the title compound **24** as a colourless syrup (810 mg, 84 %). ^1^H NMR (300 MHz, CDCl_3_): δ 9.60 (br s, 1H), 7.92 (d, *J*=7.9 Hz, 1H), 6.06 (d, *J*=2.7 Hz, 1H), 5.70 (d, *J*=7.9 Hz, 1H), 5.14–5.12 (m, 1H), 4.22–4.20 (m, 1H), 4.00–3.97 (m, 2H), 3.79–3.77 (m, 1H), 3.43 (s, 3H), 2.14 (s, 3H), 0.92 (s, 9H), 0.10 (s, 6H); ^13^C NMR (75 MHz, CDCl_3_): δ 170.2, 163.3, 150.4, 139.7, 102.6, 86.8, 82.6, 82.5, 70.0, 62.1, 59.0, 25.9, 20.8, 18.4, −5.5, −5.7. HRMS (ESI) m/z calcd for C_18_H_30_N_2_O_7_SiNa [M+Na]^+^: 437.5198; found 437.5199; ${\left[\alpha \right]}_{D}^{20}$ =25.3 (c 0.5 in MeOH).

**3′‐*O*‐Acetyl‐5′‐azido‐5′‐deoxy‐2′‐*O*‐methyluridine (27)**. A suspension of **26** (320 mg, 0.85 mmol) and NaN_3_ (76.0 mg, 1.27 mmol) in DMF (5 mL) was stirred at 90 °C for 6 h. The mixture was then poured on crushed ice and the solution was extracted with CH_2_Cl_2_ (3×15 mL). The organic layer was washed with water and brine, dried over MgSO_4_, evaporated under reduced pressure and by silica gel chromatography eluted with (CH_2_Cl_2_:EtOAc, 3 : 1) to afford a white foam (250 mg, 91 %). ^1^H NMR (500 MHz, CDCl_3_): δ 7.56 (d, *J*=7.8 Hz, 1H), 5.98 (d, *J*=3.4 Hz, 1H), 5.83 (d, *J*=7.8 Hz, 1H), 5.09–5.07 (m, 1H), 4.25 (br s, 1H), 4.04–4.03 (m, 1H), 3.81 (d, *J*=13.7 Hz, 1H), 3.66 (d, *J*=13.2 Hz, 1H) 3.44 (s, 3H), 2.17 (s, 3H). ^13^C NMR (125 MHz, CDCl_3_): δ 170.1, 162.7, 150.1, 139.5, 103.3, 88.0, 81.3, 80.0, 70.6, 59.1, 51.5, 20.6. HRMS (ESI) m/z calcd for C_12_H_15_N_5_O_6_ [M+H]+: 348.0920, found 348.0917. ${\left[\alpha \right]}_{D}^{20}$ =37.9 (c 0.5 in MeOH).

### Synthesis of Acetyl‐Protected Triazole‐Based Inhibitors

Protected 5′‐azidonucleoside (1 equiv.), alkyne derivative (1.2 equiv.), copper acetate (0.2 equiv.) and sodium ascorbate (0.5 equiv.) were suspended in a mixture of water and acetonitrile (1 : 2). After disappearance of the starting material, as indicated by TLC, the reaction mixture was concentrated under reduced pressure and extracted with ethyl acetate. The organic phase was then washed with brine, dried over magnesium sulphate and evaporated to dryness. The residue was purified by flash chromatography and eluted using CH_2_Cl_2_ : EtOAc (1 : 2) to EtOAc:Acetone (4 : 1).

**5′‐(4′′‐(α‐Acetoxy‐3′′′‐phenoxybenzyl)triazolo)‐2′, 3′‐*O*‐diacetyl‐5′‐deoxyuridine (28** **a)**. From the acetyl‐protected azidouridine **12** (120 mg, 340 μmol), the title compound was obtained as a pale yellow oil (189 mg, 90 %). ^1^H NMR (300 MHz, CDCl_3_): δ 9.63 (br s, 1H), 7.50 (2×s, 1H), 7.47–7.44 (m, 1H), 7.36–7.24 (m, 2H), 7.21–7.03 (m, 3H), 7.02‐6,95 (m, 3H), 6.90 (m, 1H), 5.72–5.69 (m, 1H), 5.64–5.62 (m, 1H), 5.37–5.30 (m, 1H), 5.30–5.26 (m, 1H), 4.77–4.75 (m, 1H), 4.76–4.65 (m, 2H), 4.47–4.42 (m, 1H), 2.14 (2×s, 3H), 2.08 (2×s, 6H); ^13^C NMR (75 MHz, CDCl_3_): δ 169.8, 169.7, 169.5, 162.7, 158.2, 157.4, 150.0, 146.7, 146.6, 141.2, 139.0, 129.7, 129.6, 123.4, 121.7, 118.9, 118.8, 118.2, 117.3, 103.0, 102.9, 91.3, 91.2, 79.9, 72.8, 70.5, 69.8, 51.0, 21.2, 21.1, 20.6, 20.5. HRMS (ESI) *m/z* calcd for C_30_H_29_N_5_O_10_Na [M+Na]^+^: 642.1812; found 642.1837. ${\left[\alpha \right]}_{D}^{20}$ =11.6 (c 0.5 in MeOH).

**5′‐(4′′‐(α‐Acetoxy‐4′′′‐fluoro‐3′′′‐phenoxybenzyl)triazolo)‐2′, 3′‐O‐diacetyl‐5′‐deoxy‐uridine (28** **b)**. From the acetyl‐protected azidouridine **12** (200 mg, 566 μmol), the title compound was obtained as a dark yellow oil (314 mg, 87 %). ^1^H NMR (400 MHz, CDCl_3_): δ 9.87 (br s, 1H), 7.60 (2×s, 1H), 7.32–7.29 (m, 2H), 7.24–7.14 (m, 3H), 7.10–7.04 (m, 2H), 6.96–6.94 (m, 2H), 6.92–6.90 (m, 1H), 5.75–5.71 (m, 1H), 5.60–5.59 (m, 1H), 5.44–5.35 (m, 2H), 4.76–4.66 (m, 2H), 4.47–4.43 (m, 1H), 2.24 (s, 3H), 2.09 (s, 3H), 2.08 (s, 3H); ^13^C NMR (125 MHz, CDCl_3_): δ 169.9, 169.7, 169.6, 163.1, 156.98, 156.96, 154.0 (d, *J*=250.3 Hz), 150.1, 146.8, 146.7, 143.5, 143.4 (d, *J*=11.6 Hz), 141.8, 135.0, 134.9 (d, *J*=3.7 Hz), 129.7, 124.1 124.0, 123.67 (2×d, *J*=7.5 Hz), 123.21, 123.19, 120.90, 120.85, 117.16 (2×d, *J*=8.0 Hz), 117.03, 103.24, 103.18, 91.96, 91.95, 79.98, 79.95, 72.83, 72.81, 70.6, 70.4, 69.02, 68.95, 51.11, 51.07, 21.02, 21.00, 20.33, 20.26. HRMS (ESI) *m/z* calcd for C_30_H_28_FN_5_O_10_Na [M+Na]^+^: 660.1718; found 660.1713; ${\left[\alpha \right]}_{D}^{20}$ =14.9 (c 0.5 in MeOH).

**5′‐(4′′‐(α‐Acetoxy‐3′′′‐cyclopentoxybenzyl)triazolo)‐2′, 3′‐O‐diacetyl‐5′‐deoxyuridine (28** **c)**. From the acetyl‐protected azidouridine **12** (220 mg, 623 μmol), the title compound was obtained as a dark yellow syrup (339 mg, 89 %). ^1^H NMR (300 MHz, CDCl_3_): δ 8.56 (2×br s, 1H), 7.48 (2×s, 1H), 7.27–7.247 (m, 1H), 7.00–6.95 (m, 3H), 6.93 (d, *J*=8.0 Hz, 1H), 6.84–6.82 (m, 1H), 5.72–5.69 (m, 1H), 5.63–5.61 (m, 1H), 5.41–5.33 (m, 1H), 5.29–5.25 (m, 1H), 4.78–4.74 (m, 1H), 4.72–4.65 (m, 2H), 4.45–4.41 (m, 1H), 2.15 (2×s, 3H), 2.09 (s, 6H), 1.89–1.75 (m, 8H); ^13^C NMR (75 MHz, CDCl_3_): δ 169.8, 169.6, 161.8, 158.3, 149.7, 147.6, 141.3, 141.2, 139.6, 129.7, 124.1, 118.82, 118.79, 115.2, 115.1, 114.7, 103.4, 91.3, 79.9, 79.4, 72.7, 70.6, 69.9, 69.8, 50.9, 32.8, 24.0, 21.2, 20.39, 20.35. HRMS (ESI) *m/z* calcd for C_29_H_33_N_5_O_10_Na [M+H]^+^: 634.2125; found 634.2142; ${\left[\alpha \right]}_{D}^{20}$ =37.4 (c 0.5 in MeOH).

**5′‐(4′′‐(α‐Acetoxy‐3′′′‐cyclopentoxy‐4′′′‐methoxybenzyl)triazolo)‐2′, 3′‐O‐diacetyl‐5′‐deoxyuridine (28** **d)**. From the acetyl‐protected azidouridine **12** (250 mg, 708 μmol), the title compound was obtained as a dark yellow oil (381 mg, 84 %). ^1^H NMR (500 MHz, CDCl_3_): δ 9.67 (br s, 1H), 7.51 (2×s, 1H), 7.06–7.04 (m, 1H), 7.03–6.95 (m, 2H), 6.92–6.89 (m, 1H), 6.83–6.81 (m, 1H), 5.74–5.68 (m, 1H), 5.64–5.63 (m, 1H), 5.42–5.35 (m, 1H), 5.31–5.27 (m, 1H), 4.78–4.74 (m, 1H), 4.72–4.66 (m, 2H), 4.46–4.41 (m, 1H), 3.81 (s, 3H), 2.12 (2×s, 3H), 2.07–2.06 (m, 6H), 1.90–1.77 (m, 6H), 1.59–1.56 (m, 2H); ^13^C NMR (125 MHz, CDCl_3_): δ 170.0, 169.9, 169.60, 169.59, 169.55, 169.54, 162.94, 162.92, 157.1, 150.2, 150.0, 147.61, 147.58, 147.55, 141.4, 130.5, 130.4, 124.0, 123.9, 119.71, 119.66, 114.34, 114.30, 111.69, 111.66, 103.32, 103.27, 91.3, 91.2, 80.48, 80.47, 80.0, 79.9, 72.74, 72.66, 70.6, 70.4, 69.83, 69.80, 55.98, 55.97, 60.0, 59.9, 32.7, 32.6, 23.94, 23.92, 21.1, 20.3, 20.26, 20.24; HRMS (ESI) *m/z* calcd for C_30_H_35_N_5_O_11_Na [M+Na]^+^: 664.2231; found 664.2246; ${\left[\alpha \right]}_{D}^{20}$ =15.4 (c 0.5 in MeOH).

**5′‐(4′′‐(α‐Acetoxy‐3′′′‐propoxybenzyl)triazolo)‐2′, 3′‐O‐diacetyl‐5′‐deoxyuridine (28** **e)**. From the acetyl‐protected azidouridine **12** (173 mg, 490 μmol), the title compound was obtained as a dark yellow oil (258 mg, 90 %). ^1^H NMR (500 MHz, CDCl_3_): δ 9.18 (br s, 1H), 7.52 (2×s, 1H), 7.28–7.25 (m, 1H), 7.03–6.94 (m, 4H), 6.86–6.84 (m, 1H), 5.72–5.69 (m, 1H), 5.63–5.62 (m, 1H), 5.43–5.35 (m, 1H), 5.32–5.27 (m, 1H), 4.75–4.67 (m, 2H), 4.46–4.41 (m, 1H), 3.93–3.90 (m, 2H), 2.15 (2×s, 3H), 2.09 (2×s, 6H), 1.79 (sxt, *J*=7.5 Hz, 2H), 1.02 (t, *J*=7.5 Hz, 3H); ^13^C NMR (125 MHz, CDCl_3_): δ 169.9, 169.8, 169.6, 169.56, 169.55, 162.59, 162.57, 159.31, 159.29, 149.90, 149.87, 147.5, 141.3, 139.66, 139.65, 129.68, 129.67, 124.1, 124.0, 119.10, 119.07, 114.3, 114.2, 113.6, 103.4, 103.3, 91.4, 91.3, 80.0, 79.9, 72.72, 72.70, 70.6, 70.4, 69.87, 69.86, 69.6, 50.92, 50.86, 22.5, 21.1, 20.34, 20.29, 10.5. HRMS (ESI) *m/z* calcd for C_27_H_31_N_5_O_10_Na [M+Na]^+^: 608.1969; found 608.2043; ${\left[\alpha \right]}_{D}^{20}$ =12.6 (c 0.5 in MeOH).

**5′‐(4′′‐(α‐Acetoxy‐benzothien‐2′′′‐ylmethyl)triazolo)‐2′, 3′‐O‐diacetyl‐5′‐deoxyuridine (28** **f)**. From the acetyl‐protected azidouridine **12** (300 mg, 849 μmol), the title compound was obtained as a dark yellow oil (456 mg, 92 %). ^1^H NMR (500 MHz, CDCl_3_): δ 9.25–9.23 (m, 1H), 7.79 (2×s, 1H), 7.74–7.72 (m, 2H), 7.38–7.31 (m, 4H), 7.03 (2×d, *J*=8.0 Hz, 1H), 5.71–5.69 (m, 1H), 5.63–5.60 (m, 1H), 5.45–5.38 (m, 1H), 5.33–5.31 (m, 1H), 4.80–4.70 (m, 2H), 4.49–4.45 (m, 1H), 2.16 + 2.15 (2×s, 3H), 2.09 (s, 6H). ^13^C NMR (125 MHz, CDCl_3_): δ 169.8, 169.7, 169.6, 162.69, 162.67, 150.0, 149.9, 146.29, 146.26, 141.54, 141.51, 141.22, 141.17, 139.9, 139.8, 138.9, 124.9, 124.6, 124.31, 124.26, 124.0, 123.78, 123.76, 122.3, 103.37, 103.35, 91.6, 80.1, 80.0, 72.8, 72.7, 70.6, 70.5, 66.29, 66.25, 51.2, 51.1, 21.1, 21.0, 20.4, 20.34, 20.30; HRMS (ESI) *m/z* calcd for C_26_H_25_N_5_O_9_SNa [M+Na]^+^: 606.1271; found 606.1276; ${\left[\alpha \right]}_{D}^{20}$ =32.1 (c 0.5 in MeOH).

**5′‐(4′′‐(α‐Acetoxy‐N‐tert‐butoxycarbonylindol‐3′′′‐ylmethyl)triazolo)‐2′, 3′‐O‐diacetyl‐5′‐deoxyuridine (28** **g)**. From the acetyl‐protected azidouridine **12** (200 mg, 566 μmol), the title compound was obtained as a pale yellow oil (340 mg, 90 %). ^1^H NMR (500 MHz, CDCl_3_): δ 8.92 (br s, 1H), 8.13–8.12 (m, 1H), 7.75 (2×s, 1H), 7.66–7.65 (m, 1H), 7.56–7.53 (m, 1H), 7.34–7.30 (m, 2H), 7.22–7.19 (m, 1H), 7.03‐6.99 (m, 1H), 5.73–5.69 (m, 1H), 5.58–5.57 (m, 1H), 5.44–5.30 (m, 2H), 4.79–4.76 (m, 1H), 4.70–4.64 (m, 1H), 4.46–4.42 (m, 1H), 2.14 (2×s, 3H), 2.10 (s, 6H), 1.67 (s, 9H); ^13^C NMR (125 MHz, CDCl_3_): δ 170.2, 170.0, 169.7, 162.61, 162.57, 149.73, 149.72, 146.4, 146.3, 141.6, 128.2, 125.22, 125.17, 124.8, 124.3, 124.1, 122.83, 122.81, 119.8, 115.5, 103.34, 103.28, 91.9, 84.4, 80.0, 79.9, 72.9, 72.8, 70.5, 70.2, 63.84, 63.85, 51.0, 50.8, 28.1, 21.11, 21.09, 20.40, 20.35; HRMS (ESI) m/z calcd for C_31_H_34_N_6_O_11_Na [M+Na]^+^: 689.2183; found 689.2198; ${\left[\alpha \right]}_{D}^{20}$ =23.8 (c 0.5 in MeOH).

**5′‐(4′′‐(N‐tert‐butoxycarbonylindol‐3′′′‐yl)‐α‐hydroxy‐α‐methoxycarbonylmethyl)triazolo)‐2′, 3′‐O‐diacetyl‐5′‐deoxyuridine (28** **h)**. From the acetyl‐protected azidouridine **12** (200 mg, 566 μmol), the title compound was obtained as a dark yellow oil (313 mg, 81 %). ^1^H NMR (300 MHz, CDCl_3_): δ 8.72 (br s, 1H), 8.15–8.13 (m, 1H), 7.77–7.73 (m, 1H), 7.52–7.47 (m, 1H), 7.33–7.29 (m, 2H), 7.19–7.13 (m, 1H), 7.01‐6.92 (m, 1H), 5.75–5.69 (m, 1H), 5.60–5.55 (m, 1H), 5.40–5.39 (m, 1H), 5.32–5.25 (m, 1H), 4.80–4.58 (m, 3H), 4.43 (br s, 1H), 3.86 (2×s, 3H), 2.12 (s, 3H), 2.10 (s, 3H), 1.67 (s, 9H); ^13^C NMR (75 MHz, CDCl_3_): δ 169.63, 169.57, 169.58, 162.3, 153.6, 149.7, 149.6, 143.9, 142.0, 141.6, 141.4, 136.0, 127.9, 125.6, 125.2, 124.6, 124.5, 122.8, 122.7, 121.1, 115.3, 103.36, 103.44, 91.8, 84.41, 84.38, 82.1, 82.0, 79.92, 79.90, 72.8, 70.4, 53.9, 50.93, 50.88, 28.2, 20.38, 20.36; HRMS (ESI) *m/z* calcd for C_31_H_34_N6O_12_Na [M+Na]^+^: 705.2132; found 705.2140; ${\left[\alpha \right]}_{D}^{20}$ =33.4 (c 0.5 in MeOH).

**5′‐(4′′‐(α‐Acetoxy‐α‐trifluoromethylbenzyl)triazolo)‐2′,3′‐O‐diacetyl‐5′‐deoxyuridine (28** **i)**. From the acetyl‐protected azidouridine **12** (200 mg, 566 μmol), the title compound was obtained as a pale yellow oil (327 mg, 97 %). ^1^H NMR (500 MHz, CDCl_3_): δ 9.39–9.34 (m, 1H), 7.84–7.82 (m, 1H), 7.50–7.46 (m, 2H), 7.43–7.39 (m, 3H), 7.04–6.95 (m, 1H), 5.81–5.72 (m, 1H), 5.67–5.44 (m, 1H), 5.42–5.09 (m, 2H), 4.88–4.72 (2×m, 2H), 4.50–4.43 (m, 1H), 2.23 (2×s, 3H), 2.10–2.07 (m, 3H); ^13^C NMR (125 MHz, CDCl_3_): δ 169.7, 169.6, 169.6, 169.4, 168.0, 167.7, 162.8, 162.7, 150.3, 150.2, 142.9, 142.6, 141.2, 140.8, 133.1, 132.7, 129.5, 129.3, 128.3, 128.2, 127.5–127.3 (m), 127.4, 127.3, 103.5, 103.4, 90.4, 89.3, 80.0, 79.7, 72.4, 72.0, 70.5, 70.0, 50.9, 50.6, 21.7, 20.3; HRESI‐MS *m/z* calcd for C_25_H_24_N_5_O_9_Na [M+Na]^+^: 618.1424; found 618.1437; ${\left[\alpha \right]}_{D}^{20}$ =29.5 (c 0.5 in MeOH).

**5′‐(4′′‐(α‐Acetoxy‐3′′′‐phenoxybenzyl)triazolo)‐2′, 3′‐*O*‐acetyl‐5′‐deoxy‐5‐fluorouridine (30** **a)**. From the acetyl‐protected 5‐fluoroazidouridine **13** (220 mg, 593 μmol), the title compound was obtained as a dark yellow oil (363 mg, 96 %); ^1^H NMR (500 MHz, CDCl_3_): δ 9.89 (br s, 1H), 7.59 (2×s, 1H), 7.32–7.28 (m, 3H), 7.23–7.22 (m, 1H), 7.20–7.17 (m, 1H), 7.14–7.08 (m, 2H), 7.00–6.97 (m, 3H), 6.91–6.89 (m, 1H), 5.66–5.62 (m, 1H), 5.41–5.37 (m, 1H), 5.35–5.29 (m, 1H), 4.78–4.67 (m, 2H), 4.48–4.44 (m, 1H), 2.11 (s, 3H), 2.08 (s, 3H), 2.07 (s, 3H); ^13^C NMR (125 MHz, CDCl_3_): δ 170.0, 169.9, 169.78, 169.76, 169.7, 157.40, 157.37, 156.7 (2×d, *J*=26.8 Hz), 156.6, 148.84, 148.79, 147.2, 147.1, 140.6 (d, *J*=239.4 Hz), 140.11, 140.06, 129.94, 129.92, 129.7, 125.6 (2×d, *J*=34.5 Hz), 124.2, 124.1, 123.5, 121.74, 121.72, 118.9, 118.27, 118.25, 117.4, 117.3, 91.2, 90.9, 79.9, 72.7, 72.6, 70.4, 70.3, 69.5, 69.4, 50.9, 21.03, 20.98, 20.31, 20.27; HRMS (ESI) *m/z* calcd for C_30_H_28_FN_5_O_10_Na [M+Na]^+^: 660.1718; found 660.1733; ${\left[\alpha \right]}_{D}^{20}$ =38.5 (c 0.5 in MeOH).

**5′‐(4′′‐(α‐Acetoxy‐3′′′‐phenoxybenzyl)triazolo)‐3′‐*O*‐acetyl‐5′‐dideoxythymidine (30** **b)**. From the acetyl‐protected azidothymidine **20** (200 mg, 647 μmol), the title compound was obtained as a dark yellow oil (350 mg, 94 %). ^1^H NMR (500 MHz, CDCl_3_): δ 8.38. (br s, 1H), 7.51 (d, *J*=9.8 Hz, 1H), 7.35–7.30 (m, 3H), 7.18–7.17 (m, 1H), 7.12–7.10 (m, 2H), 7.01–6.92 (m, 4H), 6.88–6.87 (m, 1H), 6.18–6.14 (m, 1H), 5.29–5.21 (m, 1H), 4.73–4.72 (m, 2H), 4.30–4.27 (m, 1H), 2.36–2.32 (m, 1H), 2.17–2.14 (m, 1H), 2.12 (s, 3H), 2.10 (s, 3H), 1.89 (d, *J*=0.9 Hz, 3H); ^13^C NMR (125 MHz, CDCl_3_): δ 170.7, 169.7, 163.0, 157.6, 156.7, 150.0, 147.3, 140.14, 140.05, 135.5, 135.4, 130.03, 130.01, 129.8, 124.1, 124.0, 123.6, 121.8, 119.1, 118.5, 117.4, 117.3, 111.9, 85.4, 82.1, 74.4, 74.2, 69.6, 51.4, 51.3, 36.1, 21.10, 21.07, 20.8, 12.4; HRMS (ESI) *m/z* calcd for C_29_H_29_N_5_O_8_Na [M+Na]^+^: 598.1914; found 598.1917; ${\left[\alpha \right]}_{D}^{20}$ =48.3 (c 0.5 in MeOH).

**5′‐(4′′‐(α‐Acetoxy‐3′′′‐phenoxybenzyl)triazolo)‐3′‐*O*‐acetyl‐2′, 5′‐dideoxyuridine (30** **c)**. From the acetyl‐protected 2′‐deoxyazidouridine **21** (245 mg, 830 μmol), the title compound was obtained as a dark yellow oil (394 mg, 85 %); ^1^H NMR (500 MHz, CDCl_3_): δ 9.88 (br s, 1H), 7.56–7.52 (m, 1H), 7.33–7.29 (m, 3H), 7.19–7.16 (m, 1H), 7.12–7.08 (m, 2H), 7.06–7.04 (m, 1H), 7.00–6.91 (m, 4H), 6.17–6.16 (m, 1H), 5.70–5.67 (m, 1H), 5.31–5.25 (m, 1H), 4.80–4.75 (m, 1H), 4.70–4.66 (m, 1H), 4.28 (br s, 1H), 2.36–2.30 (m, 1H), 2.12 (s, 3H), 2.09 (s, 3H), 2.06–2.02 (m, 1H); ^13^C NMR (125 MHz, CDCl_3_): δ 170.5, 169.6, 163.1, 163.0, 157.50, 157.47, 156.54, 156.52, 150.30, 150.27, 147.03, 146.96, 140.0, 139.8, 139.7, 130.0, 129.9, 129.7, 124.35, 124.15, 123.50, 123.47, 121.60, 121.55, 119.0, 118.27, 118.25, 117.17, 117.2, 103.3, 103.2, 85.3, 85.2, 82.1, 82.0, 74.3, 74.1, 69.53, 69.46, 51.1, 36.08, 36.05, 21.0, 20.6; HRMS (ESI) *m/z* calcd for C_28_H_27_N_5_O_8_Na [M+Na]^+^: 584.1757; found 584.1749; ${\left[\alpha \right]}_{D}^{20}$ =51.2 (c 0.5 in MeOH).

**5′‐(4′′‐(α‐Acetoxy‐3′′′‐phenoxybenzyl)triazolo)‐3′‐*O*‐acetyl‐2′, 5′‐dideoxy‐2′‐fluorouridine (30** **d)**. From the acetyl‐protected 2′‐deoxy‐2′‐fluoroazidouridine **22** (220 mg, 702 μmol), the title compound was obtained as a pale yellow oil (382 mg, 94 %). ^1^H NMR (300 MHz, CDCl_3_): δ 9.68 (br s, 1H); 7.56–7.55 (m, 1H), 7.34–7.28 (m, 3H), 7.20–7.17 (m, 1H), 7.14–7.12 (m, 1H), 7.10–7.08 (m, 1H), 7.06–7.02 (m, 1H), 7.01–7.00 (m, 1H), 6.99–6.98 (m, 1H), 6.97 (s, 1H), 6.92–6.89 (m, 1H), 5.72–5.69 (m, 1H), 5.59–5.51 (m, 1H), 5.40–5.37 (m, 1H), 5.33–5.22 (m, 1H), 4.77–4.71 (m, 1H), 4.67–4.62 (m, 1H), 4.52–4.46 (m, 1H), 2.12 (2×s, 3H), 2.10 (2×s, 3H); ^13^C NMR (75 MHz, CDCl_3_): δ 169.9, 169.8, 169.7, 162.9, 157.44, 157.43, 156.7, 149.80, 149.76, 147.1, 147.0, 142.43, 142.36, 140.21, 140.15, 129.92, 129.90, 129.8, 124.0, 123.9, 123.5, 121.81, 121.75, 118.98, 118.96, 118.31, 118.27, 117.42, 117.40, 103.3, 103.2, 93.9 (2×d, *J*=30.0 Hz), 90.69 (2×d, *J*=190.5 Hz), 79.01, 78.98, 71.1 (2×d, *J*=15.5 Hz), 69.6, 69.5, 50.9, 50.8, 21.03, 21.00, 20.2; HRMS (ESI) *m/z* calcd for C_28_H_26_FN_5_O_8_Na [M+Na]^+^: 602.1663; found 602.1669; ${\left[\alpha \right]}_{D}^{20}$ =69.3 (c 0.5 in MeOH).

**5′‐(4′′‐(α‐Acetoxy‐3′′′‐phenoxybenzyl)triazolo)‐3′‐O‐acetyl‐5′‐deoxy‐2′‐*O*‐methyluridine (30** **e)**. From the acetyl‐protected 2′‐methoxyoxyazidouridine **27** (160 mg, 492 μmol), the title compound was obtained as a dark yellow oil (241 mg, 83 %). ^1^H NMR (300 MHz, CDCl_3_): δ 9.21 (br s, 1H), 7.53–7.49 (m, 1H), 7.35–7.30 (m, 3H), 7.20–7.17 (m, 1H), 7.13–7.10 (m, 2H), 7.02–6.97 (m, 2H), 6.94–6.92 (m, 2H), 6.83 (2×d, *J*=8.3 Hz, 1H), 5.74–5.72 (m, 1H), 5.70–5.66 (m, 1H), 5.14–5.08 (m, 1H), 4.70–4.69 (m, 2H), 4.45–4.41 (m, 1H), 3.98–3.93 (m, 1H), 3.37 (2×s, 3H), 2.14–2.13, (m, 6H); ^13^C NMR (75 MHz, CDCl_3_): δ 170.05, 170.04, 169.9, 169.8, 162.7, 162.6, 157.60, 157.55, 156.64, 156.62, 150.0, 149.9, 147.3, 147.2, 140.5, 140.3, 140.05, 140.03, 130.04, 130.01, 129.8, 124.4, 124.2, 123.59, 123.57, 121.70, 121.65, 119.08, 119.06, 118.39, 118.37, 117.34, 117.28, 103.4, 103.3, 90.31, 90.25, 80.2, 80.1, 79.5, 71.1, 71.0, 69.60, 69.55, 59.1, 50.74, 50.70, 21.1, 20.5. HRMS (ESI) *m/z* calcd for C_29_H_29_N_5_O_9_Na [M+Na]^+^: 614.1863; found 614.1858; ${\left[\alpha \right]}_{D}^{20}$ =54.7 (c 0.5 in MeOH).

### Deprotection of Acetyl‐Protected Inhibitors.

The protected 5’‐triazolonucleoside derivative was taken up in MeOH (2–5 mL) and concentrated ammonia (2–3 mL) was added to this solution. Stirring was continued until the disappearance of the starting material was observed by TLC. The solvent was evaporated under reduced pressure and coevaporated twice with MeOH to ensure the complete removal of water. The resulting residue was purified by flash chromatography.

**5′‐Deoxy‐5′‐(4′′‐(α‐hydroxy‐3′′′‐phenoxybenzyl)triazolo)uridine (29** **a)**. From the acetyl‐protected 1,2,3‐triazole **28** **a** (130 mg, 210 μmol), the title compound was obtained as a white powder (96 mg, 93 %). m.p. 173–174 °C; ^1^H NMR (500 MHz, DMSO‐d6): 11.40 (br s, 1H), 7.86–7.85 (m, 1H), 7.50–7.43 (m, 1H), 7.39–7.31 (m, 2H), 7.16–7.09 (m, 2H), 7.01‐6.99 (m, 1H), 6.88–6.86 (m, 1H), 6.09 (s, 1H), 5.82–5.81 (m, 1H), 5.76–5.75 (m, 1H), 5.61–5.55 (m, 2H), 5.43–5.42 (m, 1H), 4.71–4.66 (m, 1H), 4.64–4.60 (m, 1H), 4.16–4.13 (m, 1H), 4.05‐3.97 (m, 2H); ^13^C NMR (125 MHz, DMSO‐d6): δ 163.09, 156.66, 156.6, 151.3, 150.8, 146.4, 141.0, 130.1, 129.8, 123.6, 123.1, 121.5, 118.8, 117.2, 116.4, 102.2, 88.6, 81.9, 72.2, 70.6, 67.5, 51.2; HRMS (ESI) *m/z* calcd for C_24_H_23_N_5_O_7_Na [M+Na]^+^: 598.1944; found 598.1931; ${\left[\alpha \right]}_{D}^{20}$ =27.6 (c 0.5 in MeOH).

**5′‐Deoxy‐5′‐(4′′‐(α‐hydroxy‐4′′′‐fluoro‐3′′′‐phenoxybenzyl)triazolo)uridine (29** **b)**. From the acetyl‐protected 1,2,3‐triazole **28** **b** (180 mg, 282 μmol), the title compound was obtained as a white powder (128 mg, 89 %). m.p. 202–204 °C; ^1^H NMR (500 MHz, DMSO‐d6): δ 11.32 (s, 1H), 7.84 (s, 1H), 7.45 (dd, *J*=23.8, 8.1 Hz, 1H), 7.39–7.30 (m, 3H), 7.23–7.19 (m, 2H), 7.11 (td, *J*=7.3, 1.0 Hz, 1H), 6.96 (d, *J*=7.8 Hz), 6.11 (br s, 1H), 5.80 (br s, 1H), 5.74 (d, 5.4 Hz, 1H), 5.59 (dd, *J*=8.1, 3.9 Hz, 1H), 4.70–4.56 (m, 2H), 4.15–4.10 (m, 1H), 4.05–3.94 (m, 2H); ^13^C NMR (125 MHz, DMSO‐d6): δ 163.0, 156.8, 152.6 (d, *J*=245.6 Hz), 151.0 (d, *J*=3.4 Hz), 150.7, 142.4 (d, *J*=11.7 Hz), 141.5 (d, *J*=3.4 Hz), 141.0 (2×d, *J*=5.6 Hz), 130.1 (2×s), 123.4, 123.3, 123.0, 119.8 (2×d, *J*=4.8 Hz), 117.0, 116.9, 116.7, 102.2, 102.1, 88.7, 81.7, 72.2, 70.7, 66.9, 51.2; HRMS (ESI) *m/z* calcd for C_24_H_22_FN_5_O_7_Na [M+Na]^+^: 534.1401; found 534.1427; ${\left[\alpha \right]}_{D}^{20}$ =12.6 (c 0.5 in MeOH).

**5′‐Deoxy‐5′‐(4′‐(α‐hydroxy‐3‐cyclopentoxybenzyl)triazolo)uridine (29** **c)**. From the acetyl‐protected 1,2,3‐triazole **28** **c** (280 mg, 458 μmol), the title compound was obtained as a colourless syrup (204 mg, 92 %). ^1^H NMR (400 MHz, DMSO‐d6): δ 11.36 (br s, 1H), 7.82 (2×s, 1H), 7.45 (2×d, *J*=8.1 Hz, 1H), 7.21–7.17 (m, 1H), 6.92–6.90 (m, 2H), 6.77–6.74 (m, 1H), 5.95 (d, J=4.4 Hz, 1H), 5.77–5.73 (m, 2H), 5.60–5.57 (m, *J*=8.2 Hz, 2.1 Hz, 1H), 5.51–5.49 (m, 1H), 5.38 (d, *J*=5.1 Hz, 1H), 4.76–4.57 (m, 3H), 4.15–4.11 (m, 1H), 4.03–3.93 (m, 2H), 1.90–1.82 (m, 2H), 1.70–1.53 (m, 6H); ^13^C NMR (125 MHz, DMSO‐d6): δ 163.0, 157.5, 151.5, 150.7, 145.6, 140.9, 129.1, 122.9, 118.3, 113.8, 113.5, 102.1, 88.5, 81.8, 79.2, 72.1, 70.5, 67.8, 51.1, 32.3, 23.6; HRMS (ESI) *m/z* calcd for C_23_H_27_N_5_O_7_Na [M+Na]^+^: 508.1808; found 508.1804; ${\left[\alpha \right]}_{D}^{20}$ =21.4 (c 0.5 in MeOH).

**5′‐Deoxy‐5′‐(4′‐(α‐hydroxy‐4′′′‐methoxy‐3′′′‐cyclopentoxy‐benzyl)triazolo)uridine (29** **d)**. From the acetyl‐protected 1,2,3‐triazole **28** **d** (230 mg, 358 μmol), the title compound was obtained as a beige powder (166 mg, 90 %). m.p. 236–238 °C; ^1^H NMR (500 MHz, DMSO‐d6): δ 11.46 (br s, 1H), 7.91 (m, 1H), 7.63–7.59 (m, J=8.3 Hz, 1H), 6.94–6.93 (m, 1H), 6.89–6.87 (m, 2H), 5.85–5.83 (m, 1H), 5.66–5.62 (m, 1H), 5.47–5.40 (m, 1H), 5.37–5.35 (m, 1H), 4.75–4.70 (m, 3H), 4.45–4.40 (m, 1H), 3.72 (2×s, 3H), 1.87–1.77 (m, 2H), 1.85–1.71 (m, 4H), 1.59–1.52 (m, 2H); ^13^C R (125 MHz, DMSO‐d6): δ 162.9, 150.2, 149.2, 148.7, 146.9, 141.8, 132.6, 123.4, 119.5, 113.6, 112.0, 102.4, 88.4, 79.5, 77.3, 71.6, 70.4, 55.6, 50.5, 32.2, 23.5; HRMS (ESI) m/z calcd for C_24_H_29_N_5_O_8_Na [M+Na]^+^: 538.1914, found 538.1921; ${\left[\alpha \right]}_{D}^{20}$ =15.1 (c 0.5 in MeOH).

**5′‐Deoxy‐5′‐(4′′‐(α‐hydroxy‐3′′′‐propoxybenzyl)triazolo)uridine (29** **e)**. From the acetyl‐protected 1,2,3‐triazole **28** **e** (250 mg, 427 μmol), the title compound was obtained as a colourless syrup (184 mg, 94 %). ^1^H NMR (500 MHz, DMSO‐d6): δ 11.36 (br s, 1H), 7.83 (s, 1H), 7.45 (2×d, *J*=8.1 Hz, 1H), 7.22–7.18 (m, 1H), 6.96 (br s, 1H), 6.94–6.93 (m, 1H), 6.80–6.78 (m, 1H), 5.97–5.96 (m, 1H), 5.77–5.76 (m, 1H), 5.74–5.73 (m, 1H), 5.58 (2×d, *J*=8.1 Hz, 1H), 5.51–5.49 (m, 1H), 5.38–5.37 (m, 1H), 4.69–4.65 (m, 1H), 4.63–4.57 (m, 1H), 4.15–4.08 (m, 1H), 4.03–3.94 (m, 2H), 3.90–3.86 (m, 2H), 1.74–1.67 (m, 2H), 0.96 (2×t, *J*=7.3 Hz, 3H); ^13^C NMR (75 MHz, DMSO‐d6): δ 162.92, 158.5, 150.6, 145.63, 145.58, 140.7 + 141.0, 129.2, 129.0, 123.02, 122.98, 118.5, 118.4, 113.0, 112.9, 112.4, 112.3, 102.12, 102.07, 88.5, 88.3, 81.8, 81.7, 72.2, 72.1, 72.0, 71.9, 70.6, 70.4, 67.8, 67.7, 51.2, 51.0, 22.0, 10.5, 10.3; HRMS (ESI) *m/z* calcd for C_21_H_25_N_5_O_7_Na [M+Na]^+^: 482.1652; found 482.1667; ${\left[\alpha \right]}_{D}^{20}$ =17.9 (c 0.5 in MeOH).

**5′‐Deoxy‐5′‐(4′′‐(α‐hydroxy‐2′′′‐benzothienylmethyl)triazolo)uridine (29** **f)**. From the acetyl‐protected 1,2,3‐triazole **28** **f** (135 mg, 0.231 μmol), the title compound was obtained as a white powder (99 mg, 94 %). m.p. 202–204 °C; ^1^H NMR (500 MHz, DMSO‐d6): δ 11.37 (br s, 1H), 8.00 (2×s, 1H), 7.90–7.88 (m, 1H), 7.77–7.75 (m, 1H), 7.55–7.48 (m, 1H), 7.35–7.26 (m, 3H), 6.55 (br s, 1H), 6.16 (br s, 1H), 5.75–5.74 (m, 1H), 5.60 (2×d, *J*=8.1 Hz, 1H), 5.53 (br s, 1H), 5.41 (br s, 1H), 4.73–4.69 (m, 1H), 4.67–4.61 (m, 1H), 4.17–4.13 (m, 1H), 4.10 (br s, 1H), 3.97 (br s, 1H). ^13^C NMR (75 MHz, DMSO‐d6): δ 163.0, 150.69, 150.67, 150.3, 150.3, 149.22, 149.19, 141.02, 140.97, 139.23, 139.21, 138.90, 138.89, 124.3, 124.1, 123.5, 123.22, 123.19, 122.4, 120.50, 120.45, 102.2, 102.1, 88.5, 88.4, 81.8, 81.7, 72.03, 71.98, 70.5, 64.7, 64.6, 51.23, 51.19; HRMS (ESI) *m/z* calcd for C_20_H_19_N_5_O_6_SNa [M+Na]^+^: 480.0954; found 480.0972; ${\left[\alpha \right]}_{D}^{25}$ =29.1 (c 0.5 in MeOH).

**5′‐Deoxy‐5′‐(4′′‐(α‐hydroxy‐α‐(N‐tert‐Butoxycarbonylindol‐3′′′‐ylmethyl)triazolo)uridine (29** **g)**. From the acetyl‐protected 1,2,3‐triazole **28** **g** (300 mg, 450 μmol), the title compound was obtained as an off‐white powder (202 mg, 83 %). m.p. 215–216 °C; ^1^H NMR (500 MHz, DMSO‐d6): δ 11.34 (br s, 1H), 8.05–8.03 (m, 1H), 7.58–7.57 (m, 1H), 7.52–7.49 (m, 1H), 7.46–7.44 (m, 1H), 7.30–7.27 (m, 2H), 7.17–7.12 (m, 1H), 6.07 (br s 1H), 6.03–6.01 (m, 1H), 5.78–5.72 (m, 1H), 5.59–5.55 (m, 1H), 5.50 (br s, 1H), 5.38 (br s, 1H), 4.71–4.66 (m, 1H), 4.64–4.58 (m, 1H), 4.15–4.09 (m, 1H), 4.03–4.00 (m, 1H), 3.96–3.95 (m, 1H), 1.62 (s, 9H). ^13^C NMR (75 MHz, DMSO‐d6): δ 162.9, 150.65, 150.63, 150.29, 150.26, 149.1, 140.9, 135.1, 128.33, 128.30, 124.3, 123.7, 123.6, 123.3, 123.2, 122.74, 122.69, 122.4, 122.3, 120.5, 114.67, 114.66, 102.08, 102.05, 88.5, 88.3, 83.7, 81.83, 81.78, 72.1, 72.0, 70.53, 70.50, 61.8, 51.2, 51.1, 27.7. HRMS (ESI) *m/z* calcd for C_25_H_28_N_6_O_8_Na [M+Na]^+^: 563.1866; found 563.1891; ${\left[\alpha \right]}_{D}^{20}$ =38.3 (c 0.5 in MeOH).

**5′‐(4′′‐(N‐tert‐Butoxycarbonylindol‐3′′′‐yl)‐α‐carboxamido‐α‐hydroxymethyltriazolo)‐5′‐deoxyuridine (29** **h)**. From the acetyl‐protected 1,2,3‐triazole **28** **h** (250 mg, 366 μmol), the title compound was obtained as an off‐white powder (173 mg, 79 %). m.p. decomp. >280 °C; ^1^H NMR (500 MHz, DMSO‐d6): δ 8.06–8.04 (m, 1H), 8.01–7.97 (m, 1H), 7.55 (d, *J*=9.1 Hz, 1H), 7.47–7.39 (m, 2H), 7.31–7.26 (m, 1H), 7.15–7.10 (m, 1H), 6.85 (s, 1H), 5.76–5.75 (m, 1H), 5.58–5.56 (m, 1H), 5.51–5.50 (m, 1H), 5.39–5.38 (m, 1H), 4.74–4.73 (m, 1H), 4.67–4.63 (m, 1H), 4.18–4.14 (m, 1H), 4.01–3.96 (m, 2H), 3.68 (2×s, 3H), 1.62 (s, 9H). ^13^C NMR (125 MHz, DMSO‐d6): δ 172.0, 162.9, 150.7, 150.6, 148.9, 148.6, 140.8, 137.8, 137.7, 135.1, 133.2, 128.0, 124.4, 124.3, 124.1, 122.4, 122.1, 122.0, 114.6, 102.1, 88.4, 88.3, 84.05, 84.03, 81.8, 81.7, 72.59, 72.55, 72.1, 72.0, 70.51, 70.46, 52.6, 51.2, 27.6; HRMS (ESI) *m/z* calcd for C_27_H_30_N_6_O_10_Na [M+Na]^+^: 621.1921; found 621.1930; ${\left[\alpha \right]}_{D}^{20}$ =36.3 (c 0.5 in MeOH).

**5′‐(4′′‐(α‐Hydroxy‐α‐trifluoromethylbenzyl)triazolo)‐5′‐deoxyuridine (29** **i)**. From the acetyl‐protected 1,2,3‐triazole **28** **i** (300 mg, 504 μmol), the title compound was obtained as a white powder (203 mg, 86 %). m.p. 122–123 °C; ^1^H NMR (500 MHz, DMSO‐d6): δ 11.35 (br s, 1H), 8.13 (2×s, 1H), 7.62–7.59 (m, 2H), 7.56–7.37 (m, 4H), 5.76–5.74 (m, 1H), 5.61–5.45 (m, 4H), 4.79–4.67 (m, 2H), 4.21–4.15 (m, 1H), 4.09–3.96 (m, 2H); ^13^C NMR (125 MHz, DMSO‐d6): δ 162.98, 162.95, 150.7, 146.7, 146.6, 141.0–140.9 (m), 137.9, 137.8, 127.8 (q, ^*1*^ *J*_*C, F*_=284.0 Hz), 127.24, 127.20, 125.1, 125.0, 102.12, 102.09, 88.7, 88.6, 81.7, 81.6, 74.3 (q, ^*2*^ *J*_*C, F*_=27.0 Hz), 72.1, 72.0, 70.6, 70.5, 51.34, 51.26; HRMS (ESI) *m/z* calcd for C_19_H_18_FN_5_O_6_Na [M+Na]^+^: 492.1107; found 492.1123. Δ=3.25 ppm; ${\left[\alpha \right]}_{D}^{25}$ =18.7 (c 0.5 in MeOH).

**5′‐Deoxy‐5′‐(4′′‐(α‐hydroxy‐3′′′‐phenoxybenzyl)triazolo)‐5‐fluorouridine (31** **a)**. From the acetyl‐protected 1,2,3‐triazole **30** **a** (260 mg, 408 μmol), the title compound was obtained as a white powder (204 mg, 98 %). m.p. 193–195 °C; ^1^H NMR (500 MHz, DMSO‐d6): δ 11.80 (br s, 1H), 8.00–7.94 (m, 1H), 7.87–7.86 (m, 1H), 7.40–7.37 (m, 2H), 7.34–7.30 (m, 1H), 7.15–7.12 (m, 2H), 7.08–7.07 (m, 1H), 7.01–6.99 (m, 2H), 6.88–6.86 (m, 1H), 6.06–6.03 (m, 1H), 5.80 (s, 1H), 5.76–5.75 (m, 1H), 5.53 (br s, 1H), 5.39 (br s, 1H), 4.71–4.62 (m, 2H), 4.15–4.09 (m, 2H), 3.97–3.96 (m, 1H); ^13^C NMR (125 MHz, DMSO‐d6): δ 157.00 (2×d, J=26.2 Hz), 156.6, 156.5, 151.3, 151.2, 149.4, 146.33, 146.32, 140.2 (2×d, *J*=231.9 Hz), 130.0, 129.7, 129.6, 125.4 (2×d, *J*=34.5 Hz), 123.5, 123.4, 122.8, 122.7, 121.43, 121.41, 118.67, 118.65, 117.1, 116.4, 116.3, 88.64, 88.58, 82.0, 81.9, 72.01, 71.97, 70.5, 67.5, 67.4, 51.23, 51.19; HRMS (ESI) *m/z* calcd for C_24_H_22_FN_5_O_7_Na [M+Na]^+^: 534.1401; found 534.1412; ${\left[\alpha \right]}_{D}^{20}$ =25.5 (c 0.5 in MeOH).

**5′‐Deoxy‐5′‐(4′′‐(α‐hydroxy‐3′′′‐phenoxybenzyl)triazolo)thymidine (31** **b)**. From the acetyl‐protected 1,2,3‐triazole **30** **b** (280 mg, 486 μmol), the title compound was obtained as a white powder (232 mg, 97 %). m.p. 218–220 °C; ^1^H NMR (300 MHz, DMSO‐d6): δ 11.27 (br s, 1H), 7.85 (d, J=8.8 Hz, 1H), 7.39–7.36 (m, 2H), 7.32–7.30 (m, 2H), 7.14–7.11 (m, 2H), 7.06 (br s, 1H), 6.99 (br s, 2H), 6.86 (d, J=7.8 Hz, 1H), 6.16–6.15 (m, 1H), 6.04 (br s, 1H), 5.80 (br s, 1H), 5.48 (br s, 1H), 4.68–4.65 (m, 1H), 4.60–4.54 (m, 1H), 4.26 (br s, 1H), 4.07 (br s, 1H), 2.13–2.07 (m, 2H), 1.77 (2×s, 3H); ^13^C NMR (75 MHz, DMSO‐d6): δ 171.5, 163.7, 156.6, 151.3, 151.2, 150.4, 146.40, 146.37, 136.1, 135.9, 130.0, 129.7, 123.5, 122.81, 122.76, 121.5, 118.69, 118.66, 117.2, 117.1, 116.4, 116.3, 110.0, 84.14, 84.09, 84.0, 70.9, 67.51, 67.46, 51.2, 37.9, 12.1; HRMS (ESI) *m/z* calcd for C_25_H_25_N_5_O_6_Na [M+Na]^+^: 514.1703; found 514.1722; ${\left[\alpha \right]}_{D}^{20}$ =50.0 (c 0.5 in MeOH).

**2′,5′‐Dideoxy‐5′‐(4′′‐(α‐hydroxy‐3′′′‐phenoxybenzyl)triazolo)uridine (31** **c)**. From the acetyl‐protected 1,2,3‐triazole **30** **c** (250 mg, 445 μmol), the title compound was obtained as a white powder (196 mg, 92 %). m.p. 232–233 °C; ^1^H NMR (500 MHz, DMSO‐d6): δ 11.33 (br s, 1H), 7.85–7.84 (m, 1H), 7.53–7.47 (m, 1H), 7.39–7.36 (m, 2H), 7.33–7.30 (m, 1H), 7.15–7.12 (m, 2H), 7.07 (s, 1H), 7.00‐6.99 (m, 2H), 6.88–6.86 (m, 1H), 6.15–6.13 (m, 1H), 6.04–6.03 (m, 1H), 5.80–5.79 (m, 1H), 5.59–5.56 (m, 1H), 5.50–5.49 (m, 1H), 4.67–4.63 (m, 1H), 4.58–4.53 (m, 1H), 4.24–4.23 (m, 1H), 4.08–4.04 (m, 1H), 2.12–2.08 (m, 2H); ^13^C NMR (125 MHz, DMSO‐d6): δ 163.0, 156.54, 156.52, 151.2, 151.1, 150.4, 146.3, 140.7, 140.6, 130.0, 129.6, 123.5, 123.4, 122.8, 121.42, 121.40, 118.7, 118.6, 117.09, 117.06, 116.32, 116.27, 102.1, 84.4, 84.3, 84.2, 84.1, 70.8, 70.7, 67.4, 51.20, 51.15, 38.1; HRMS (ESI) *m/z* calcd for C_24_H_23_N_5_O_6_Na [M+Na]^+^: 500.1546; found 500.1583; ${\left[\alpha \right]}_{D}^{20}$ =51.2 (c 0.5 in MeOH).

**5′‐(4′′‐(α‐hydroxy‐3′′′‐phenoxybenzyl)triazolo)‐2′, 5′‐dideoxy‐2′‐fluorouridine (31** **d)**. From the acetyl‐protected 1,2,3‐triazole **30** **d** (250 mg, 431 μmol), the title compound was obtained as a white powder (205 mg, 96 %). m.p. 164–166 °C; ^1^H NMR (500 MHz, DMSO‐d6): δ 11.39 (br s, 1H), 7.89–7.87 (m, 1H), 7.39–7.31 (m, 4H), 7.16–7.11 (m, 2H), 7.09 (br s, 1H), 7.01‐6.99 (m, 2H), 6.88–6.86 (m, 1H), 6.04 (br s, 1H), 5.90 (br s, 1H), 5.86–5.80 (m, 2H), 5.57–5.53 (m, 1H), 5.21–5.08 (m, 1H), 4.76–4.73 (m, 1H), 4.66–4.61 (m, 1H), 4.17–4.15 (m, 2H); ^13^C NMR (125 MHz, DMSO‐d6): δ 163.07, 163.06, 156.53, 156.51, 151.24, 151.22, 150.08, 150.07, 146.3, 141.3, 141.2, 130.0, 129.6, 123.4, 123.01, 122.95, 121.5, 121.4, 118.7, 117.1, 116.4, 116.3, 102.0, 101.9 92.6 (2×d, *J*=185.0 Hz), 89.1 (2×d, *J*=13.8 Hz), 80.2, 80.1, 69.6 (2×d, *J*=16.3 Hz), 67.41, 67.39, 50.64, 50.57; HRMS (ESI) *m/z* calcd for C_24_H_22_FN_5_O_6_Na [M+Na]^+^: 518.1452; found 518.1459, ${\left[\alpha \right]}_{D}^{20}$ =73.0 (c 0.5 in MeOH).

**5′‐Deoxy‐5′‐(4′′‐(α‐hydroxy‐3′′′‐phenoxybenzyl)triazolo)‐2′‐*O‐*methyluridine (31** **e)**. From the acetyl‐protected 1,2,3‐triazole **30** **e** (180 mg, 304 μmol), the title compound was obtained as a white powder (147 mg, 95 %). m.p. 205–207 °C; ^1^H NMR (500 MHz, DMSO‐d6): δ 11.39 (br s, 1H), 7.88–7.87 (m, 1H), 7.45 (2×d, J=8.2 Hz, 1H), 7.39–7.36 (m, 2H), 7.33–7.30 (m, 1H), 7.15–7.11 (m, 2H), 7.08 (br s, 1H), 7.00–6.98 (m, 2H), 6.87–6.85 (m, 1H), 6.08–6.07 (m, 1H), 5.81–5.80 (m, 2H), 5.59–5.56 (m, 1H), 5.55–5.53 (m, 1H), 4.72–4.68 (m, 1H), 4.65–4.60 (m, 1H), 4.16–4.08 (m, 3H), 3.83–3.77 (m, 1H), 3.33 (s, 3H); ^13^C NMR (75 MHz, DMSO‐d6): δ 162.87, 162.85, 156.5, 151.2, 150.4, 150.3, 146.3, 140.6, 140.5, 130.0, 129.6, 123.4, 122.9, 121.4, 118.6, 117.02, 116.31, 116.28, 102.17, 102.15, 87.1, 86.9, 81.92, 81.86, 81.14, 81.09, 69.32, 69.26, 67.4, 57.7, 57.6, 51.0, 50.9; HRMS (ESI) *m/z* calcd for C_25_H_25_N_5_O_7_Na [M+Na]^+^: 530.1652; found 530.1674; ${\left[\alpha \right]}_{D}^{20}$ =56.2 (c 0.5 in MeOH).

### Biological Evaluation

Biological assessment of the activity of these compounds against human ST3GAL1 and ST6GAL1 was carried out based on the CMP‐Glo™ assay developed by Promega and detailed by Das *et al*.^[67]^ CMP‐Neu5Ac was used as the donor and Gal‐β1,3‐GalNAc and LacNAc were used as the acceptors for ST3GAL1 and ST6GAL1, respectively. CMP produced as a by‐product of the ST reaction was detected using a luciferase producing luminescence with a linear response to the concentration of CMP, detected using a POLARstar Omega plate reader. The percentage inhibition of all compounds at 100 μM (with 1 mM LacNAc and 100 μM CMP‐Neu5Ac) was calculated using a positive control with no inhibitor and a negative control with no enzyme present. Reported ST inhibitors **2** and **3** were used as positive inhibition controls. ST8SIA2 activity was determined via the method of Al‐Saraireh *et al*.^[68]^ All experiments were performed as technical triplicates, and percentage inhibition is given as the average of these three results. Data shown in Table 1. The MTS cell viability assay was performed as described in our prior work (see supplementary information for detail).^[52]^

### Molecular Modelling

For docking purposes, the crystallographic coordinates of the porcine ST3GAL1, human ST6GAL1 complex with CMP, and human ST8SIA3 with a donor analogue were obtained from the RCSB Protein Data Bank (PDB code 2WNB,^[44]^ 4JS2^[41]^ and 5CXY^[42]^ respectively). The ligand was then removed to leave the receptor complex using Discovery Studio 4, which was used for the subsequent docking studies. The PDBQT files for the STs and the 5′‐triazolonucleoside derivatives were generated using Autodock Tools 1.5.6 and used as the input for AutoDock Vina 1.1.2.^[69]^ When docking, the grid scale was set as 30 Å×30 Å×30 Å based on grid module, with a spacing of 1 Å between F++grid points. The protein was kept rigid, and all torsional bonds of the ligands were kept free.

## Supporting Information

The authors have cited additional references within the Supporting Information,[^54^, ^61^, ^70^, ^71^, ^72^, ^73^] which includes experimental data for the aryl alkynes synthesised in this study, SwissADME data for the final compounds (**29** **a–i** and **31** **a–e**), in silico cytotoxicity evaluation and ^1^H and ^13^C NMR spectra for the final compounds (**29** **a–i** and **31** **a–e**).

## Conflict of Interests

The authors declare no conflict of interest.

## Supporting information

As a service to our authors and readers, this journal provides supporting information supplied by the authors. Such materials are peer reviewed and may be re‐organized for online delivery, but are not copy‐edited or typeset. Technical support issues arising from supporting information (other than missing files) should be addressed to the authors.

Supporting Information

## Data Availability

The data that support the findings of this study are available in the supplementary material of this article.
