# Supplementary material for: Synthesis of α‐Hydroxy‐1,2,3‐Triazole‐linked Sialyltransferase Inhibitors and Evaluation of Selectivity Towards ST3GAL1, ST6GAL1 and ST8SIA2
Source: ChemMedChem. 2024 Jul 1;19(16):e202400088. doi: 10.1002/cmdc.202400088 (PMC13494824; doi:10.1002/cmdc.202400088)
Supplement: Supplementary file 1 [file CMDC-19-e202400088-s001.pdf]

ChemMedChem

Supporting Information

Synthesis of α-Hydroxy-1,2,3-Triazole-linked Sialyltransferase Inhibitors and Evaluation of Selectivity Towards ST3GAL1, ST6GAL1 and ST8SIA2

Rémi Szabo, Chris Dobie, Andrew P. Montgomery, Harrison Steele, Haibo Yu, and Danielle Skropeta*

Supporting Information

Synthesis of α-Hydroxy-1,2,3-Triazole-linked Sialyltransferase Inhibitors and Evaluation of Selectivity towards ST3GAL1, ST6GAL1 and ST8SIA2

Rémi Szabo[a], Chris Dobie,[a, b] Andrew P. Montgomery,[a] Harrison Steele,[a] Haibo Yu,[a, b, c] Danielle Skropeta*[a, b]

[a] Dr R. Szabo, Dr C. Dobie, Dr A. P. Montgomery, H. Steele, Prof H. Yu, Prof D. Skropeta
School of Chemistry & Molecular Bioscience
University of Wollongong
Wollongong, NSW, Australia
E-mail: skropeta@uow.edu.au

[b] Dr C. Dobie, Prof H. Yu, Prof D. Skropeta
Molecular Horizons
University of Wollonong
Wollongong, NSW, Australia

[c] Prof H. Yu
ARC Centre of Excellence in Quantum Biotechnology
University of Wollongong
Wollongong, NSW, Australia

Contents

Synthesis of α-arylpropargyl acetates 5a-i 2

MTS Cell viability assay 3

Table S1 Pharmacokinetic properties of final compounds and reference ST inhibitors calculated with SwissADME 4

Table S2: Predicted toxicity of final compounds using the Web-based tool ProTox 4

^{1}H and ^{13}C NMR spectra of final compounds 29a-i and 30a-e 5

Synthesis of **α**-arylpropargyl acetates (5a-i, 9 compounds).

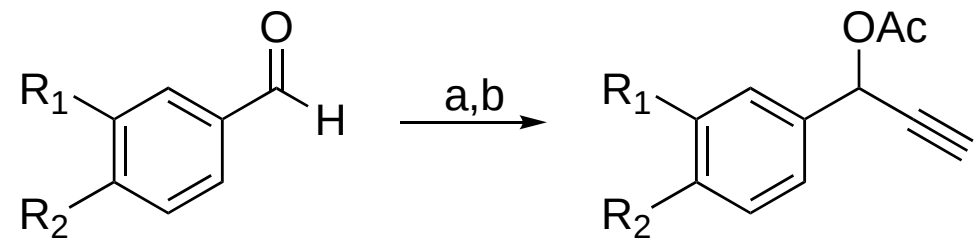

4a, R_1 = OPh; R_2 = H
4b, R_1 = OPh; R_2 = F
4c, R_1 = OCp; R_2 = H
4d, R_1 = OCp; R_2 = OMe
4e, R_1 = OPr; R_2 = H

5a, R_1 = OPh; R_2 = H
5b, R_1 = OPh; R_2 = F
5c, R_1 = OCp; R_2 = H
5d, R_1 = OCp; R_2 = OMe
5e, R_1 = OPr; R_2 = H

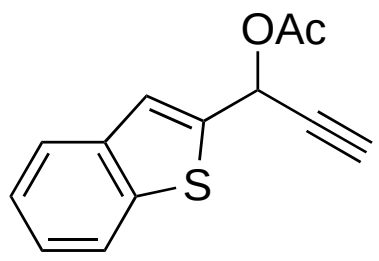

5f

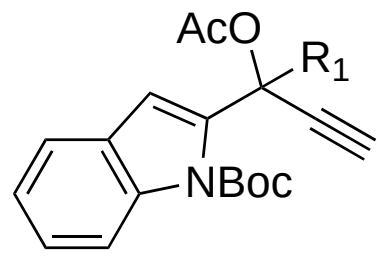

5g, R_1 = H
5h, R_1 = CO_2Me

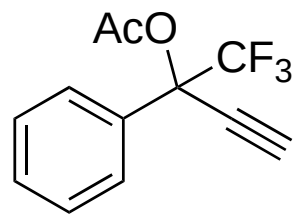

5i

Scheme S1 (Scheme 1 from paper). Synthesis of the alpha-arylpropargyl acetate derivatives **5a-e** from the corresponding aldehydes. *Reagents and conditions:* (a) Ethynyl-MgBr (1.2 equiv.), THF, 0°C. (b) Ac_2O (1.2 equiv.), Et_3N (1.5 equiv.), CH_2Cl_2, r.t, 83–94% yield over 2 steps. Heterocyclic derivatives **5f-i** were prepared in a similar manner in 51–86% yield overall from their corresponding aldehydes. Note: Cp = cyclopentyl, Pr = n-propyl.

General method. The aldehyde derivative (1.0 equiv.) was dissolved in dry THF (20 mL) and added at 0 °C to ethynyl magnesium bromide (0.5M in THF, 1.2 equiv.). After stirring at r.t. for 3 h the reaction mixture was quenched with saturated NH_4Cl solution (50 mL) and ice (50 mL). After removal of THF, Et_2O (50 mL) was added and the organic layer separated, washed with brine, dried over $MgSO_4$, filtered and concentrated to dryness. Next, the crude propargylic alcohol, Ac_2O (1.5 equiv.) and Et_3N (1.5 equiv.) in dry CH_2Cl_2 were stirred overnight at r.t (if necessary a catalytic amount of DMAP was added to achieve total conversion). The reaction mixture was washed with water, saturated $NaHCO_3$ and brine then dried over $MgSO_4$, filtered and evaporated to dryness. If necessary, the residue was purified by silica gel column chromatography or used without further purification. [1]

1-(3′-Phenoxyphenyl)prop-2-ynyl acetate (5a). The title derivative was obtained starting from 3-phenoxybenzaldehyde (400 mg, 2.43mmol) as an amber oil (520 mg, 92%), with spectral data matching those reported. [2]

1-(4′-Fluoro-3′-phenoxyphenyl)prop-2-ynyl acetate (5b). From 4-fluoro-3-phenoxybenzaldehyde (400 mg, 1.85 mmol), the title compound was obtained as an amber oil (494 mg, 94%). ^{1}H NMR (300 MHz, $CDCl_3$): δ 7.37–7.27 (m, 3H), 7.24–7.09 (m, 3H) 7.00-6.98 (m, 2H), 6.36 (d, *J* = 2.0 Hz, 1H), 2.63 (d, *J* = 2.2 Hz, 1H), 2.09 (s, 3H). ^{13}C NMR (125 MHz, $CDCl_3$): δ 169.5, 157.0, 154.5 (d, *J* = 251.6 Hz), 143.8 (d, *J* = 12.7 Hz), 133.4 (d, *J* = 3.7 Hz), 130.0, 129.8, 124.1 (d, *J* = 8.4 Hz), 123.4, 121.3 (d, *J* = 1.8 Hz), 117.9, 117.3, 117.2, 79.7, 75.8, 64.3, 20.9.

1-(3′-Cyclopentoxyphenyl)prop-2-ynyl acetate (5c). From 3-cyclopentoxybenzaldehyde (600 mg, 3.15 mmol), the title compound was obtained as a clear oil (730 mg, 90%). ^{1}H NMR (500 MHz, $CDCl_3$): δ 7.29–7.26 (m, 1H), 7.08–7.05 (m, 2H), 6.86 (dd, *J* = 8.3 Hz, 2.4 Hz, 1H),

6.40 (d, *J* = 1.9 Hz, 1H), 4.78–4.76 (m, 1H), 2.64 (d, *J* = 1.9 Hz, 1H), 2.12 (s, 3H), 1.94–1.78 (m, 6H), 1.64–1.61 (m, 2H); ^{13}C NMR (125 MHz, $CDCl_3$): δ 169.7, 158.3, 137.7, 129.6, 119.4, 116.0, 114.9, 80.2, 79.3, 75.2, 65.2, 32.8, 24.0, 21.0.

1-(3′-Cyclopentoxy-4′-methoxyphenyl)prop-2-ynyl acetate (5d). From 3-cyclopentoxy-4-methoxybenzaldehyde (500 mg, 2.27 mmol), the title compound was obtained as an orange oil (595 mg, 86%). ^{1}H NMR (400 MHz, $CDCl_3$): δ 7.08–7.06 (m, 2H), 6.87–6.85 (m, 1H), 6.38 (d, *J* = 2.3 Hz, 1H), 4.81–4.78 (m, 1H), 3.85 (s, 3H), 2.65 (d, *J* = 2.3 Hz, 1H), 2.10 (s, 3H), 1.97–1.77 (m, 8H). ^{13}C NMR (125 MHz, $CDCl_3$): δ 169.7, 150.7, 147.8, 128.9, 120.4, 114.6, 111.7, 80.6, 80.5, 75.1, 65.3, 56.1, 32.8, 24.0, 21.0.

1-(3′-Propoxyphenyl)prop-2-ynyl acetate (5e). The title derivative was obtained from 3-propoxybenzaldehyde (400 mg, 2.43mmol) as an amber oil (520 mg, 92%), with spectral data matching those reported.

1-(Benzo[*b*]thiophen-2-yl)prop-2-ynyl acetate (5f). The title compound was obtained starting from benzo[*b*]thiophene-2-carboxaldehyde (400 mg, 2.47 mmol), as a pale yellow oil (488 mg, 86%), with spectral data matching those reported. [3]

***tert*-Butyl 3-(1-acetoxyprop-2-ynyl)-1*H*-indole-1-carboxylate (5g).** The title compound was obtained from *tert*-butyl 3-formyl-1*H*-indole-1-carboxylate (400 mg, 1.63 mmol), as an amber oil (388 mg, 51%), with spectral data matching those reported. [4]

Methyl 2-(1-*tert*-butoxycarbonyl-3-indolyl)-2-hydroxybut-3-ynoate (5h). From *tert*-butyl 3-(2-methoxy-2-oxoacetyl)-1*H*-indole-1-carboxylate (700 mg, 2.31 mmol), the title compound was obtained as an amber oil (388 mg, 51%), with spectral data matching those reported. [4]

1,1,1-trifluoro-2-phenylbut-3-yn-2-yl acetate (5i). The title compound was obtained starting from 1,1,1-trifluoroacetophenone (500 mg, 2.87 mmol) as a white solid (388 mg, 51%), with spectral data matching those reported. [5]

MTS cell viability assay

MIA PaCa-2 human pancreatic cancer cells were grown in DMEM cell culture medium with L-glutamine, supplemented with 10% FBS. Cell culture medium and supplements were obtained from Invitrogen (Australia). The cells were incubated at 37 °C under a humidified atmosphere containing 5% CO_2 in a Heracell incubator (Kendro Laboratory Products Germany). Cell viability was determined using the CellTiter 96 Aqueous One Solution (MTS) Cell Proliferation colorimetric assay. Cells were plated in 96-well plates at a density of 60,000 cells/mL and allowed to attach overnight (24 h). Samples were added at a final concentration of 200-300 μM in media and the plates incubated at 37 °C for 24–48 hrs. The MTS reagent [3-(4,5-dimethylthiazol-2-yl)-5-(3-carboxymethoxyphenyl)-2-(4-sulfophenyl)–2*H*-tetrazolium, inner salt] (50 μg) was added and the cells were incubated for another 3 h, and absorbance determined on a plate reader at 490 nm as reported previously.[6]

Table S1: Pharmacokinetic properties of final compounds and reference ST inhibitors calculated with SwissADME [6]

Compound	MW	LogP	HBA	HBD	tPSA	LogS	Lipinski Violations	PAINS
***(R)*-1**	583	-0.46	13	3	255.18	-1.82	2	N
**2**	473	-0.9	11	4	215.88	-1.47	1	N
**3**	609	-0.32	16	3	226.72	-2.24	2	N
**29a**	493	0.42	9	4	164.72	-2.66	1	N
**29b**	511	0.47	10	4	164.72	-2.82	2	N
**29c**	485	0.16	9	4	164.72	-2.31	1	N
**29d**	516	0.25	10	4	173.95	-2.39	2	N
**29e**	459	-0.13	9	4	164.72	-1.87	1	N
**29f**	457	0.01	8	4	183.73	-2.26	1	N
**29g**	541	0.08	10	4	186.72	-2.7	2	N
**29h**	599	0.05	12	4	213.02	-2.84	2	N
**29i**	469	0.1	11	4	155.49	-2.11	1	N
**31a**	495	1.49	9	3	144.49	-3.48	1	N
**31b**	492	1.53	8	3	144.49	-3.5	1	N
**31c**	477	1.14	8	3	144.49	-3.19	1	N
**31d**	495	1.24	9	3	144.49	-3.32	1	N
**31e**	508	0.88	9	3	153.72	-3.01	2	N
**Desirable ranges**	150-500	-0.7-5.0	<10	<5	20-130	-6.0-0		

Table S2: Predicted toxicity of final compounds using the Web-based tool ProTox 3[7]

Cmpd 29	SMILES	Predicted Tox	^Binding to Tox Targets	Cytotox
a	O[C@@H]([C@H]([C@H](N1C(NC(C=C1)=O)=O)O2)O)[C@H]2CN3N=NC(C(C4=CC(OC5=CC=CC=C5)=CC=C4)O)=C3	Class 4	None	Inactive
b	O[C@@H]([C@H]([C@H](N1C(NC(C=C1)=O)=O)O2)O)[C@H]2CN3N=NC(C(C4=CC(OC5=CC=CC=C5)=C(F)C=C4)O)=C3	Class 4	None	Inactive
c	O[C@@H]([C@H]([C@H](N1C(NC(C=C1)=O)=O)O2)O)[C@H]2CN3N=NC(C(C4=CC(OC5CCCC5)=CC=C4)O)=C3	Class 4	None	Inactive
d	O[C@@H]([C@H]([C@H](N1C(NC(C=C1)=O)=O)O2)O)[C@H]2CN3N=NC(C(C4=CC(OC5CCCC5)=C(OC)C=C4)O)=C3	Class 4	None	Inactive
e	O[C@@H]([C@H]([C@H](N1C(NC(C=C1)=O)=O)O2)O)[C@H]2CN3N=NC(C(C4=CC(OCCC)=CC=C4)O)=C3	Class 4	None	Inactive
f	O[C@@H]([C@H]([C@H](N1C(NC(C=C1)=O)=O)O2)O)[C@H]2CN3N=NC(C(C4=CC(C=CC=C5)=C5S4)O)=C3	Class 4	None	Inactive
g	O[C@@H]([C@H]([C@H](N1C(NC(C=C1)=O)=O)O2)O)[C@H]2CN3N=NC(C(C4=CC(C=CC=C5)=C5N4C(OC(C)(C)C)=O)O)=C3	Class 4	None	Inactive
h	O[C@@H]([C@H]([C@H](N1C(NC(C=C1)=O)=O)O2)O)[C@H]2CN3N=NC(C(C4=CC(C=CC=C5)=C5N4C(OC(C)(C)C)=O)(C(OC)=O)O)=C3	Class 5	None	Inactive
i	O[C@@H]([C@H]([C@H](N1C(NC(C=C1)=O)=O)O2)O)[C@H]2CN3N=NC(C(C4=CC=CC=C4)(C(F)(F)F)O)=C3	Class 4	None	Inactive
j	O[C@@H]([C@H]([C@H](N1C(NC(C=C1)=O)=O)O2)O)[C@H]2CN3N=NC(C(C4=CC(C=CC=C5)=C5N4)O)=C3	Class 4	None	Inactive
		Class 4	None	Inactive
**Cmpd 31**	**SMILES**			
a	O[C@@H]([C@H]([C@H](N1C(NC(C(F)=C1)=O)=O)O2)O)[C@H]2CN3N=NC(C(C4=CC(OC5=CC=CC=C5)=CC=C4)O)=C3	Class 4	None	Inactive
b	O[C@@H](C[C@H](N1C(NC(C(C)=C1)=O)=O)O2)[C@H]2CN3N=NC(C(C4=CC(OC5=CC=CC=C5)=CC=C4)O)=C3	Class 6	None	Inactive
c	O[C@@H](C[C@H](N1C(NC(C=C1)=O)=O)O2)[C@H]2CN3N=NC(C(C4=CC(OC5=CC=CC=C5)=CC=C4)O)=C3	Class 4	None	Inactive
d	O[C@@H](C(F)[C@H](N1C(NC(C=C1)=O)=O)O2)[C@H]2CN3N=NC(C(C4=CC(OC5=CC=CC=C5)=CC=C4)O)=C3	Class 6	None	Inactive
e	O[C@@H]([C@H]([C@H](N1C(NC(C=C1)=O)=O)O2)OC)[C@H]2CN3N=NC(C(C4=CC(OC5=CC=CC=C5)=CC=C4)O)=C3	Class 4	None	Inactive
**ProTox**	https://tox.charite.de/protox3			
**Tox Tar**	AA2AR, Adenosine receptor A2a			
	ADRB2, Beta-2 adrenergic receptor			
	ANDR, Androgen receptor			
	AOFA, Amine oxidase [flavin-containing]			
	CRFR1, Corticotropin-releasing factor receptor			
	DRD3, D(3) dopamine receptor			
	ESR1, Estrogen receptor			
	ESR2, Estrogen receptor beta			
	GCR, Glucocorticoid receptor			
	HRH1, Histamine H1 receptor			
	NR1I2, Nuclear receptor subfamily 1 group I member 2			
	OPRK, Kappa-type opioid receptor			
	OPRM, Mu-type opioid receptor			
	PDE4D, 3',5'-cyclic-AMP phosphodiesterase 4D			
	PGH1, Prostaglandin G/H synthase 1			
	PRGR, Progesterone receptor			

Final Deprotected Triazoles:

5′-Deoxy-5′-(4′′-(α-hydroxy-3′′′-phenoxybenzyl)triazolo)uridine (29a)

^{1}H NMR

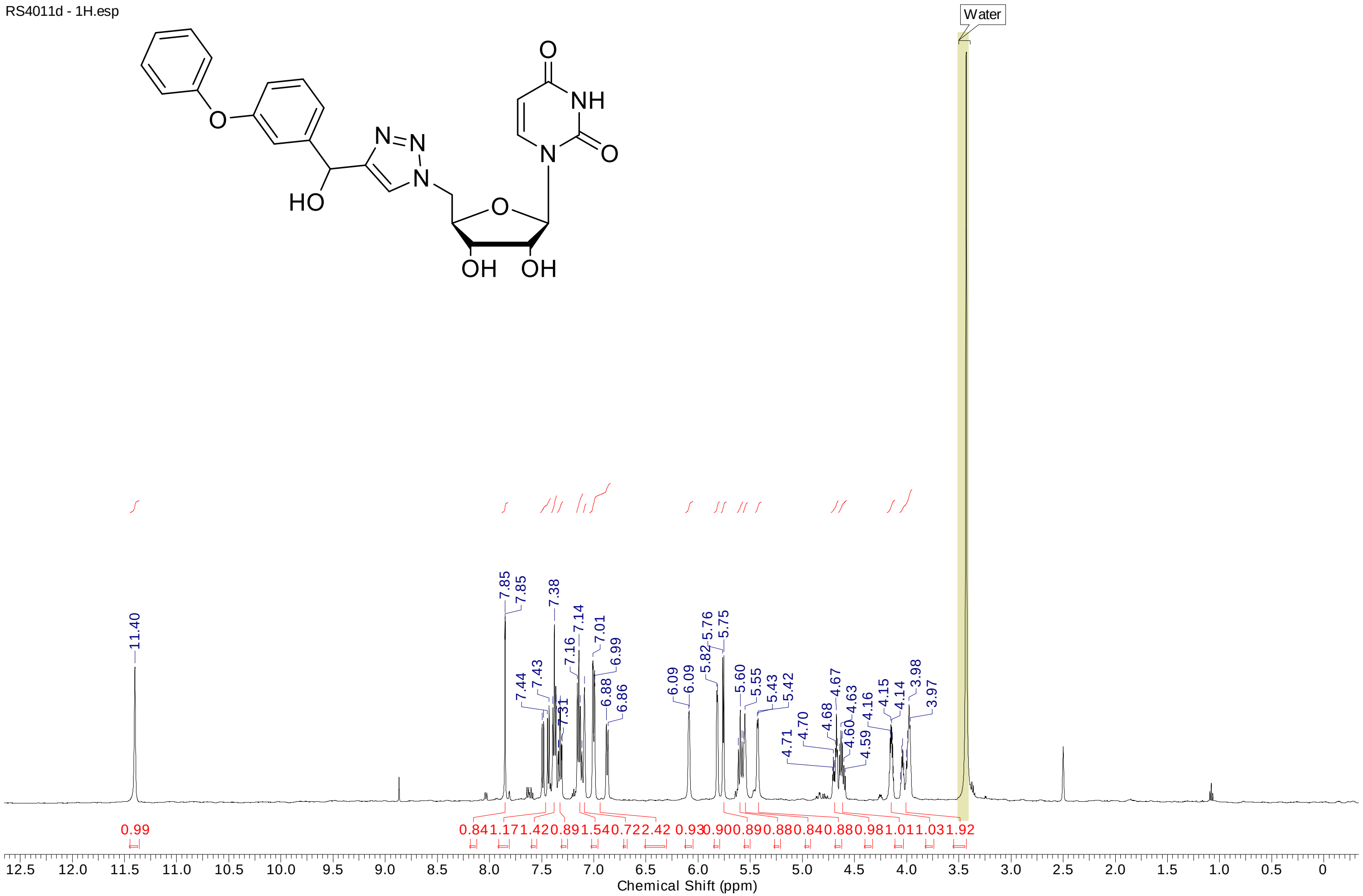

^{13}C NMR

RS4011d - 13C.esp

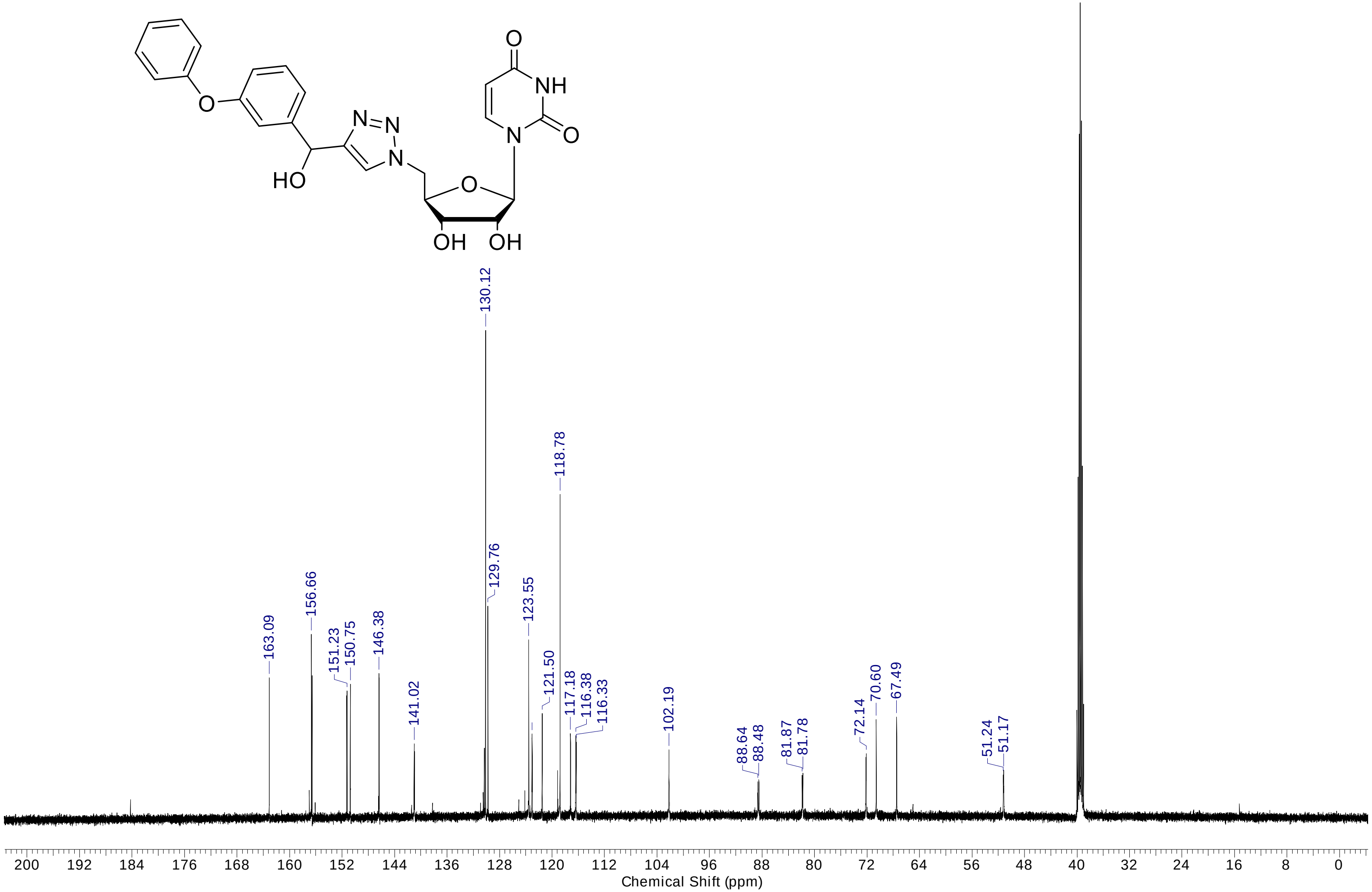

5′-Deoxy-5′-(4′′-(α-hydroxy-4′′′-fluoro-3′′′-phenoxybenzyl)triazolo)uridine (29b)

<u>^{1}H NMR</u>

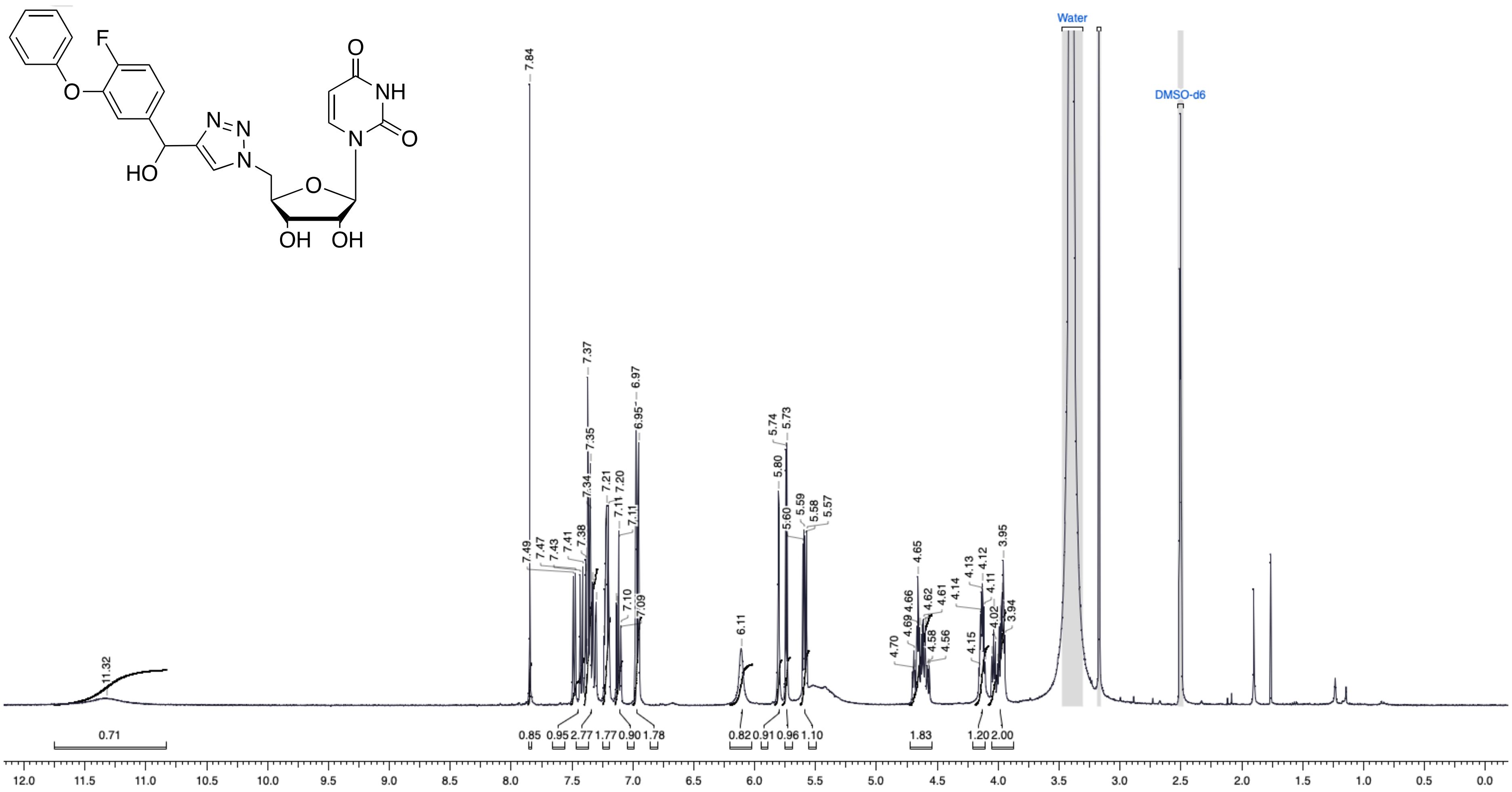

^{13}C NMR

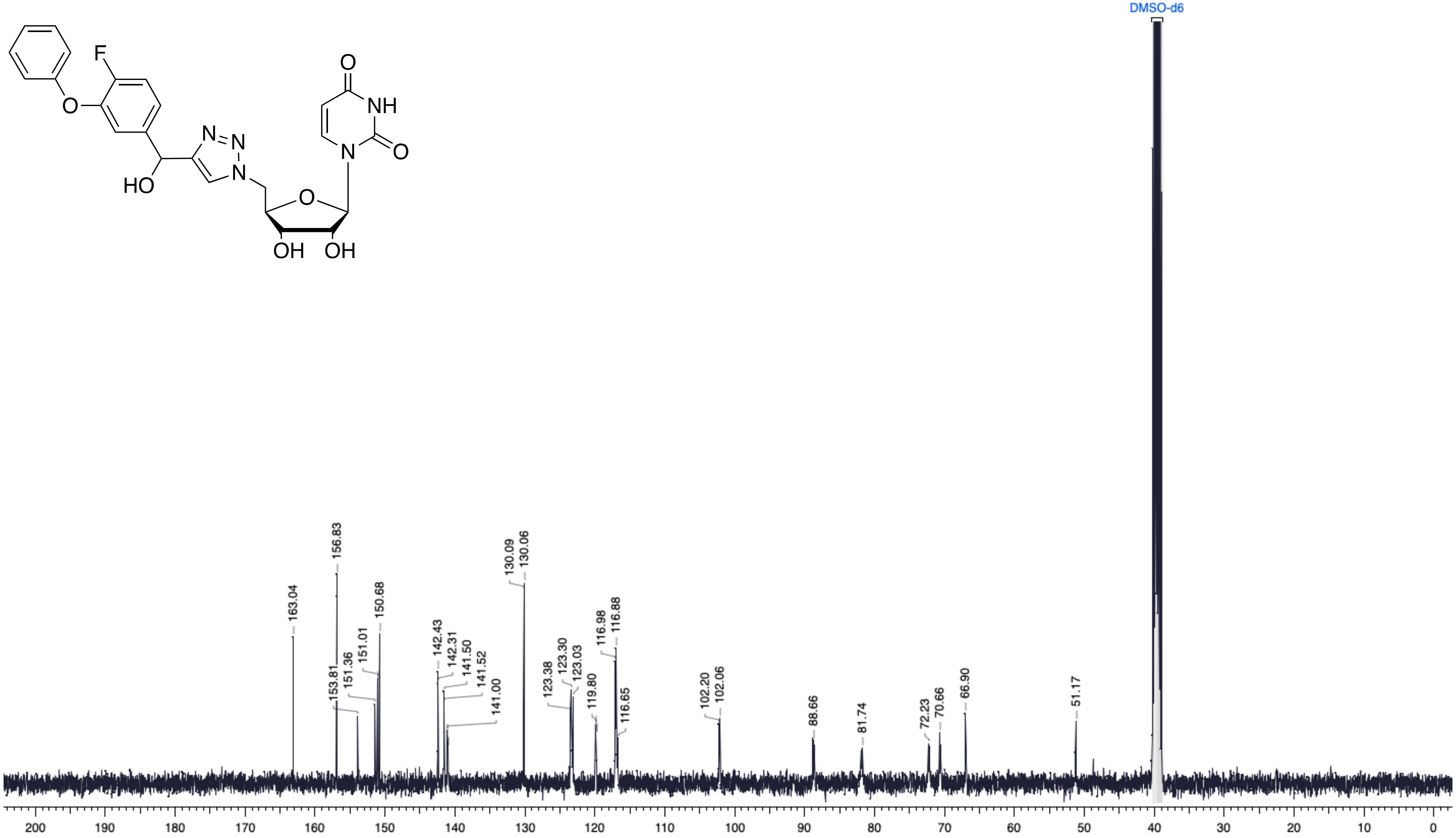
F
O
HO
N
N
N
O
NH
N
O
O
OH
OH
DMSO-d6
163.04
156.83
153.81
151.36
151.01
150.68
142.43
142.31
141.50
141.52
141.00
130.09
130.06
123.38
123.30
123.03
119.80
116.98
116.88
116.65
102.20
102.06
88.66
81.74
72.23
70.66
66.90
51.17
200
190
180
170
160
150
140
130
120
110
100
90
80
70
60
50
40
30
20
10
0

5′-Deoxy-5′-(4′′-(α-hydroxy-3′′′-cyclopentoxybenzyl)triazolo)uridine (29c)

^{1}H NMR

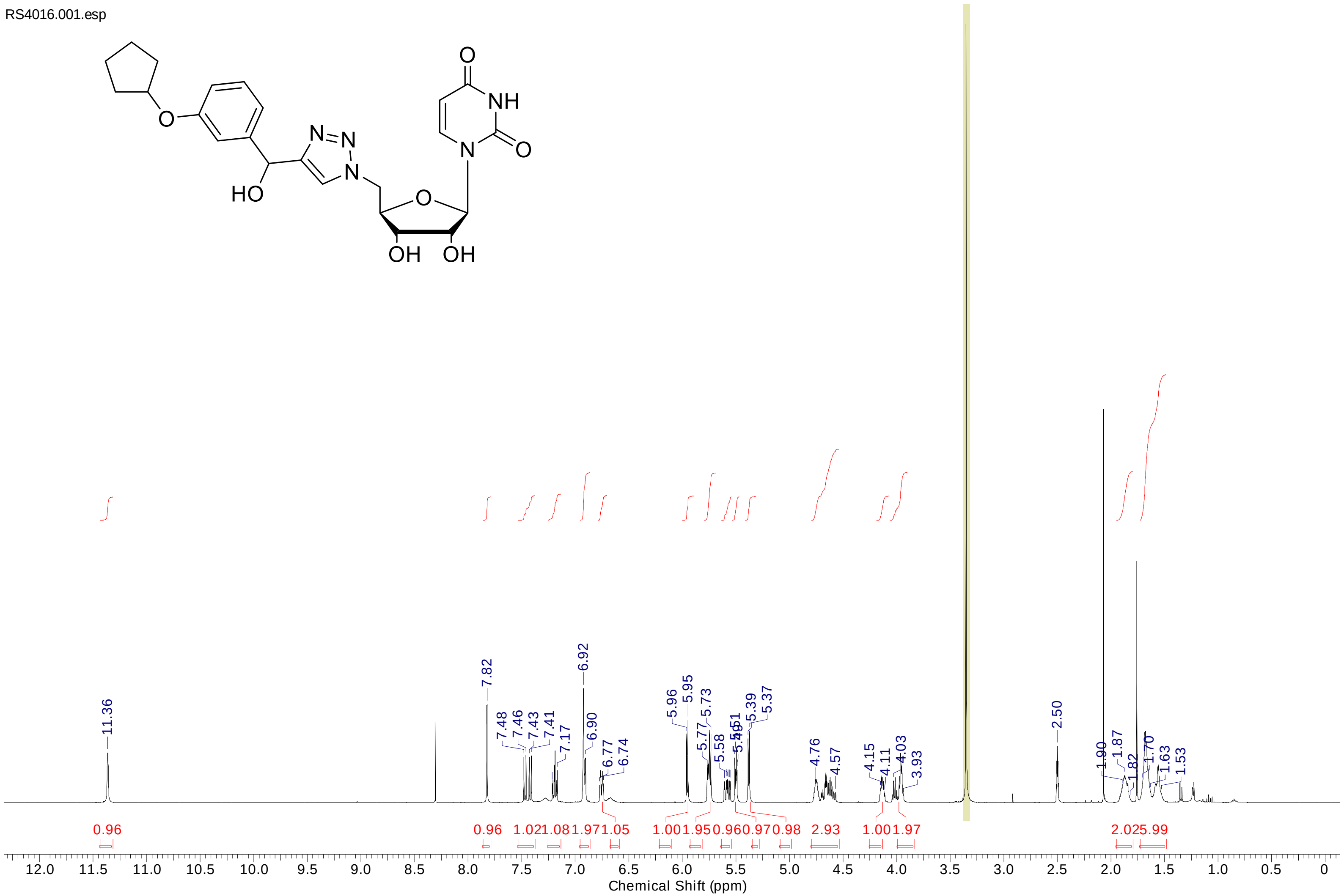

^{13}C NMR

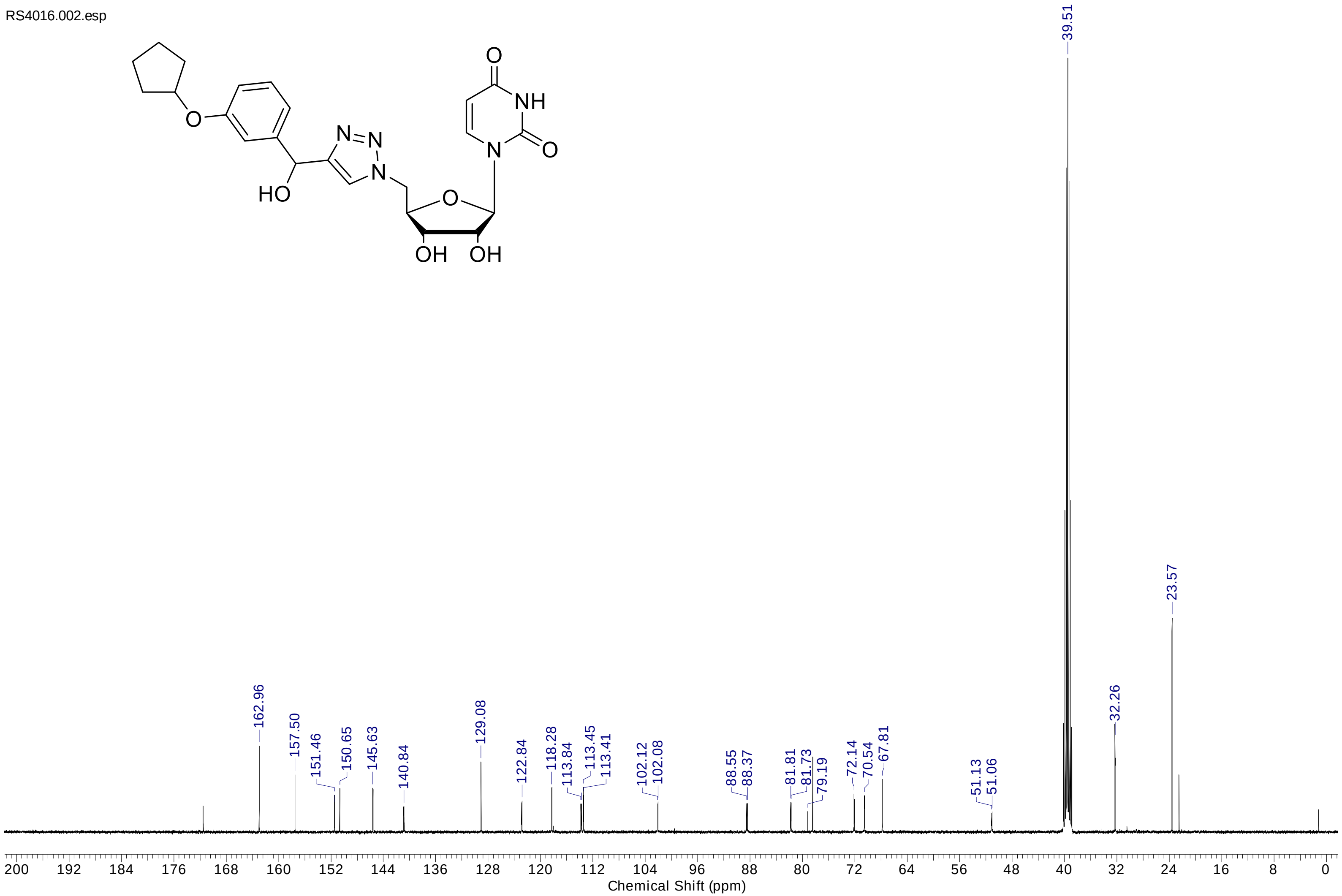
RS4016.002.esp
O
NH
O
N
N=N
N
O
HO
OH OH
39.51
23.57
32.26
162.96
157.50
151.46
150.65
145.63
140.84
129.08
122.84
118.28
113.84
113.45
113.41
102.12
102.08
88.55
88.37
81.81
81.73
79.19
72.14
70.54
67.81
51.13
51.06
200 192 184 176 168 160 152 144 136 128 120 112 104 96 88 80 72 64 56 48 40 32 24 16 8 0
Chemical Shift (ppm)

5′-Deoxy-5′-(4′′-(α-hydroxy-4′′′-methoxy-3′′′-cyclopentoxybenzyl)triazolo)uridine (29d)

<u>^{1}H NMR</u>

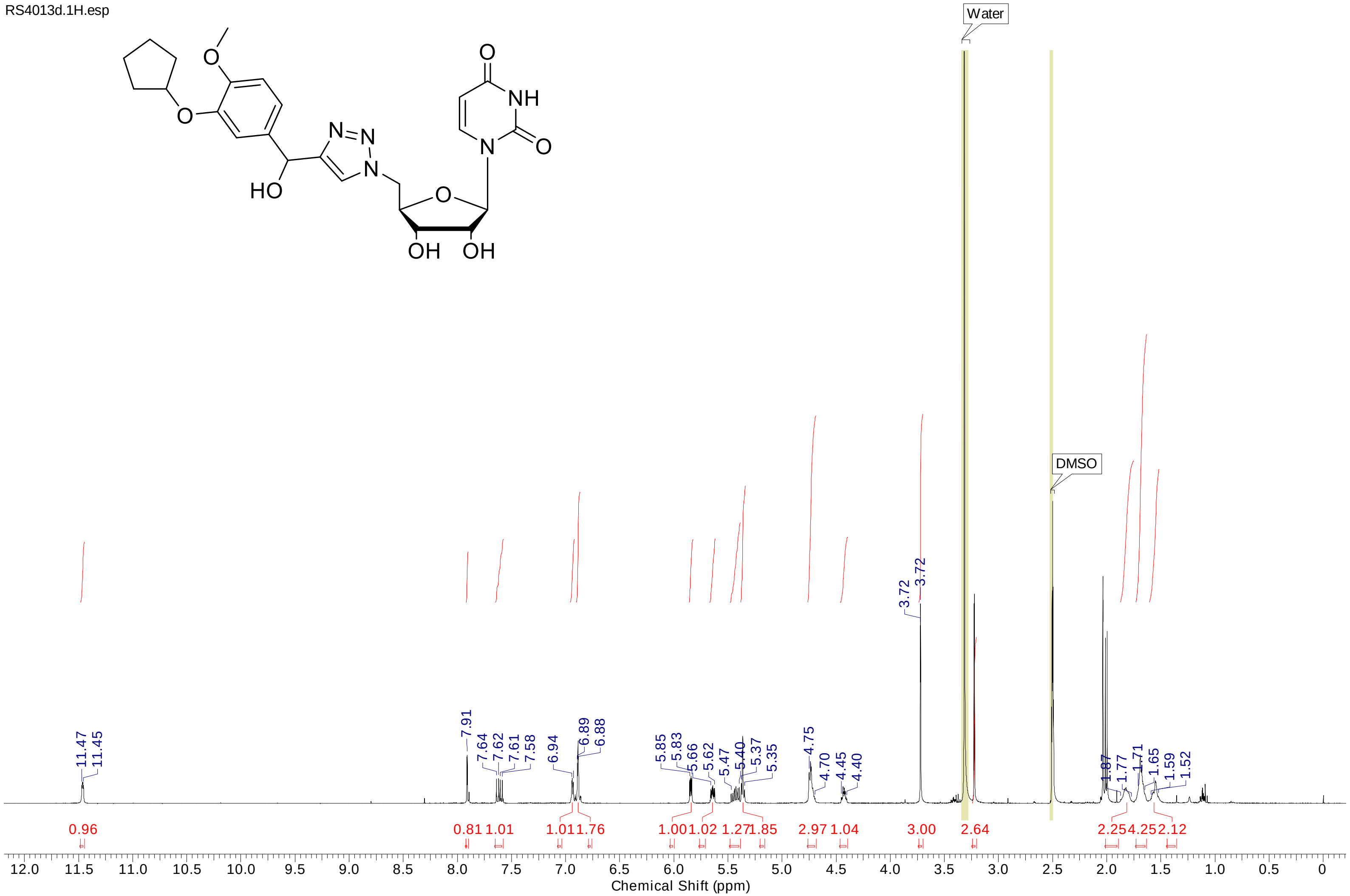

^{13}C NMR

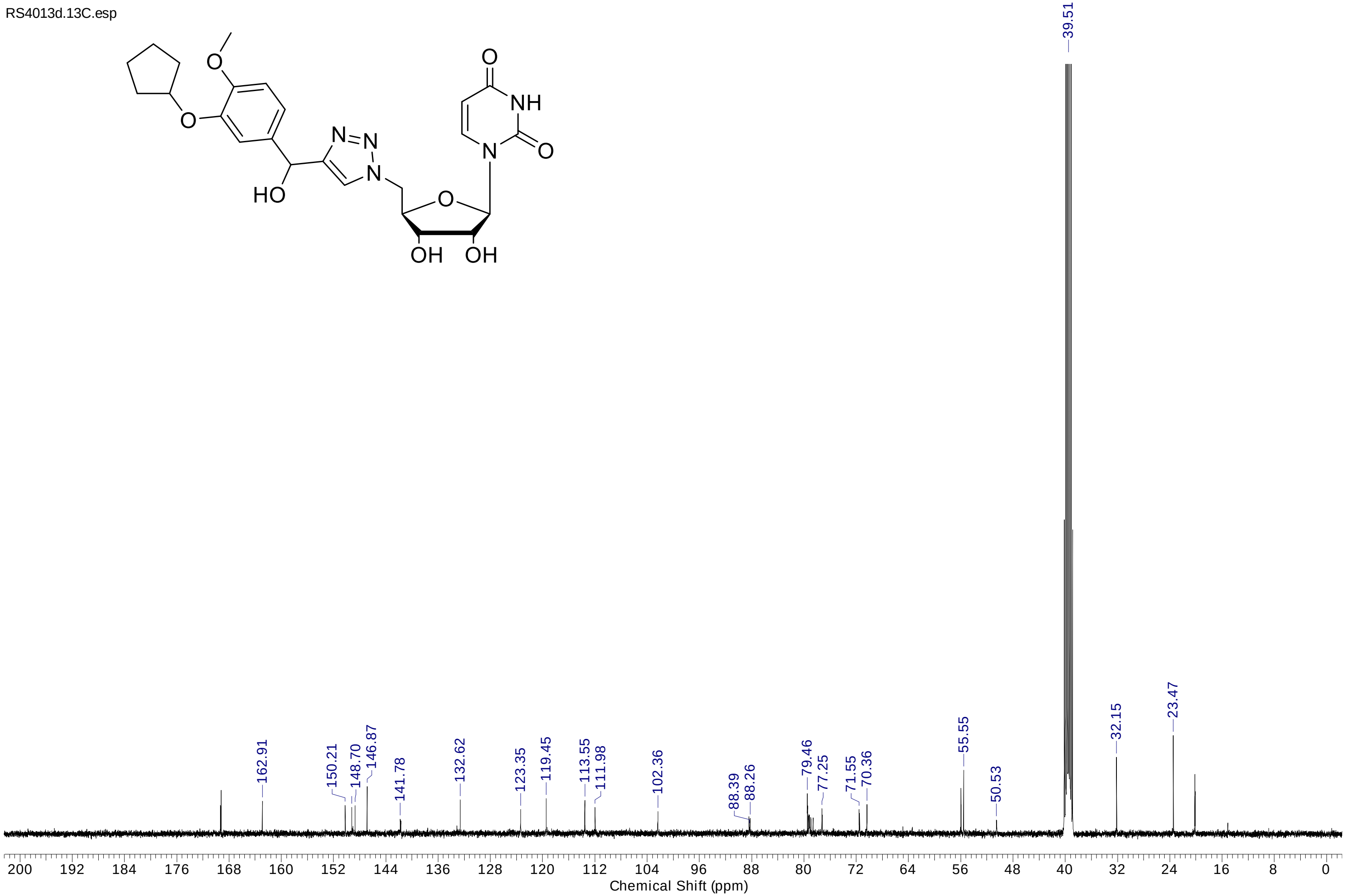
RS4013d.13C.esp
O
NH
N
O
N
N
N
O
HO
OH
OH
O
O
39.51
162.91
150.21
148.70
146.87
141.78
132.62
123.35
119.45
113.55
111.98
102.36
88.39
88.26
79.46
77.25
71.55
70.36
55.55
50.53
32.15
23.47
200 192 184 176 168 160 152 144 136 128 120 112 104 96 88 80 72 64 56 48 40 32 24 16 8 0
Chemical Shift (ppm)

5′-Deoxy-5′-(4″-(α-hydroxy-3‴-propoxybenzyl)triazolo)uridine (29e)

^{1}H NMR

RS4017 - 1H.esp

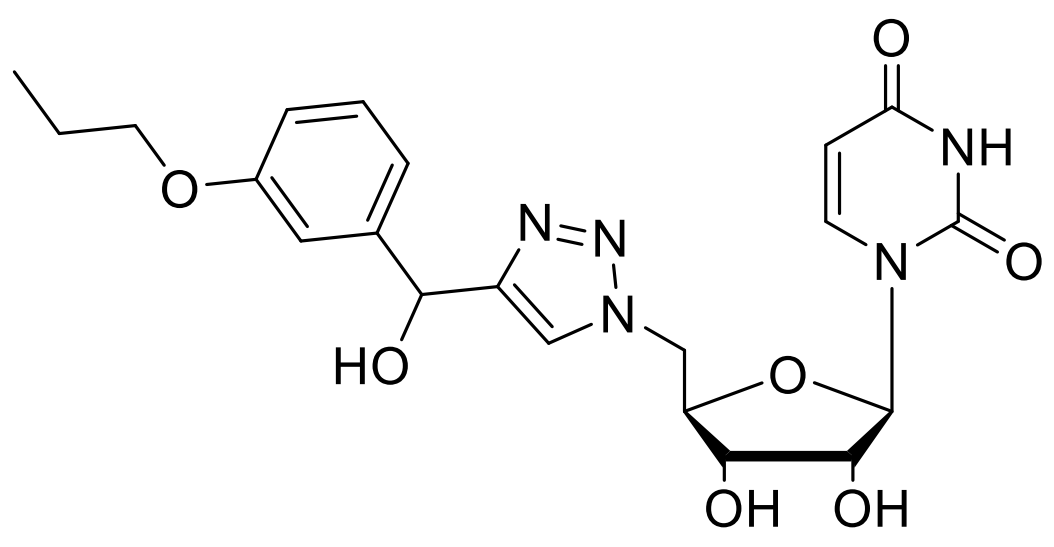

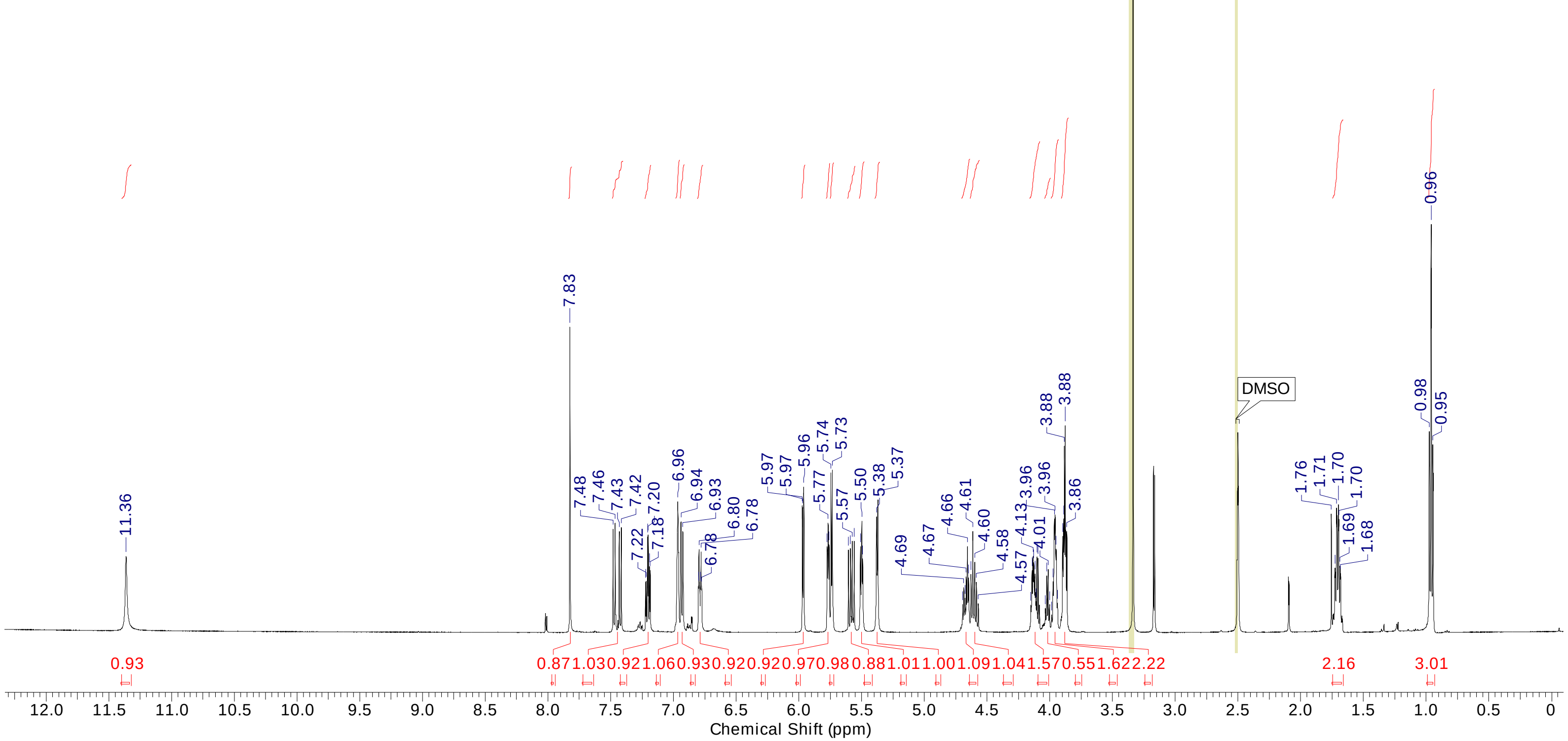

^{13}C NMR

RS4017 - 13.esp

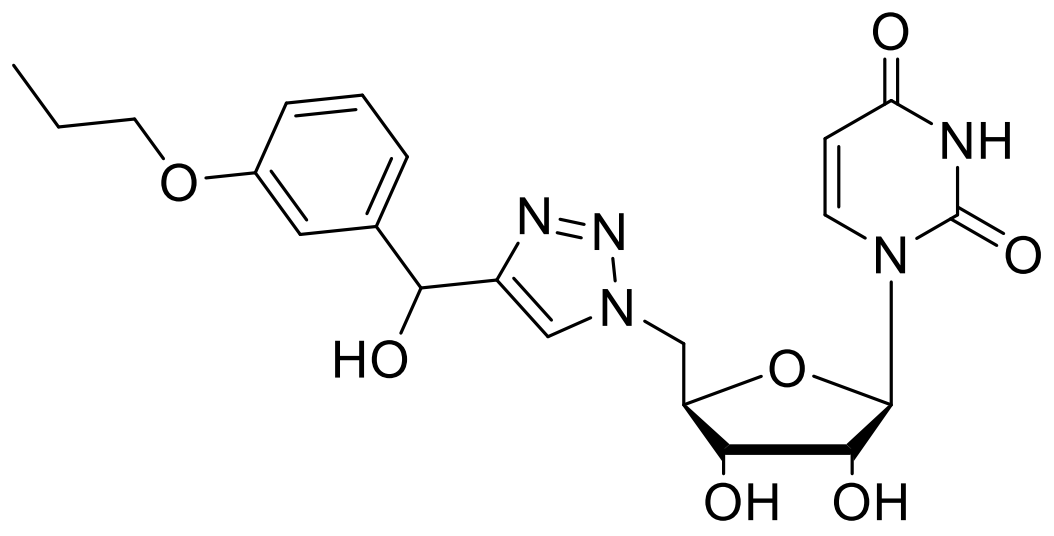

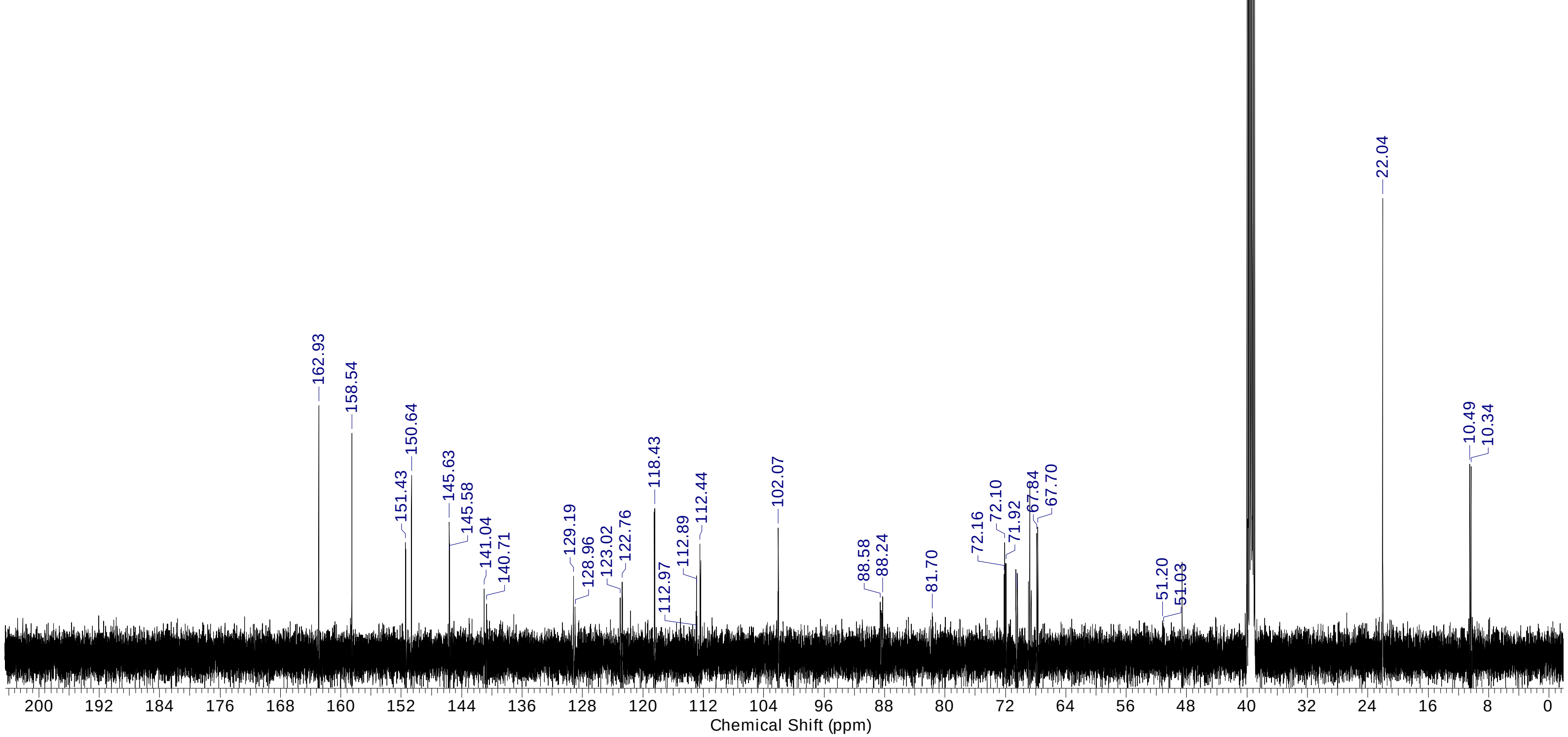

5′-Deoxy-5′-(4′′-(α-hydroxy-2′′′-benzothienylmethyl)triazolo)uridine (29f)

^{1}H NMR

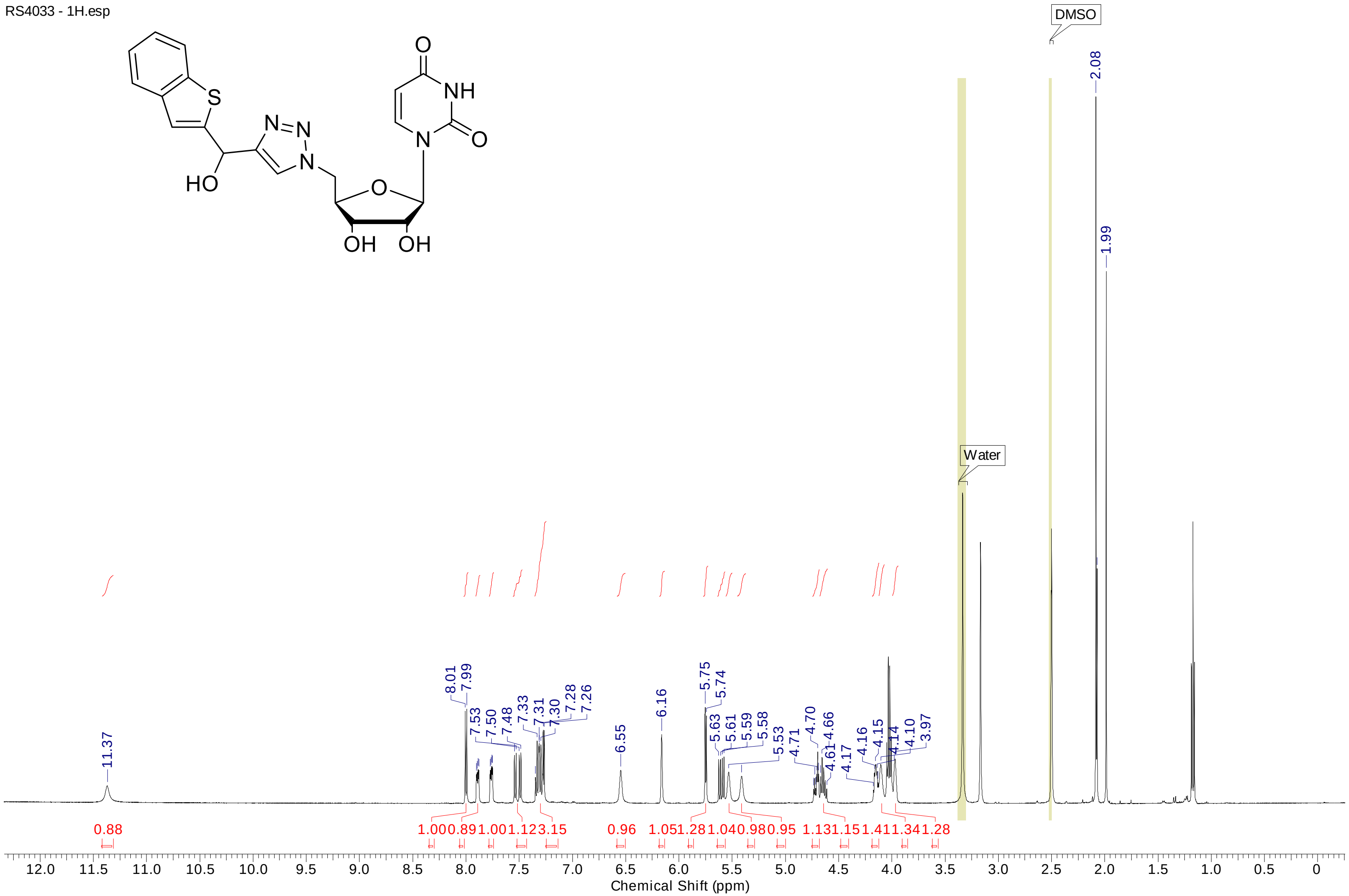

^{13}C NMR

RS4033 - 13C.esp

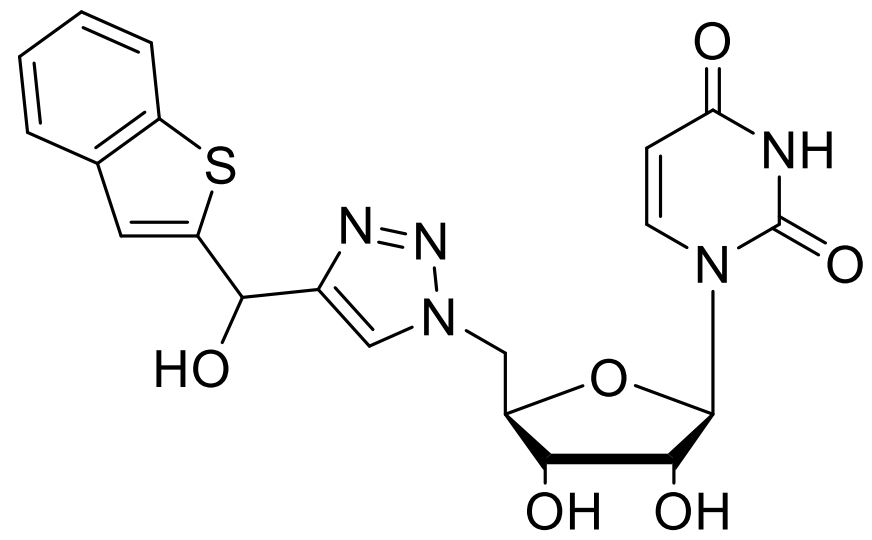

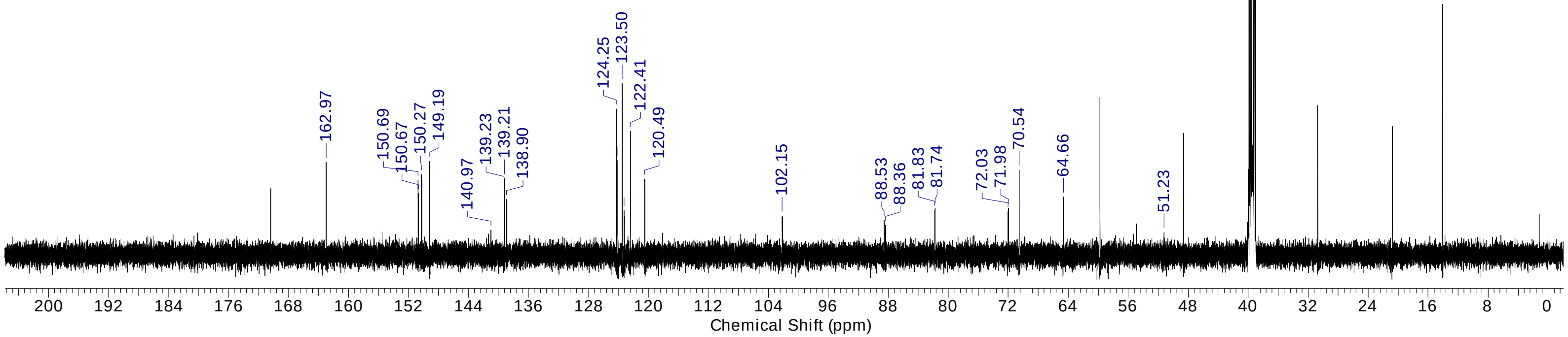

5′-Deoxy-5′-(4′′-(α-hydroxy-α-(*N-tert*-Butoxycarbonylindol-3′′′-ylmethyl)triazolo)uridine (29g)

<u>^{1}H NMR</u>

RS4022 - 1H.esp

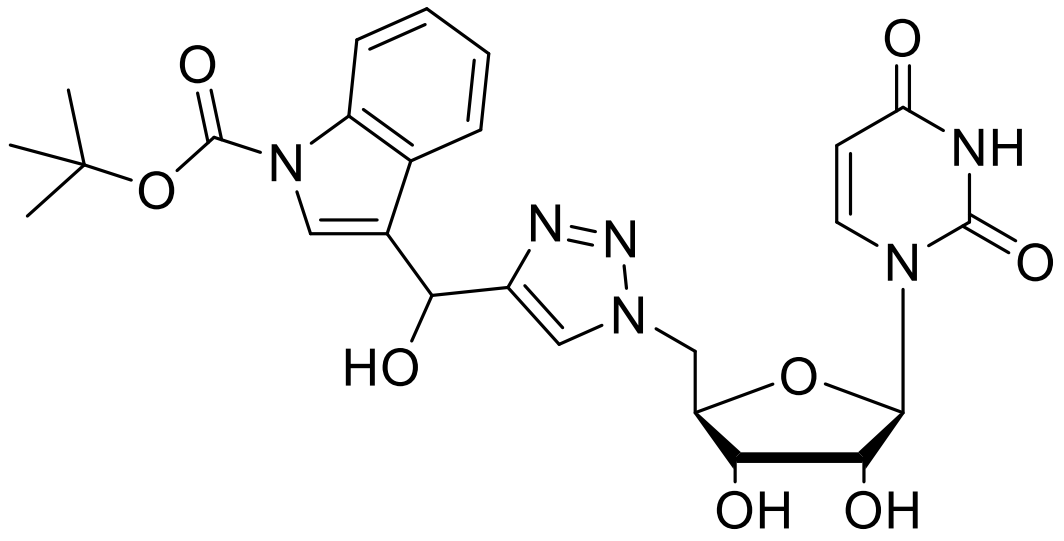

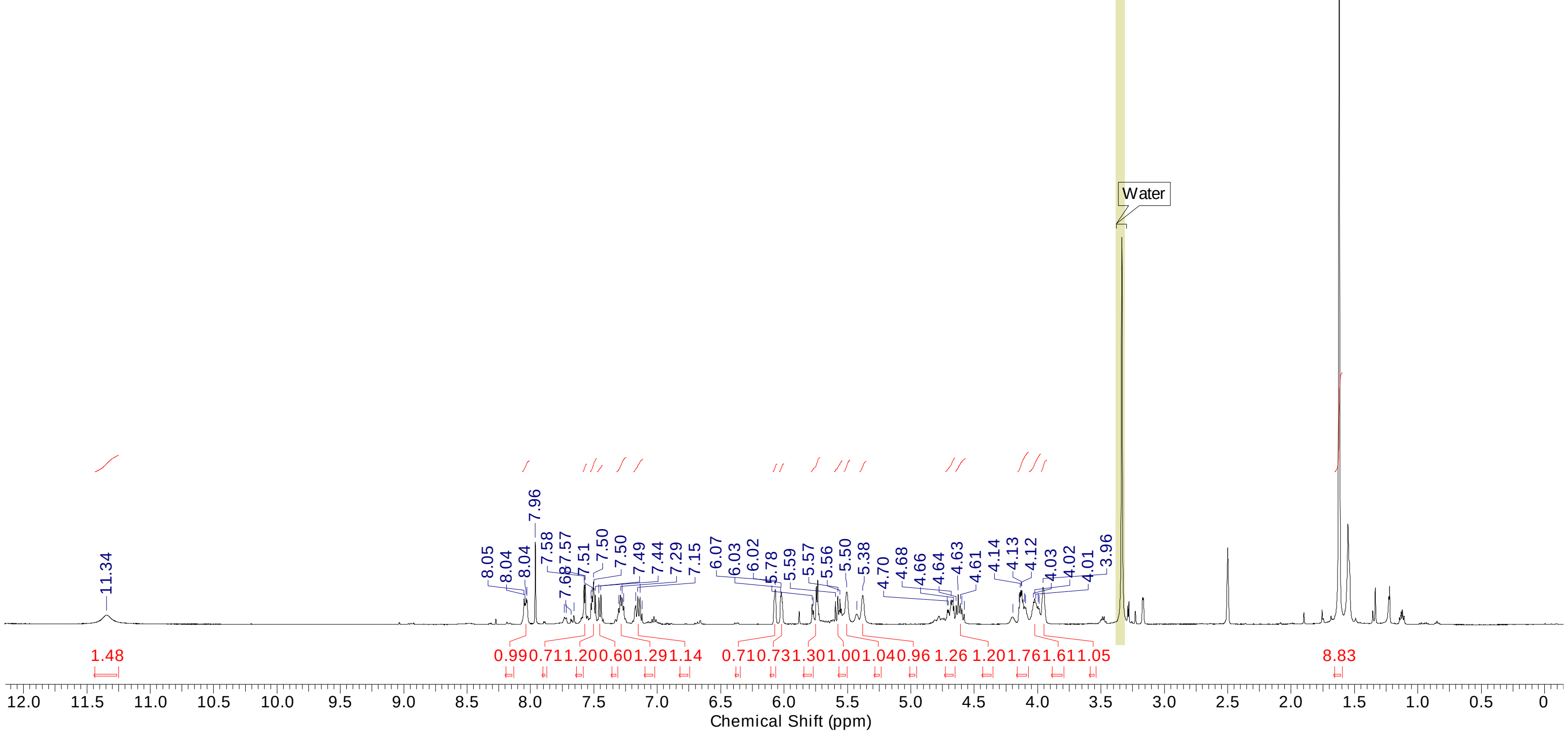

^{13}C NMR

RS4022 - 13C.esp

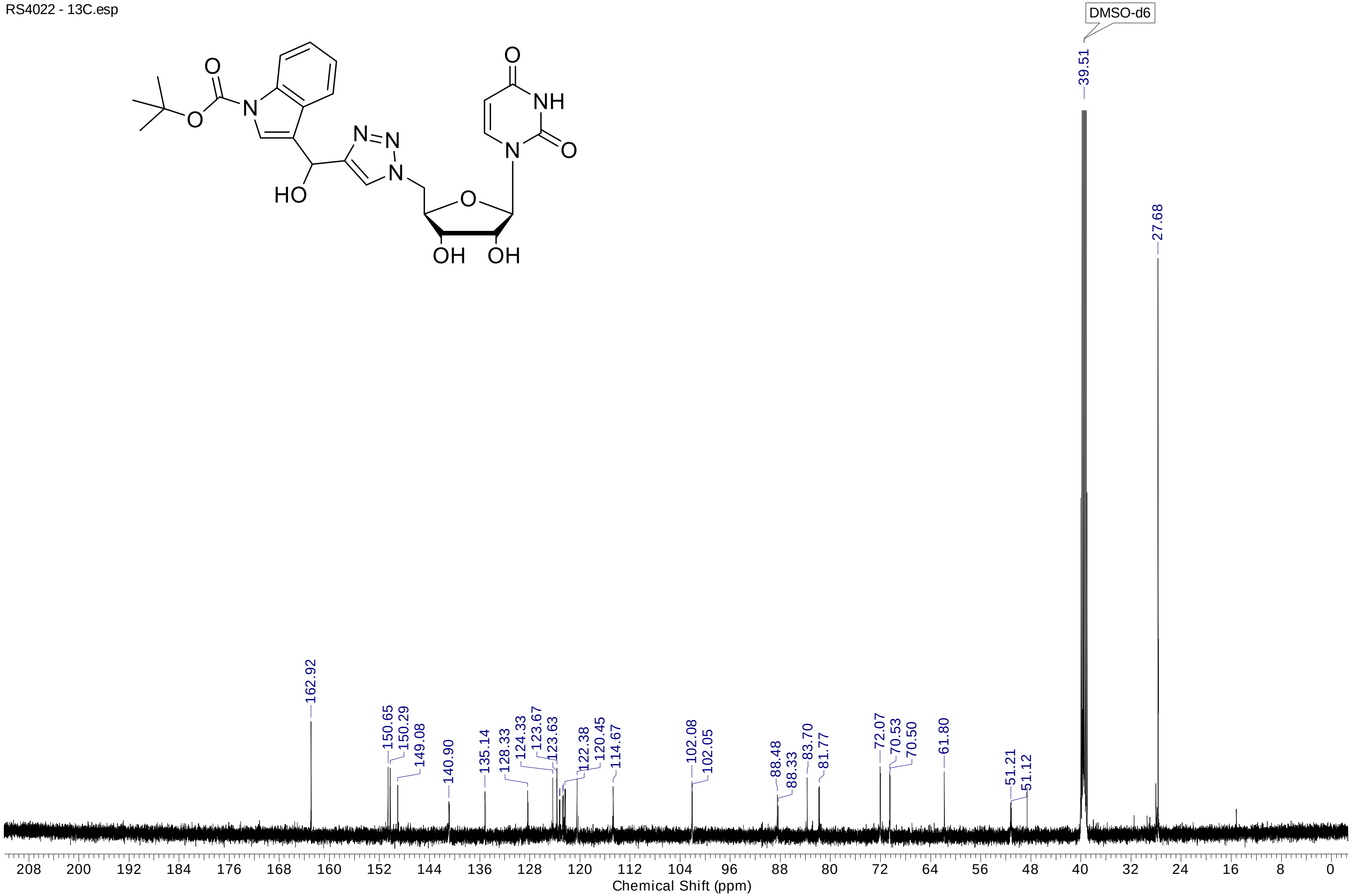

5′-(4″-(*N*-*tert*-Butoxycarbonylindol-3‴-yl)-α-carboxamido-α-hydroxymethyltriazolo)-5′-deoxyuridine (29h)

^{1}H NMR

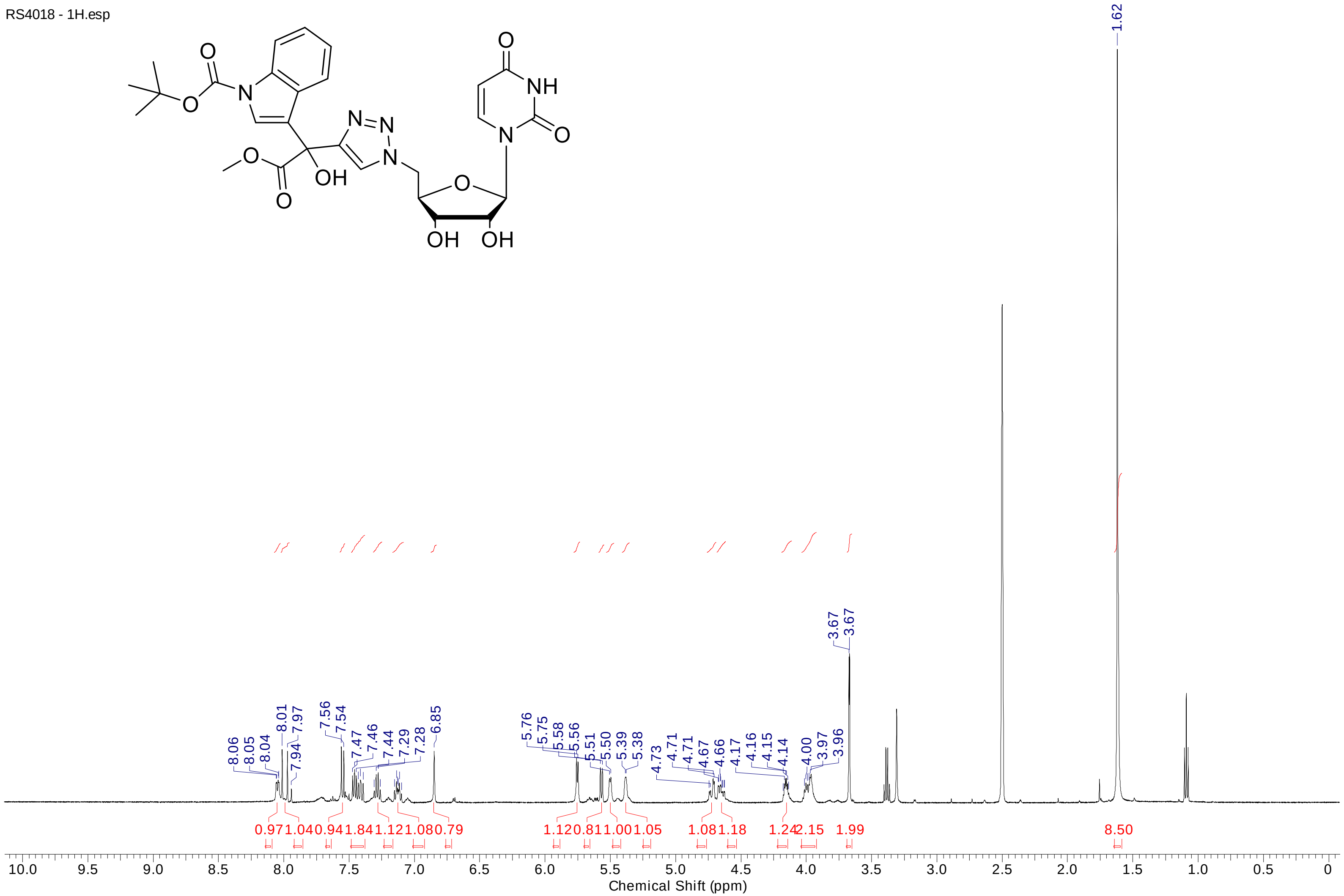

^{13}C NMR

RS4018 - 13C.esp

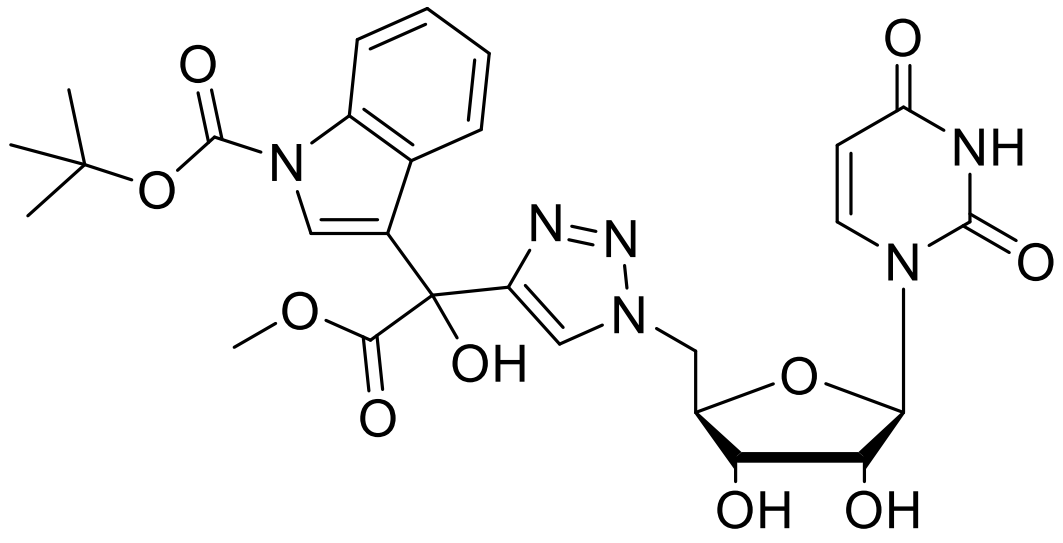

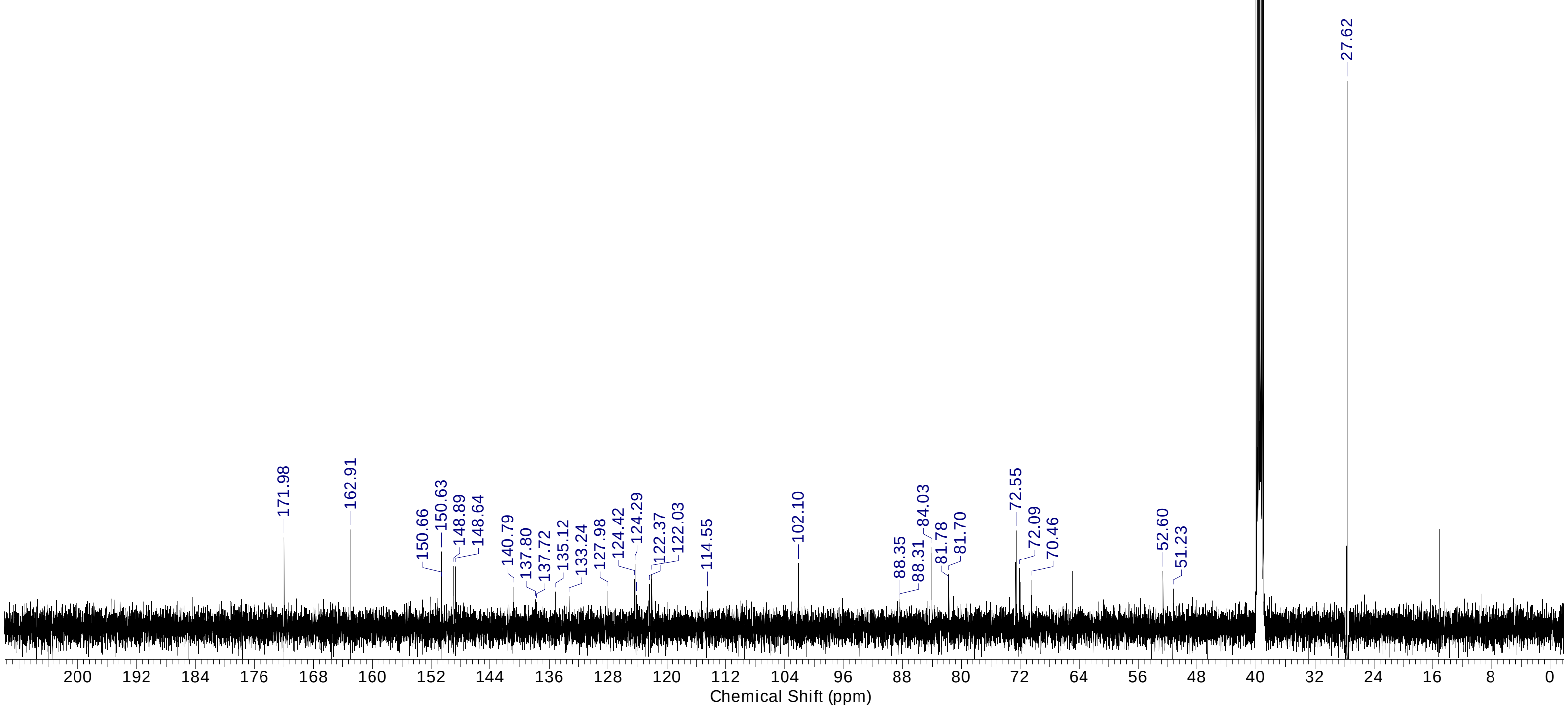

5′-(4′′-(α-Hydroxy-α-trifluoromethylbenzyl)triazolo)-5′-deoxyuridine (29i)

<u>^{1}H NMR</u>

RS4029_1H.esp

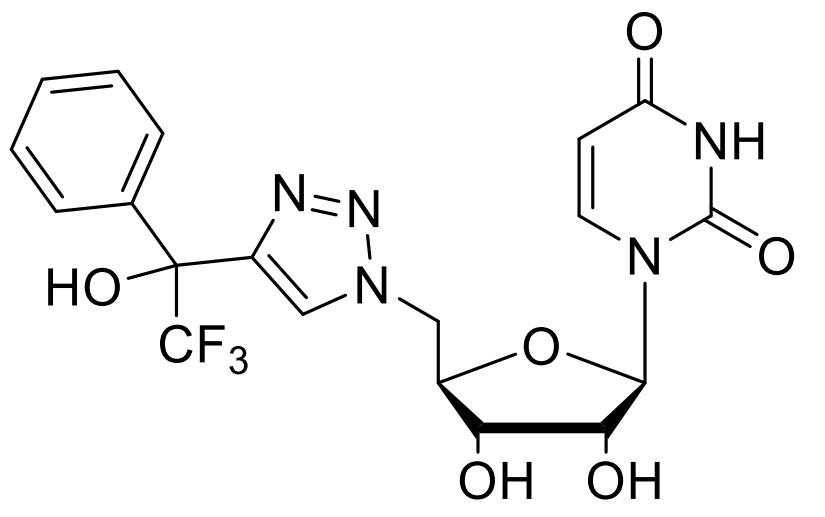

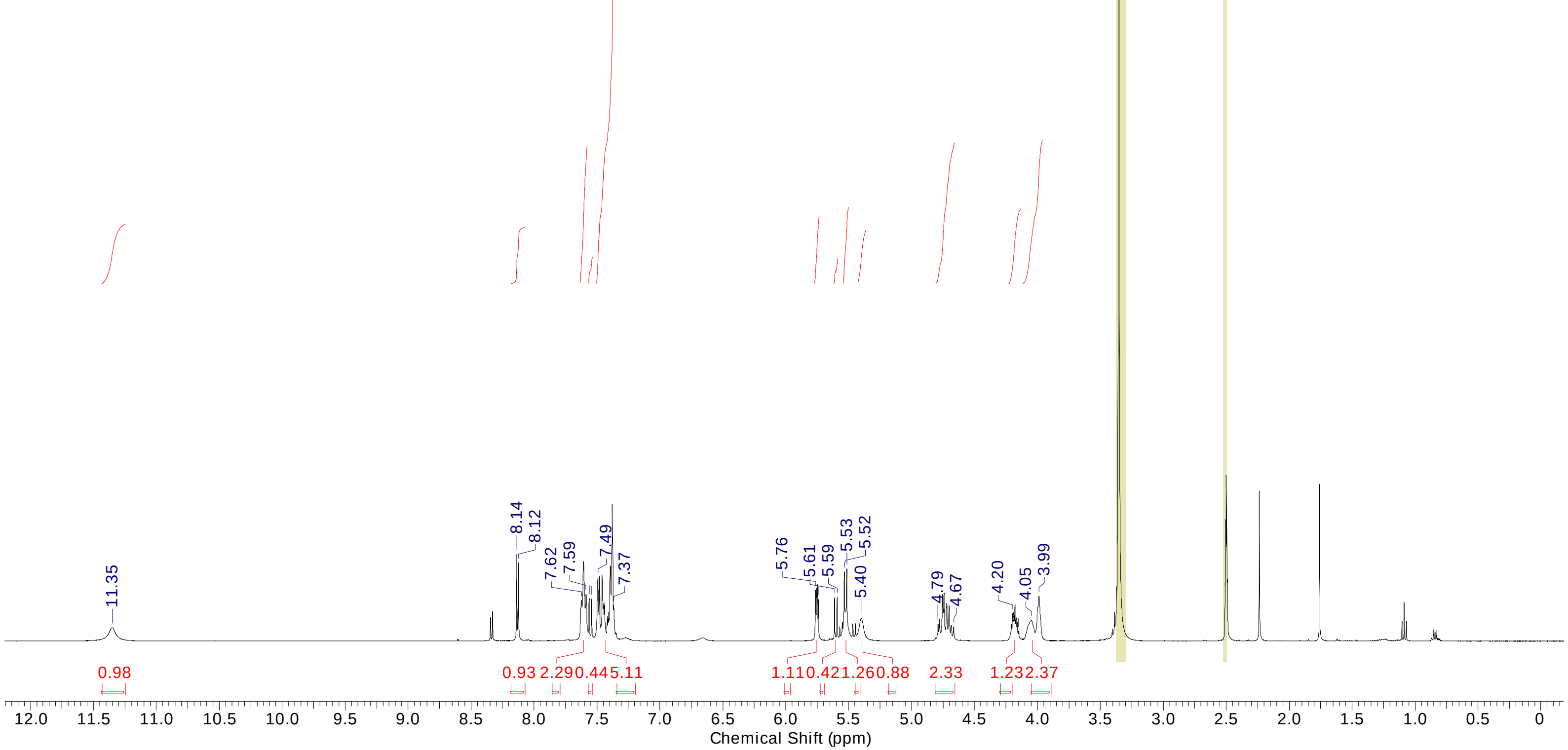

^{13}C NMR

RS4029_13C.esp

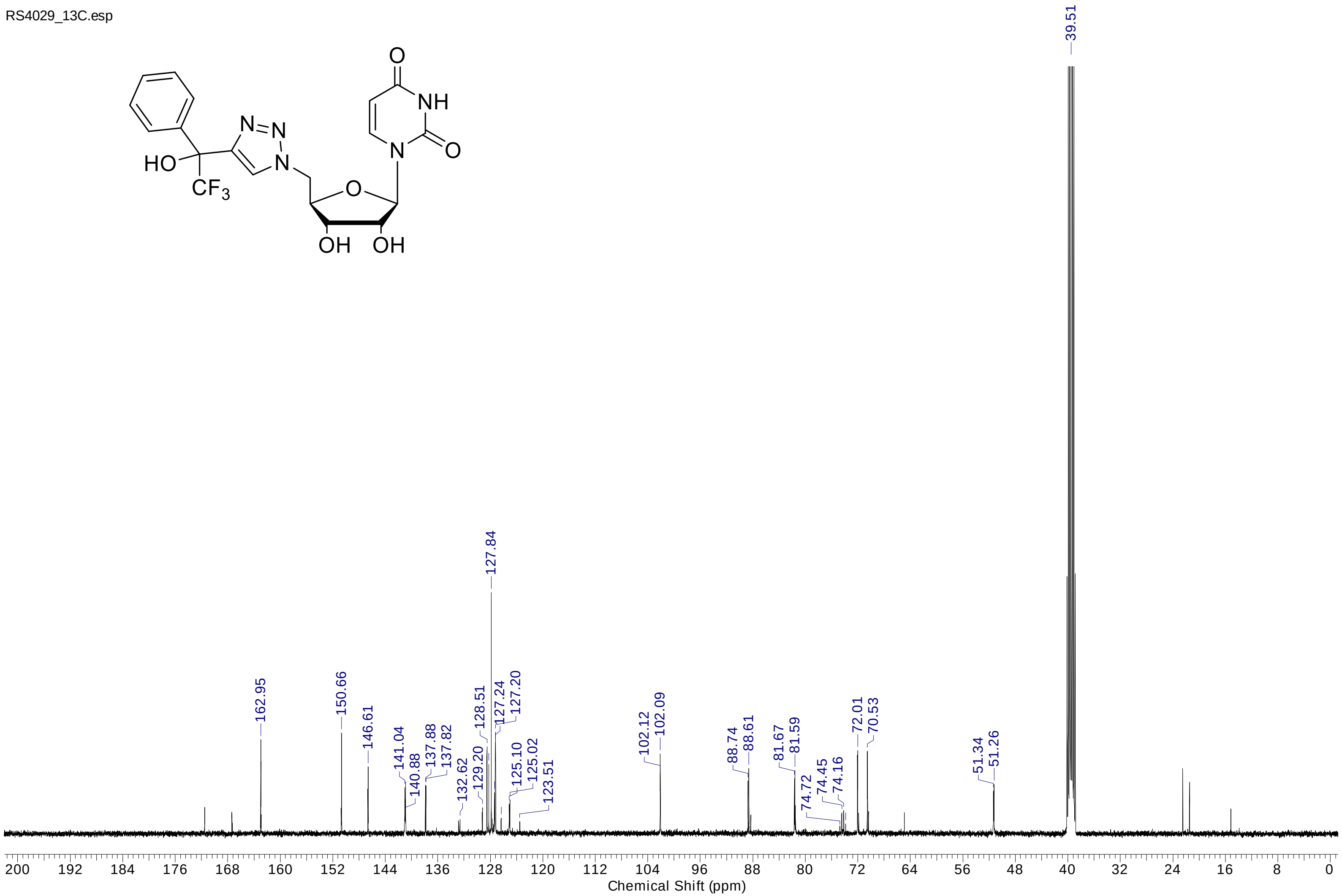

5′-Deoxy-5-flouro -5′-(4′′-(α-hydroxy-3′′′-phenoxybenzyl)triazolo)uridine (31a)

<u>^{1}H NMR</u>

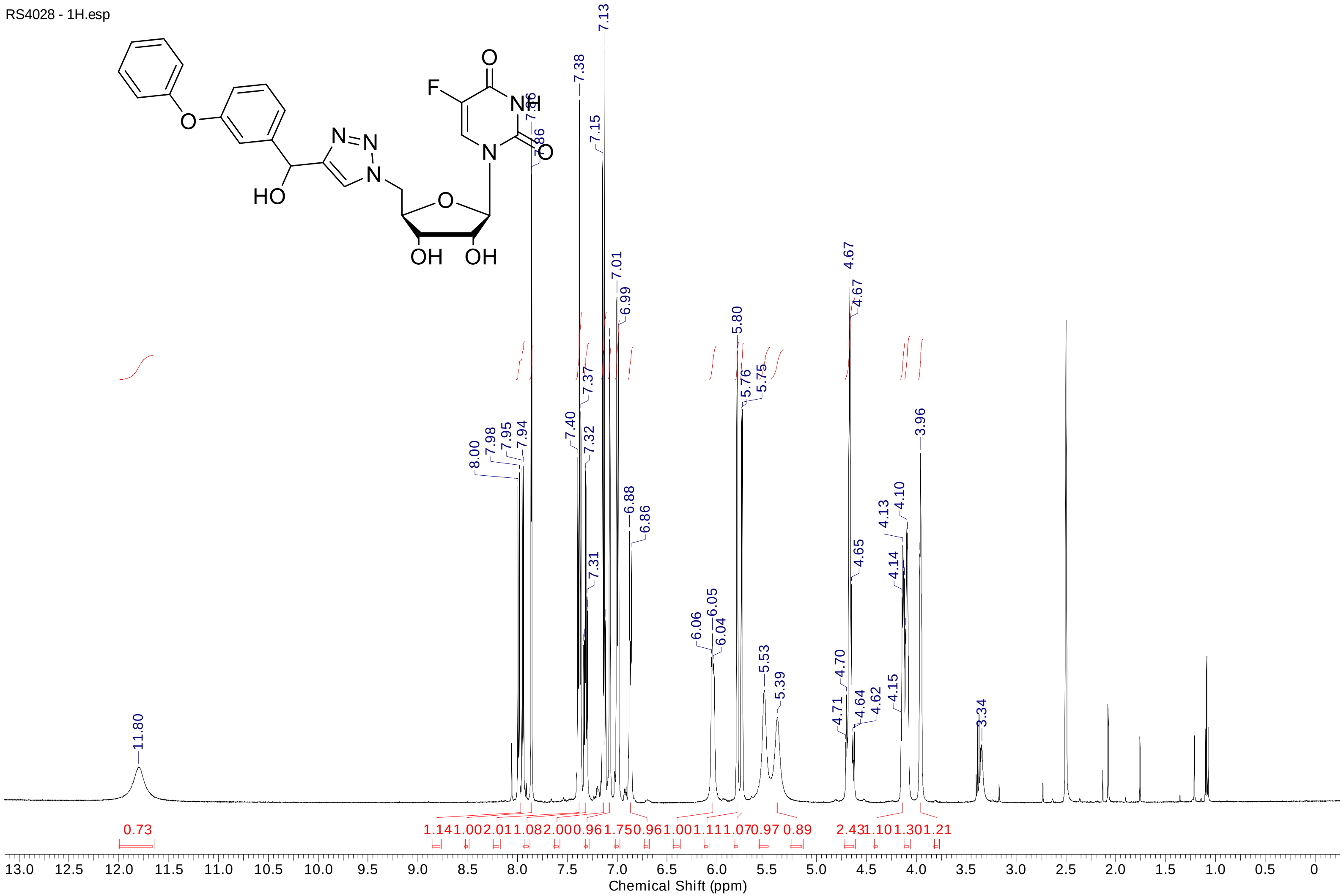

^{13}C NMR

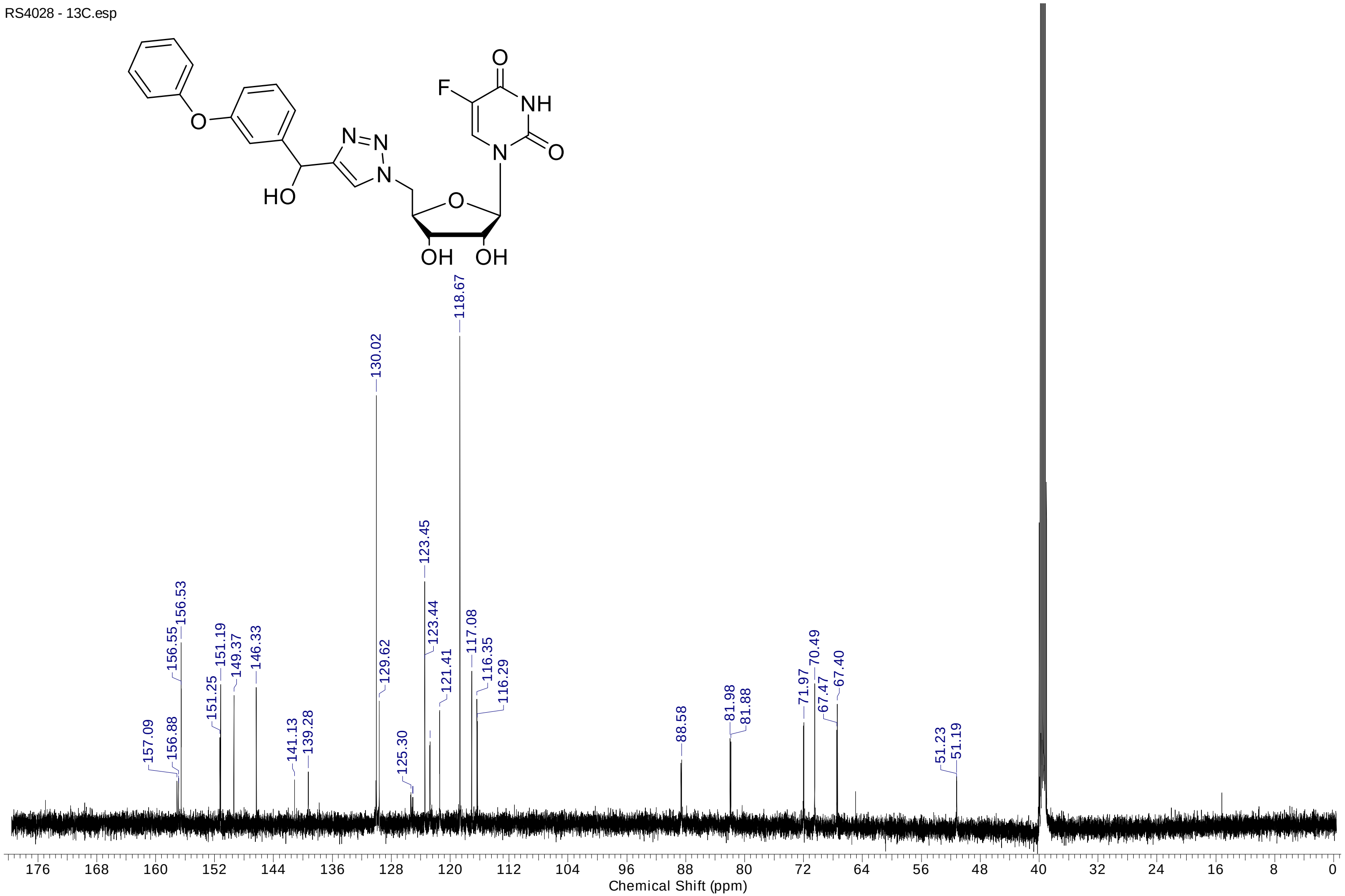
RS4028 - 13C.esp
O
F
NH
N
O
N=N
N
O
HO
O
OH
OH
157.09
156.88
156.55
156.53
151.25
151.19
149.37
146.33
141.13
139.28
130.02
129.62
125.30
123.45
123.44
121.41
118.67
117.08
116.35
116.29
88.58
81.98
81.88
71.97
70.49
67.47
67.40
51.23
51.19
176
168
160
152
144
136
128
120
112
104
96
88
80
72
64
56
48
40
32
24
16
8
0
Chemical Shift (ppm)

5′-deoxy-5′-(4′′-(α-Hydroxy-3′′′-phenoxybenzyl)triazolo)thymidine (31b)

<u>^{1}H NMR</u>

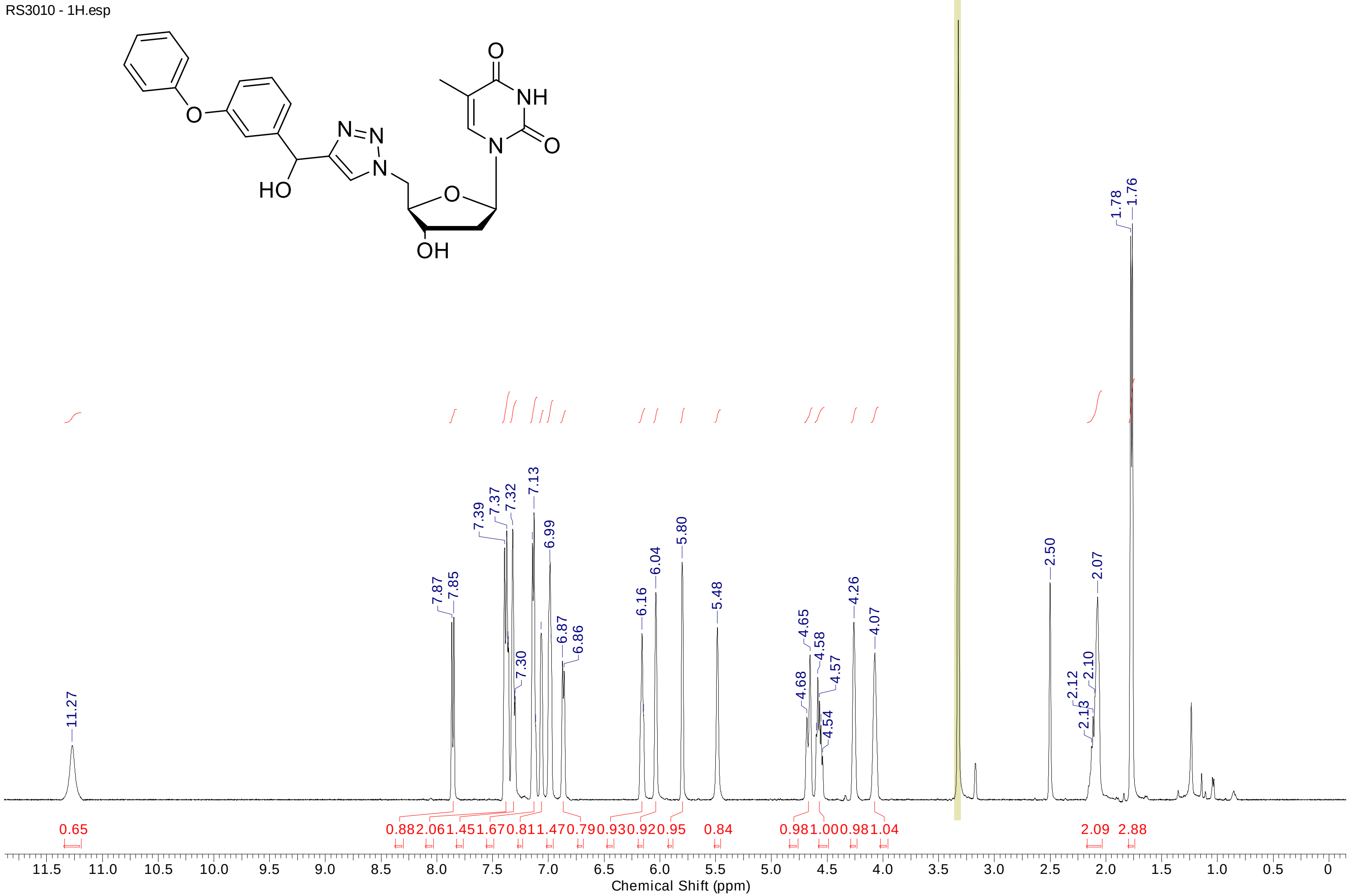

^13^C NMR

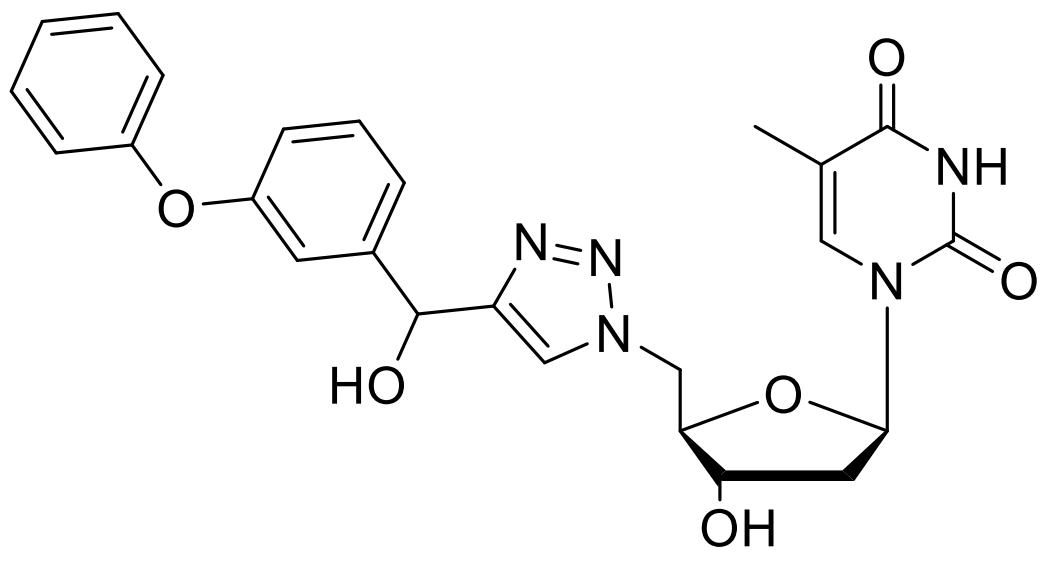

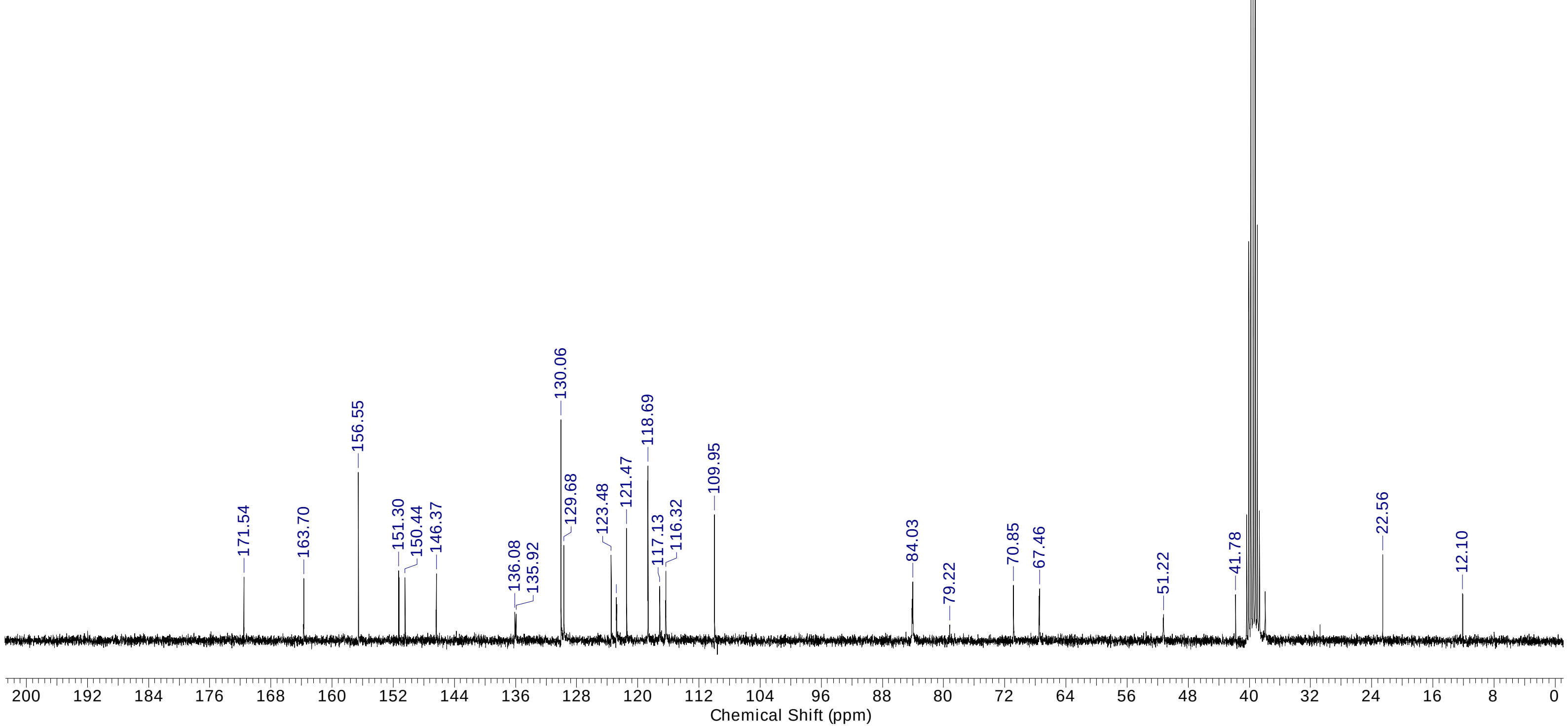
RS3010 - 13C.esp
171.54
163.70
156.55
151.30
150.44
146.37
136.08
135.92
130.06
129.68
123.48
121.47
118.69
117.13
116.32
109.95
84.03
79.22
70.85
67.46
51.22
41.78
22.56
12.10
200 192 184 176 168 160 152 144 136 128 120 112 104 96 88 80 72 64 56 48 40 32 24 16 8 0
Chemical Shift (ppm)

2′, 5′-dideoxy-5′-(4′′-(α-hydroxy-3′′′-phenoxybenzyl)triazolo)uridine (31c)

^{1}H NMR

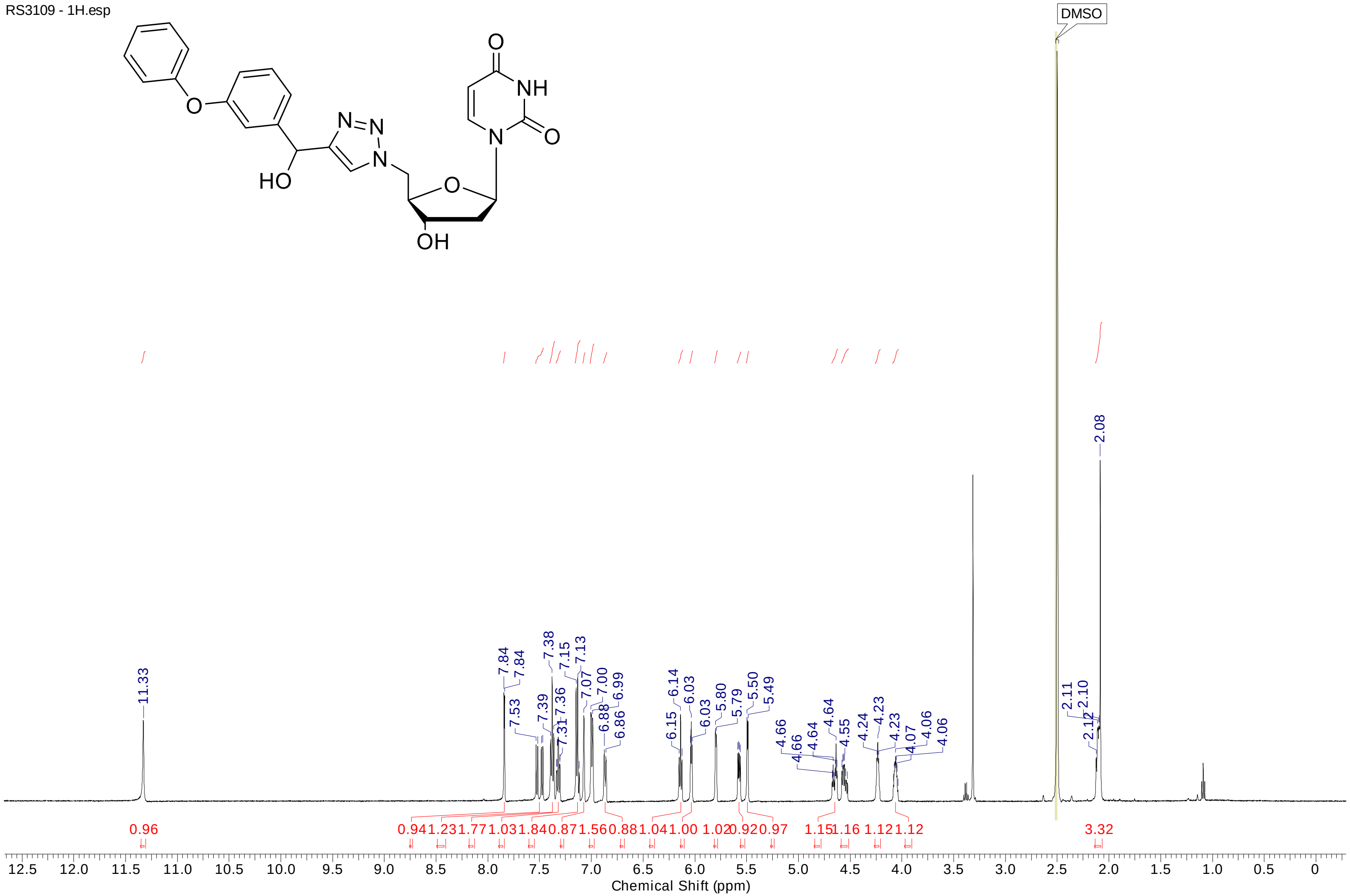

^{13}C NMR

RS3109 - 13C.esp

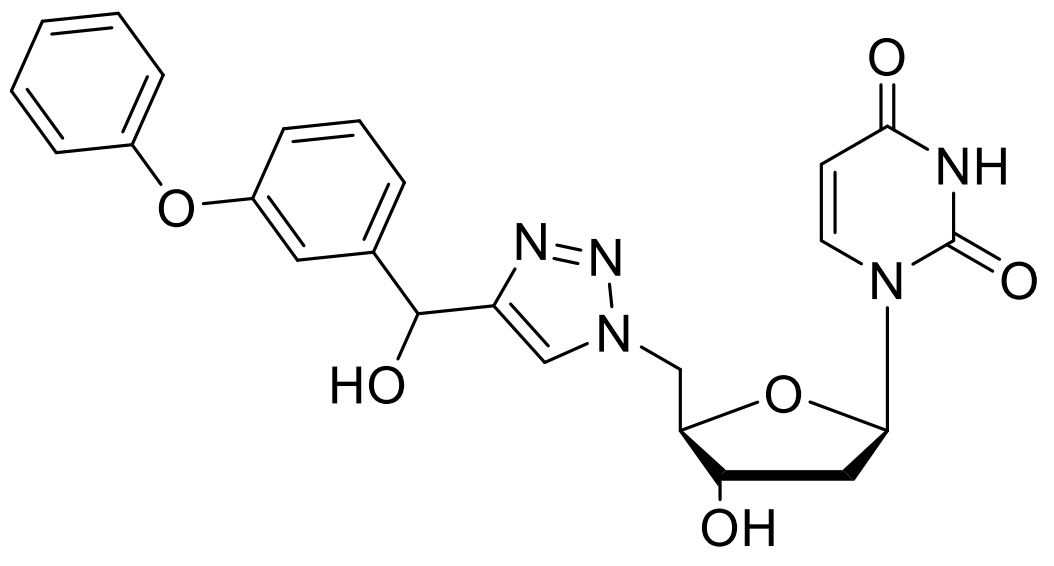

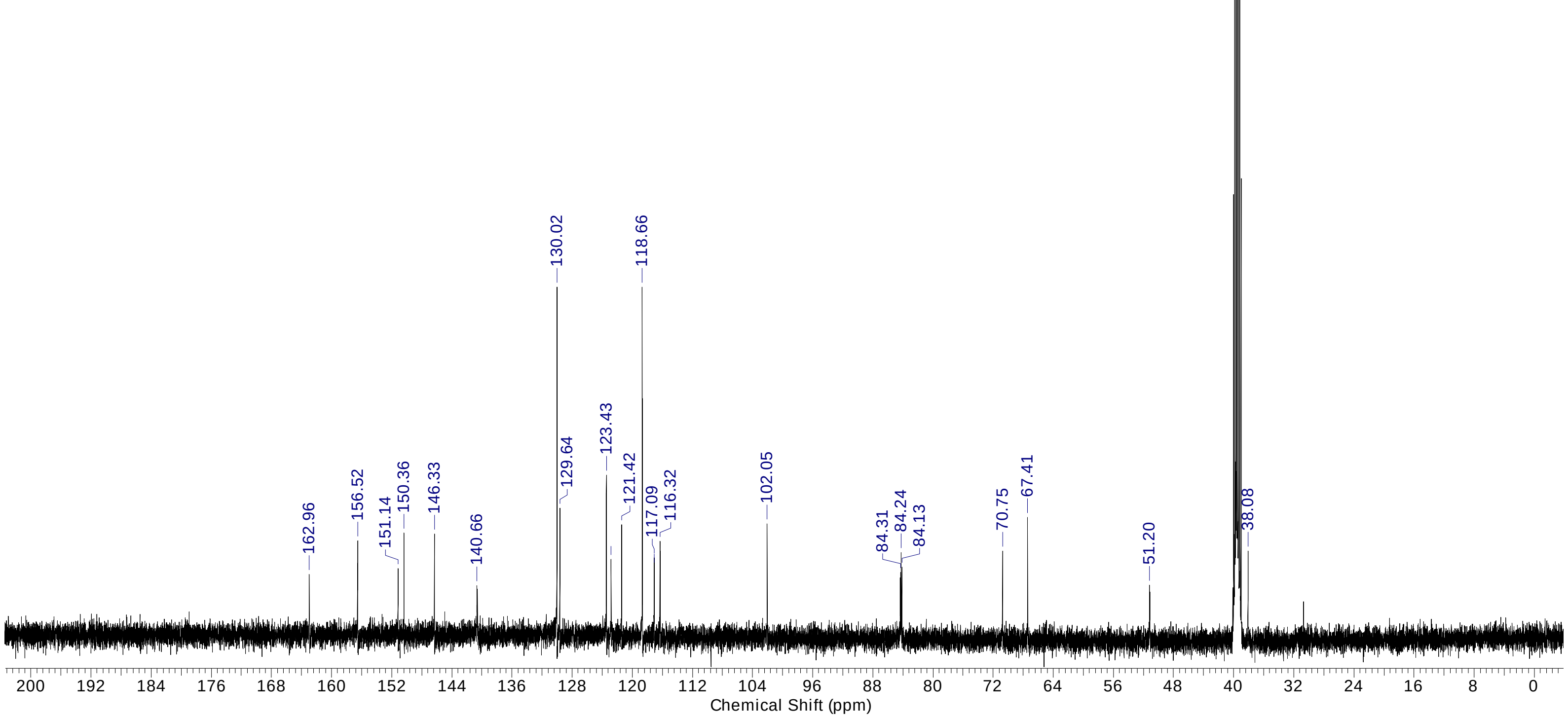

2′, 5′-dideoxy-5′-(4′′-(α-hydroxy-3′′′-phenoxybenzyl)triazolo)2′-fluorouridine (31d)

^{1}H NMR

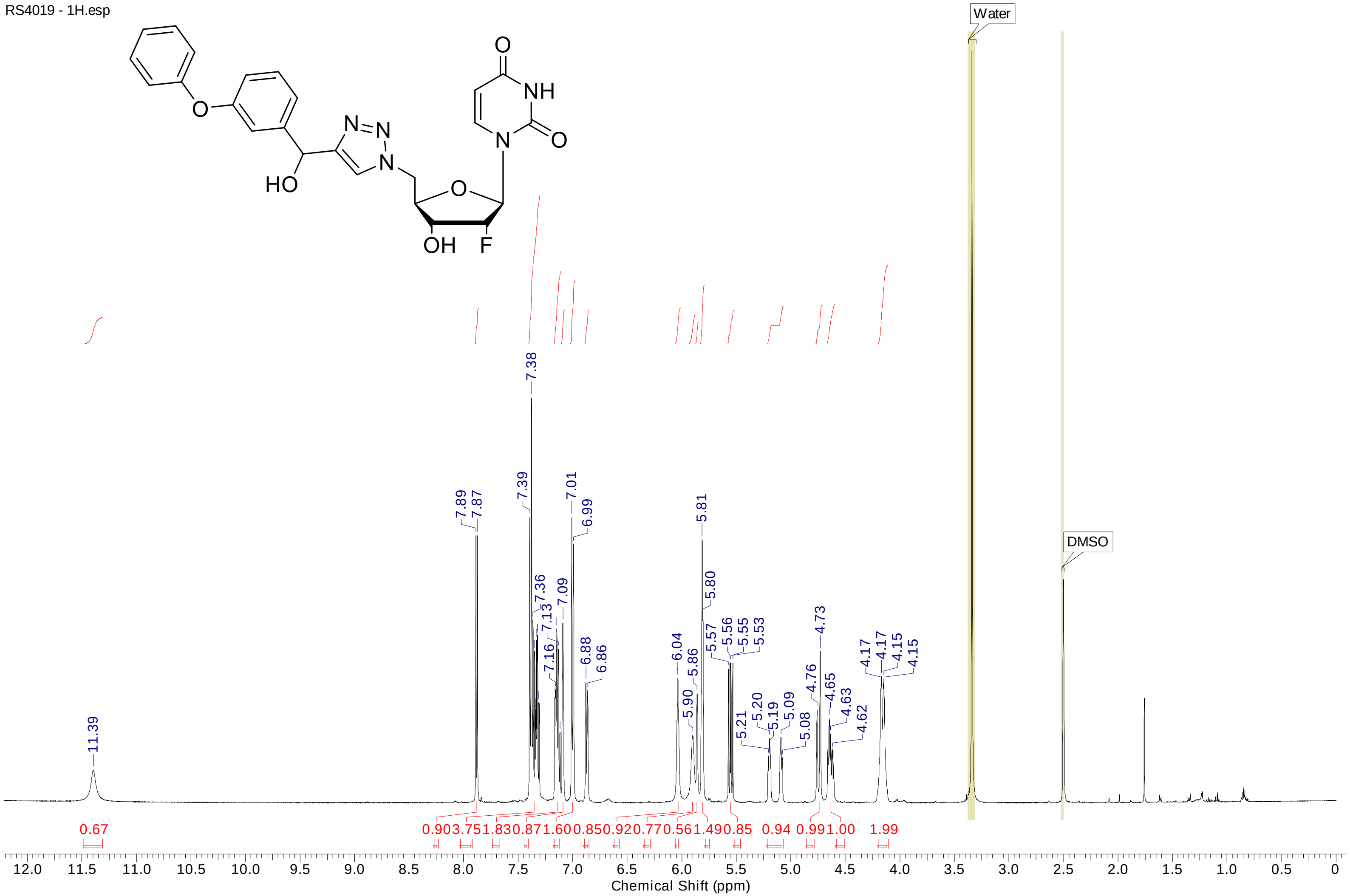

^{13}C NMR

RS4019 - 13C.esp

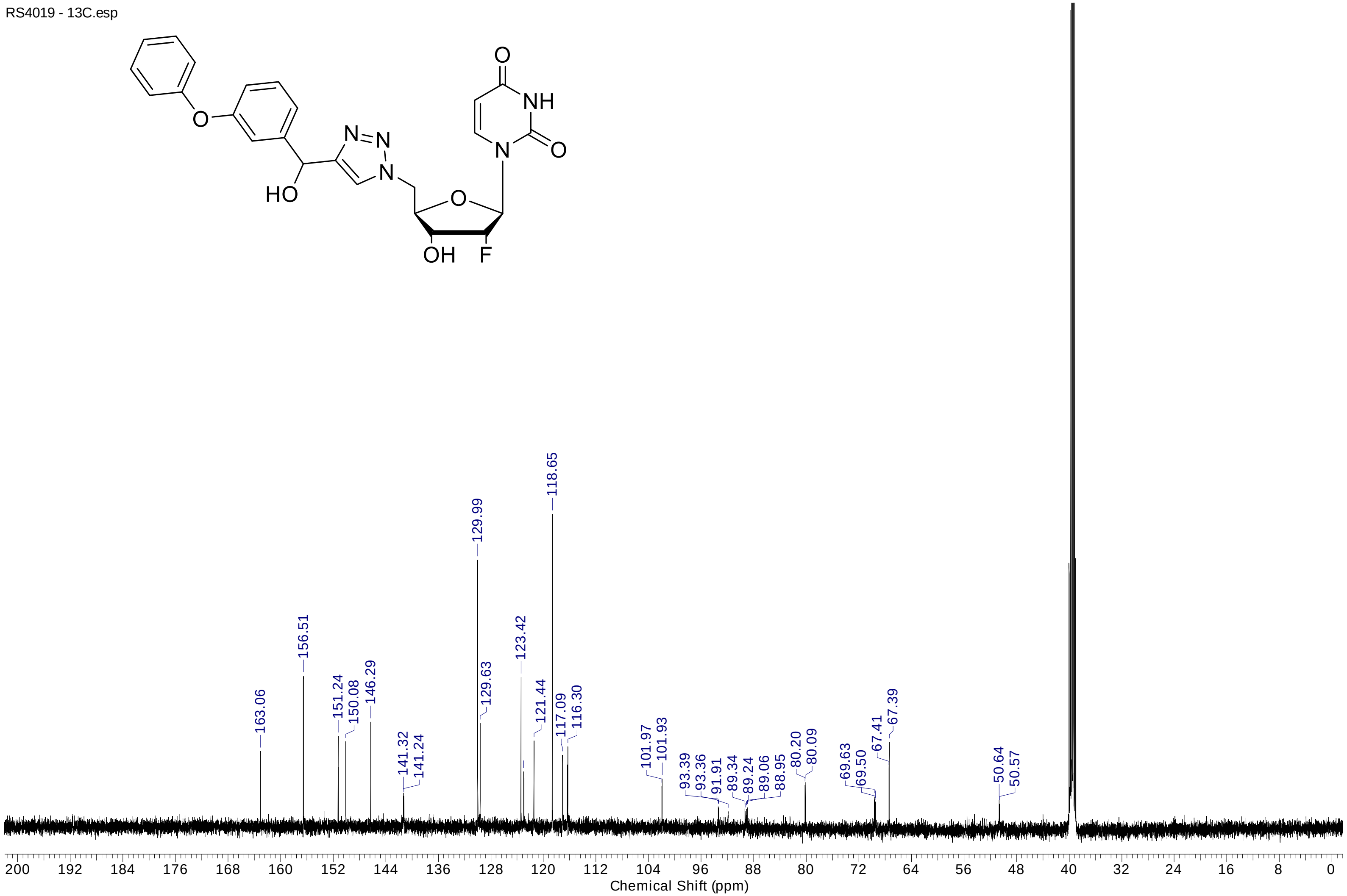

5′-deoxy-5′-(4′′-(α-hydroxy-3′′′-phenoxybenzyl)triazolo)-2′-methoxyuridine (31e)

^{1}H NMR

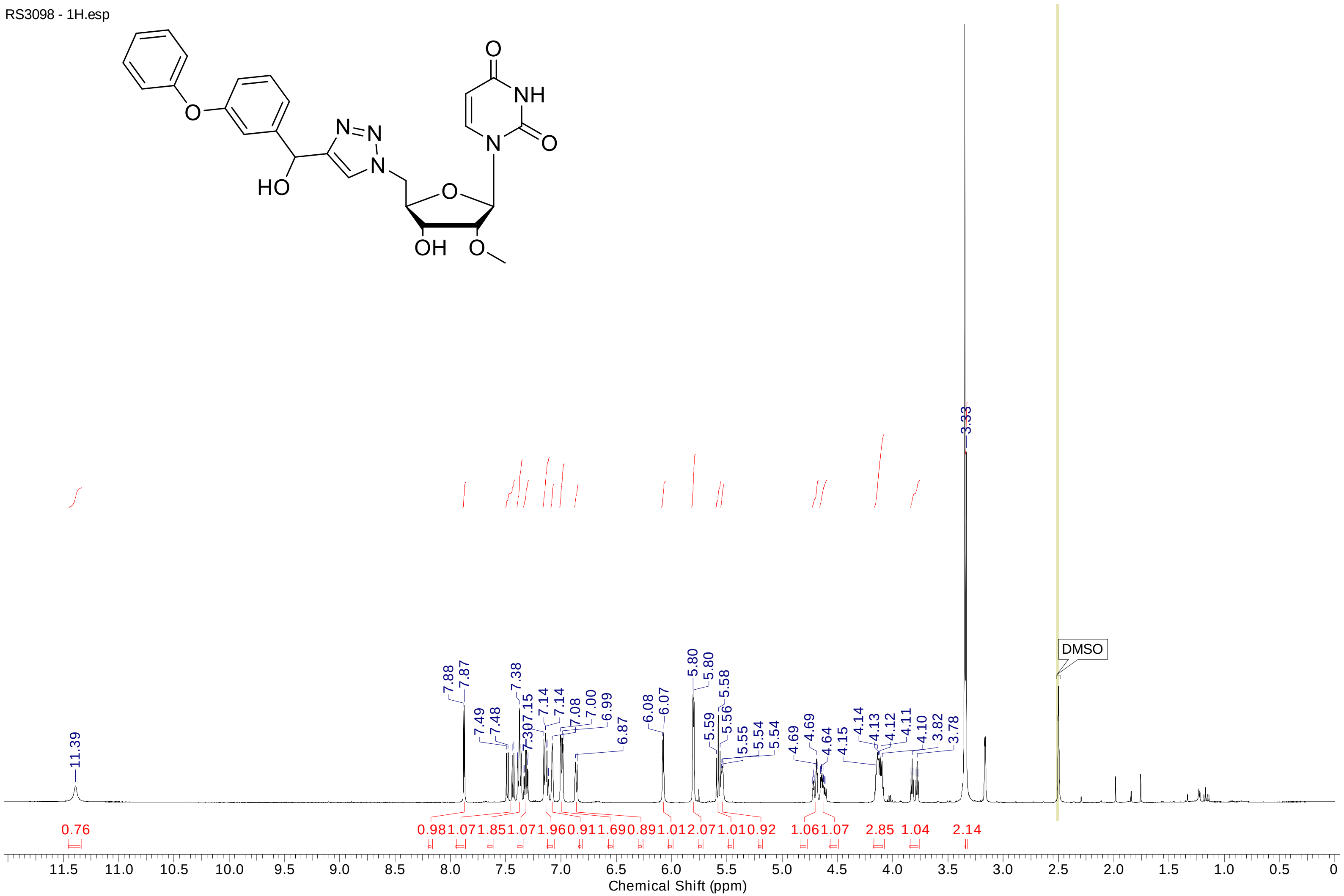

^{13}C NMR

RS3098 - 13C.esp

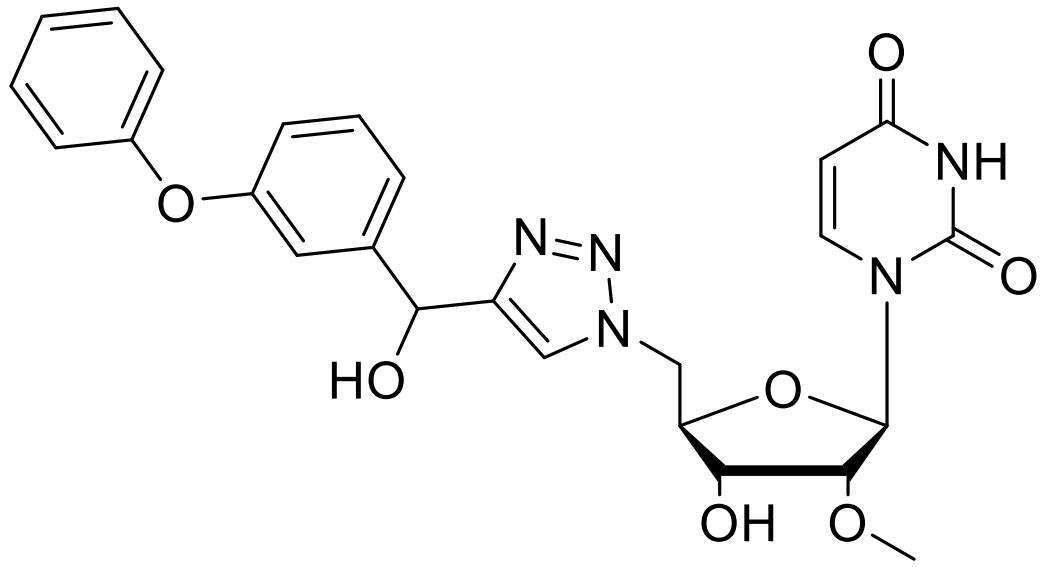

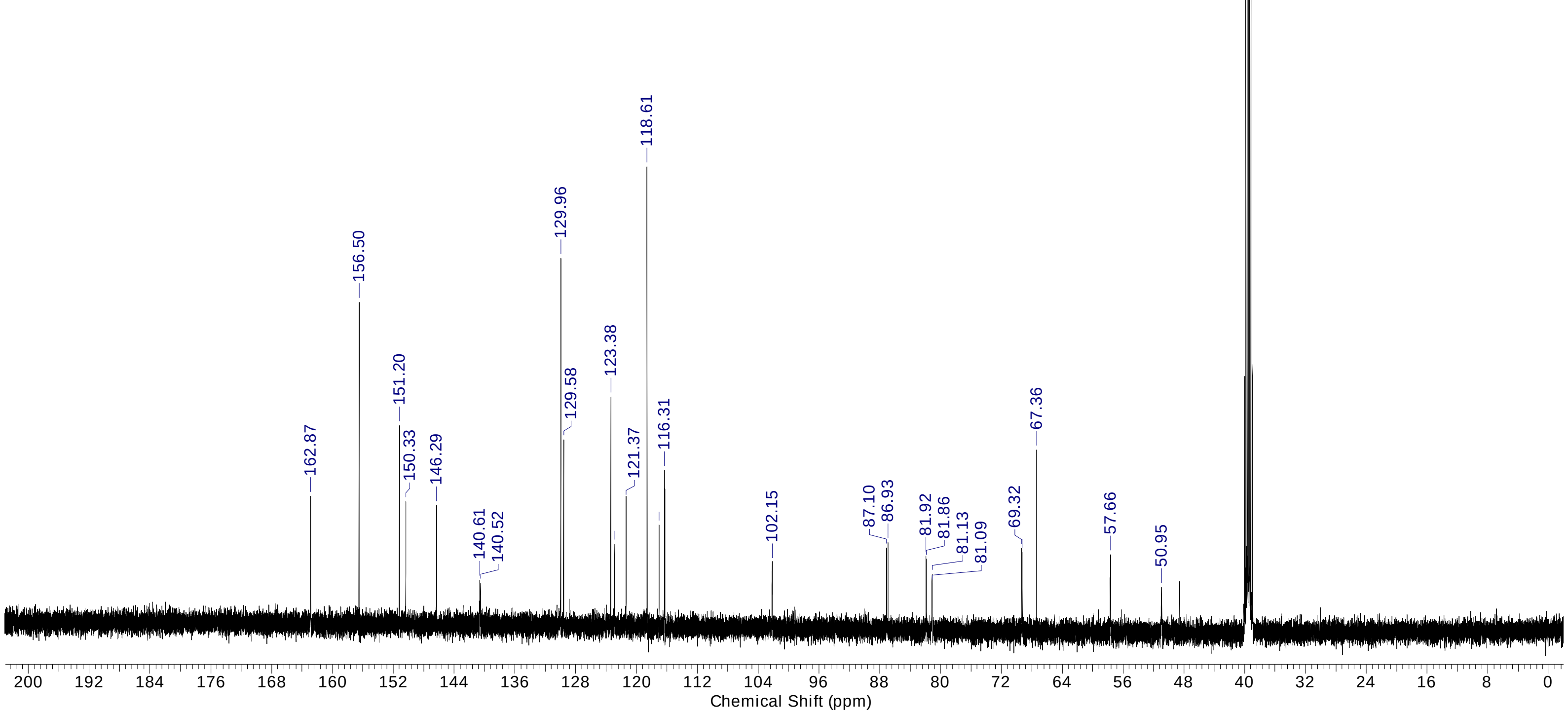

[1] S. Kitazume, Y. Tachida, R. Oka, K. Shirotani, T. C. Saido, Y. Hashimoto, *Proceedings of the National Academy of Sciences of the United States of America* **2001**, *98*, 13554-13559.
[2] N. Ghosh, S. Nayak, A. K. Sahoo, *The Journal of Organic Chemistry* **2011**, *76*, 500-511.
[3] C. Cheng, L. Ge, X. Lu, J. Huang, H. Huang, J. Chen, W. Cao, X. Wu, *Tetrahedron* **2016**, *72*, 6866-6874.
[4] M. J. Ardolino, J. P. Morken, *Journal of the American Chemical Society* **2012**, *134*, 8770-8773.
[5] R. Kourist, S. Bartsch, U. T. Bornscheuer, *Advanced Synthesis & Catalysis* **2007**, *349*, 1393-1398.
[6] A. Daina, O. Michielin, V. Zoete, *Scientific Reports* **2017**, *7*, 42717.
[7] P. Banerjee, E. Kemmler, M. Dunkel, R. Presissner, *Nuclei Acids Research* **2024**, *April (gkae303), ahead of print.*
